# Supplementary material for: Genome-wide association studies and cross-population meta-analyses investigating short and long sleep duration
Source: Nat Commun. 2023 Sep 28;14:6059. doi: 10.1038/s41467-023-41249-y (PMC10539313; doi:10.1038/s41467-023-41249-y)
Supplement: Supplementary file 1 — Supplementary Information [file 41467_2023_41249_MOESM1_ESM.pdf]

Supplementary Material:

Genome-wide association study investigating short and long sleep duration: a cross-population meta-analysis

Isabelle Austin-Zimmerman, Daniel F. Levey, Olga Giannakopoulou, Joseph D. Deak, Marco Galimberti, Keyrun Adhikari, Hang Zhou, Spiros Denaxas, Haritz Irizar, Karoline Kuchenbaecker, Andrew McQuillin, the Million Veteran Program, John Concato, Daniel J. Buysse, J. Michael Gaziano, Daniel J. Gottlieb, Renato Polimanti, Murray B. Stein, Elvira Bramon, and Joel Gelernter.

Table of Contents

1. Results from primary studies 2
Primary studies: UK Biobank 2
European-ancestry population 2
African-ancestry population 3
East Asian-ancestry population 4
Admixed American population 5
Primary studies: MVP 6
European-ancestry population 6
African-ancestry population 7
East Asian-ancestry population 8
Admixed American population 9
Ancestry-specific meta-analyses 10
European-ancestry population 10
African-ancestry population 16
2. Case-case analysis 18
3. Analysis with nightshift workers excluded 29
4. Sex-stratified analysis 30
5. Comparison of results between primary EUR analyses and sensitivity analyses 31
Genetic correlation between main analyses 31
Forest plots of genome-wide significant associations 32
6. Ingenuity Pathways Analysis: Results 41
7. Replication of previously published significant associations in the MVP sample 42

1. Results from primary studies

Primary studies: UK Biobank

European-ancestry population

Supplementary Figure 1. Mirrored Manhattan plot showing genetic associations with short sleep duration (top) and long sleep duration (bottom) in European UK Biobank participants. Loci reaching a nominal threshold of $1x10^{-5}$ highlighted in green, and the red line indicating genome-wide significance threshold of $5x10^{-8}$.

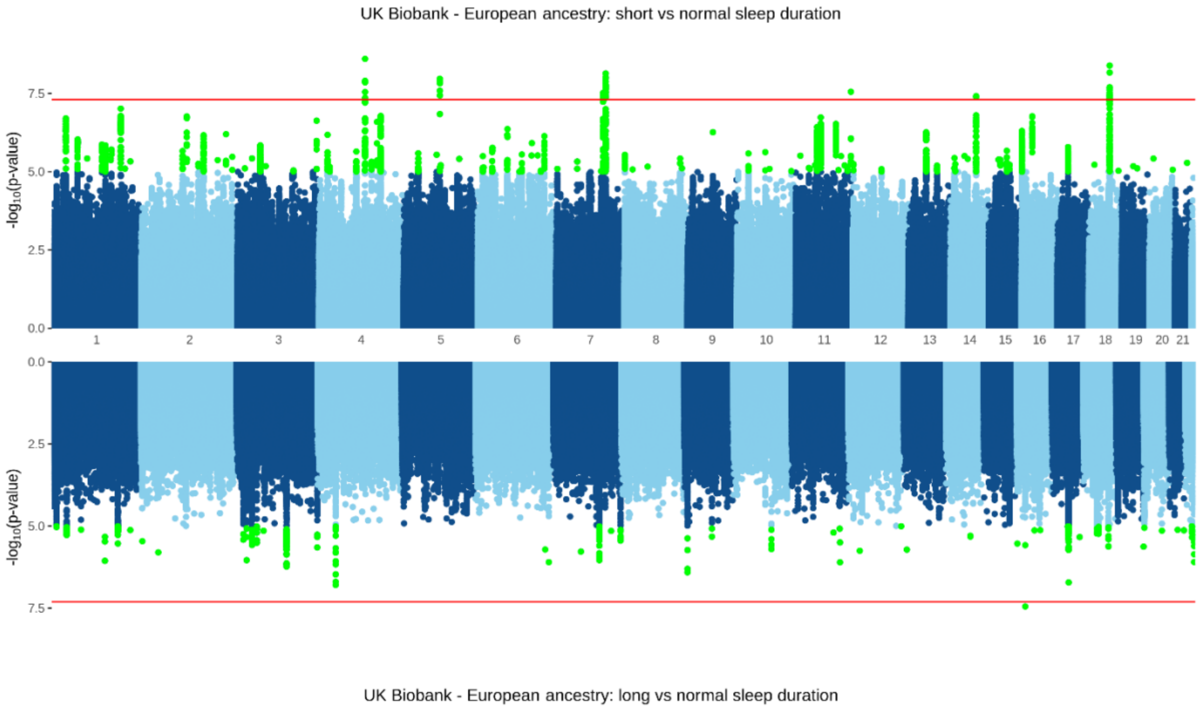

Supplementary Figure 2. Quantile-quantile plot for short sleep duration (left) and long sleep duration (right) in the European UK Biobank sample.

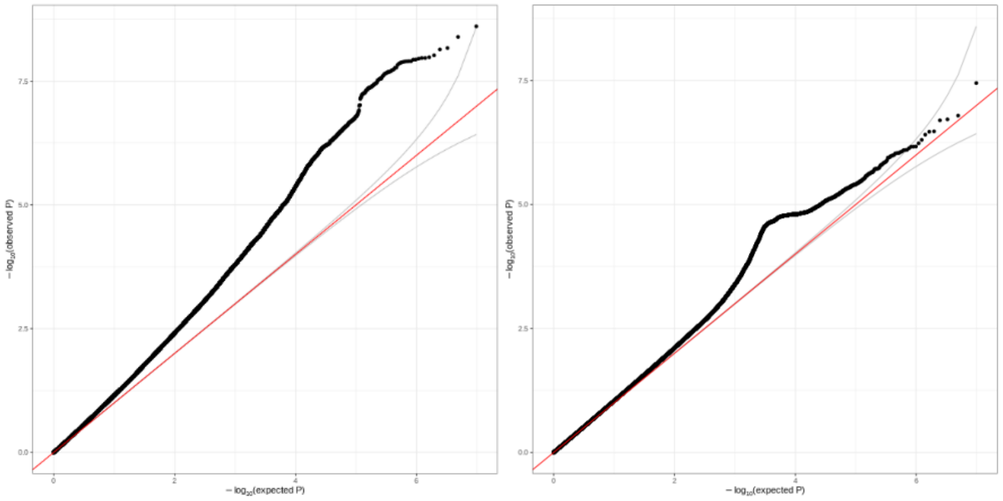

African-ancestry population

Supplementary Figure 3. Mirrored Manhattan plot showing genetic associations with short sleep duration (top) and long sleep duration (bottom) in African UK Biobank participants. Loci reaching a nominal threshold of $1x10^{-5}$ highlighted in green, and the red line indicating genome-wide significance threshold of $5x10^{-8}$.

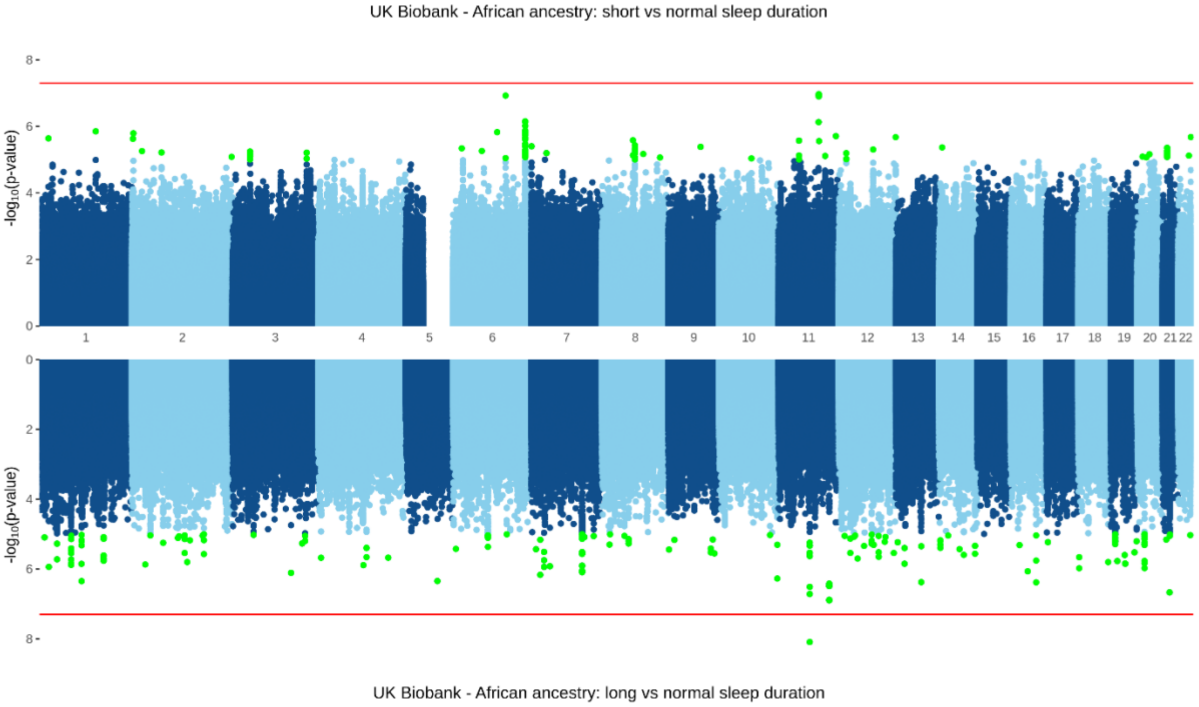

Supplementary Figure 4. Quantile-quantile plot for short sleep duration (left) and long sleep duration (right) in African UK Biobank participants.

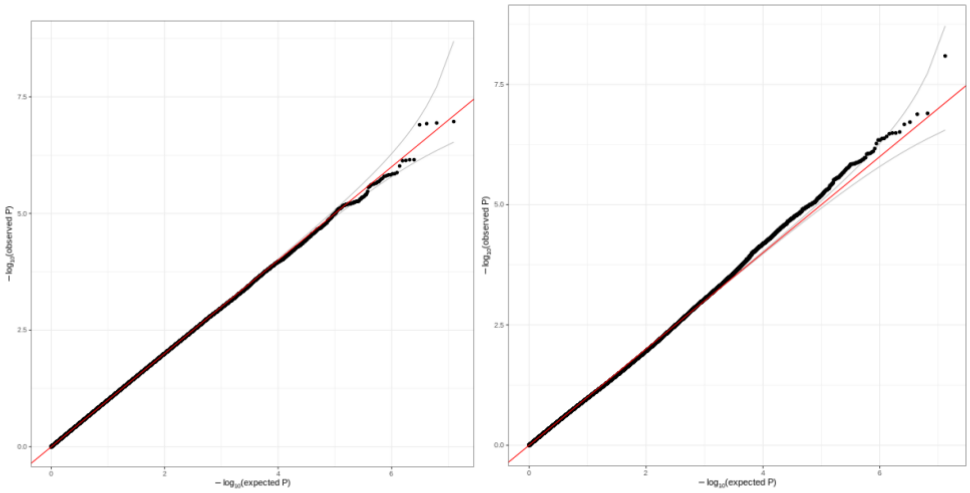

East Asian-ancestry population

Supplementary Figure 5. Mirrored Manhattan plot showing genetic associations with short sleep duration (top) and long sleep duration (bottom) in East Asian UK Biobank participants. Loci reaching a nominal threshold of $1x10^{-5}$ highlighted in green, and the red line indicating genome-wide significance threshold of $5x10^{-8}$.

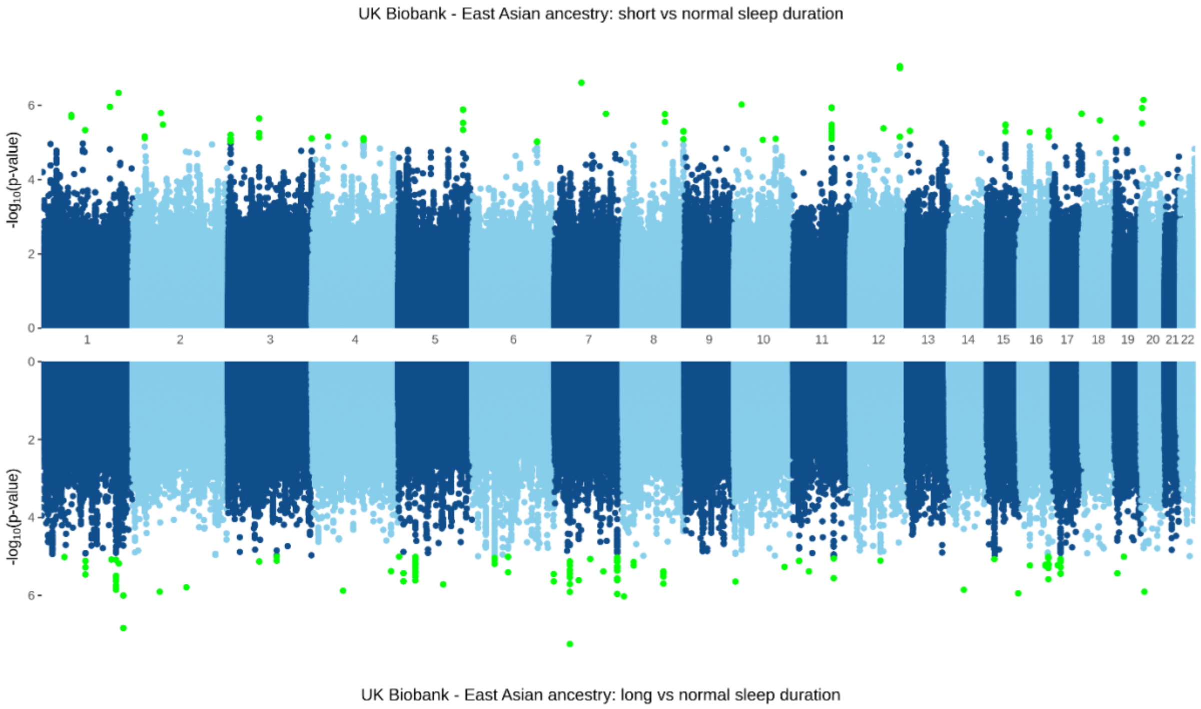

Supplementary Figure 6. Quantile-quantile plot for short sleep duration (left) and long sleep duration (right) in East Asian UK Biobank participants.

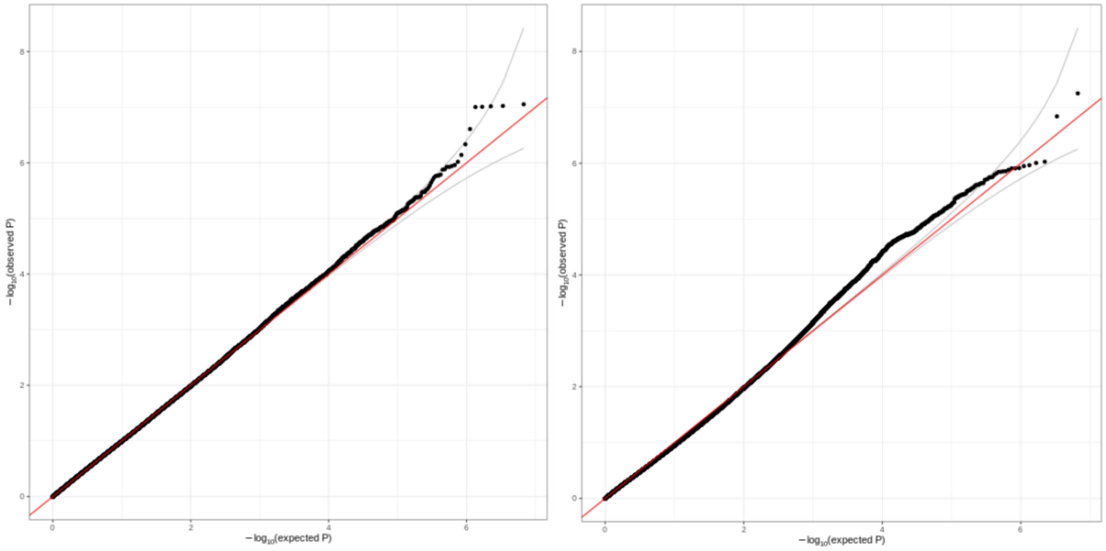

Admixed American population

Supplementary Figure 7. Mirrored Manhattan plot showing genetic associations with short sleep duration (top) and long sleep duration (bottom) in Admixed UK Biobank participants. Loci reaching a nominal threshold of $1x10^{-5}$ highlighted in green, and the red line indicating genome-wide significance threshold of $5x10^{-8}$.

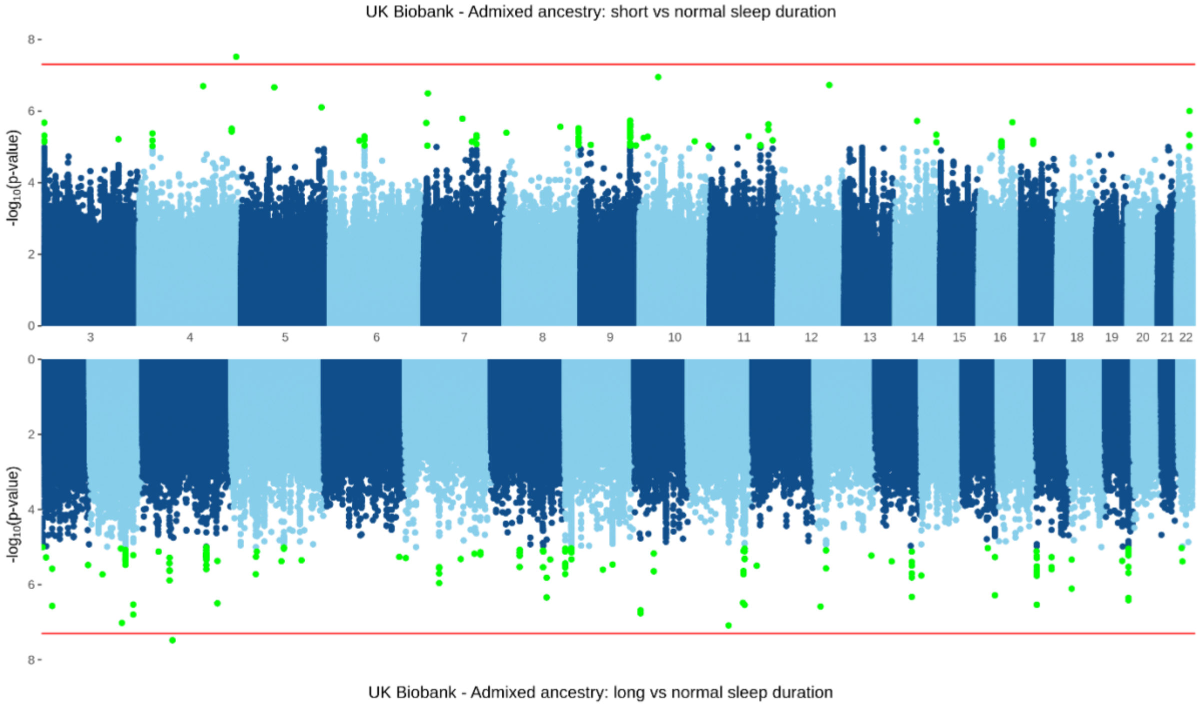

Supplementary Figure 8. Quantile-quantile plot for short sleep duration (left) and long sleep duration (right) in Admixed UK Biobank participants.

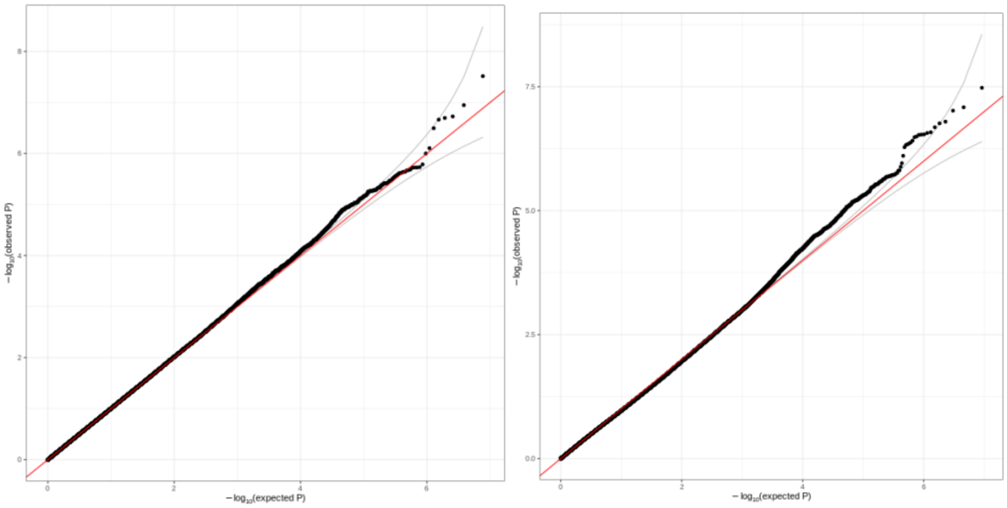

Primary studies: MVP

European-ancestry population

Supplementary Figure 9. Mirrored Manhattan plot showing genetic associations with short sleep duration (top) and long sleep duration (bottom) in European MVP participants. Loci reaching a nominal threshold of $1x10^{-5}$ highlighted in green, and the red line indicating genome-wide significance threshold of $5x10^{-8}$.

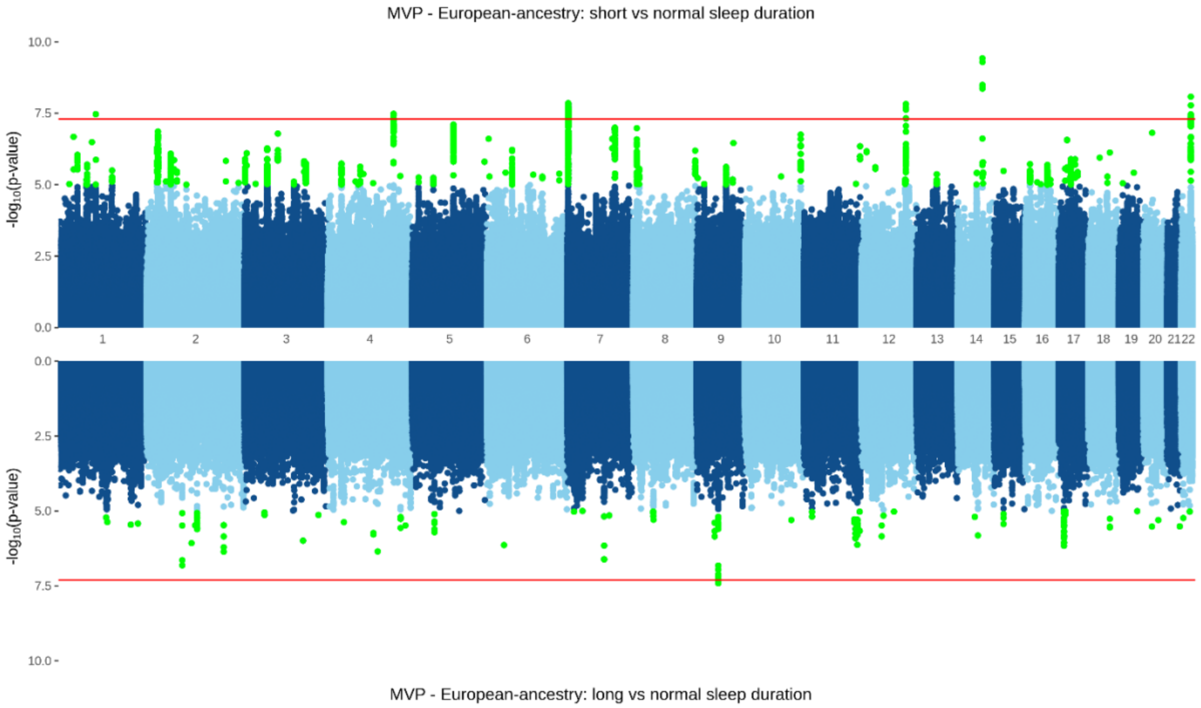

Supplementary Figure 10. Quantile-quantile plot for short sleep duration (left) and long sleep duration (right) in European MVP participants.

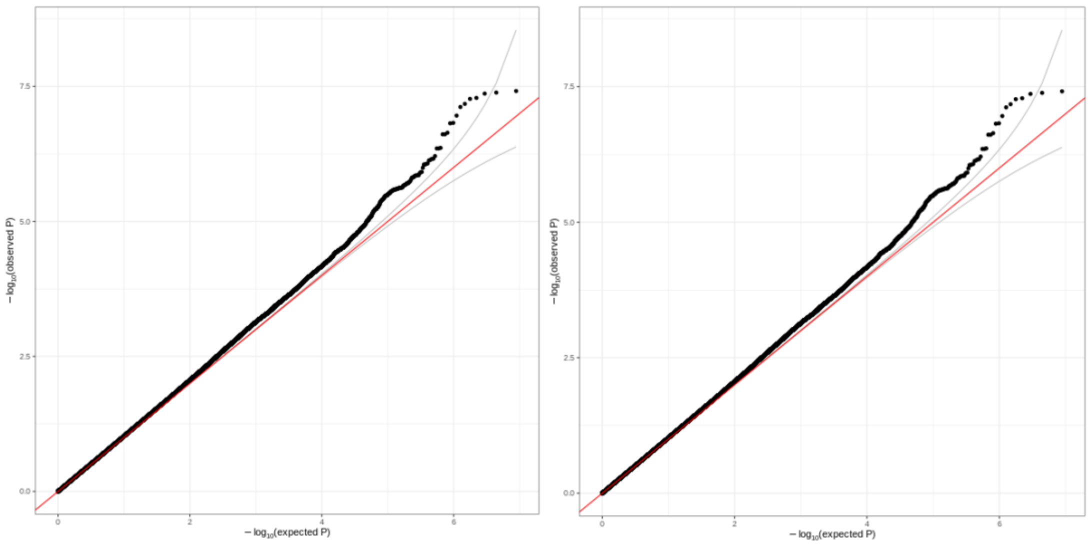

African-ancestry population

Supplementary Figure 11. Mirrored Manhattan plot showing genetic associations with short sleep duration (top) and long sleep duration (bottom) in African MVP participants. Loci reaching a nominal threshold of $1x10^{-5}$ highlighted in green, and the red line indicating genome-wide significance threshold of $5x10^{-8}$.

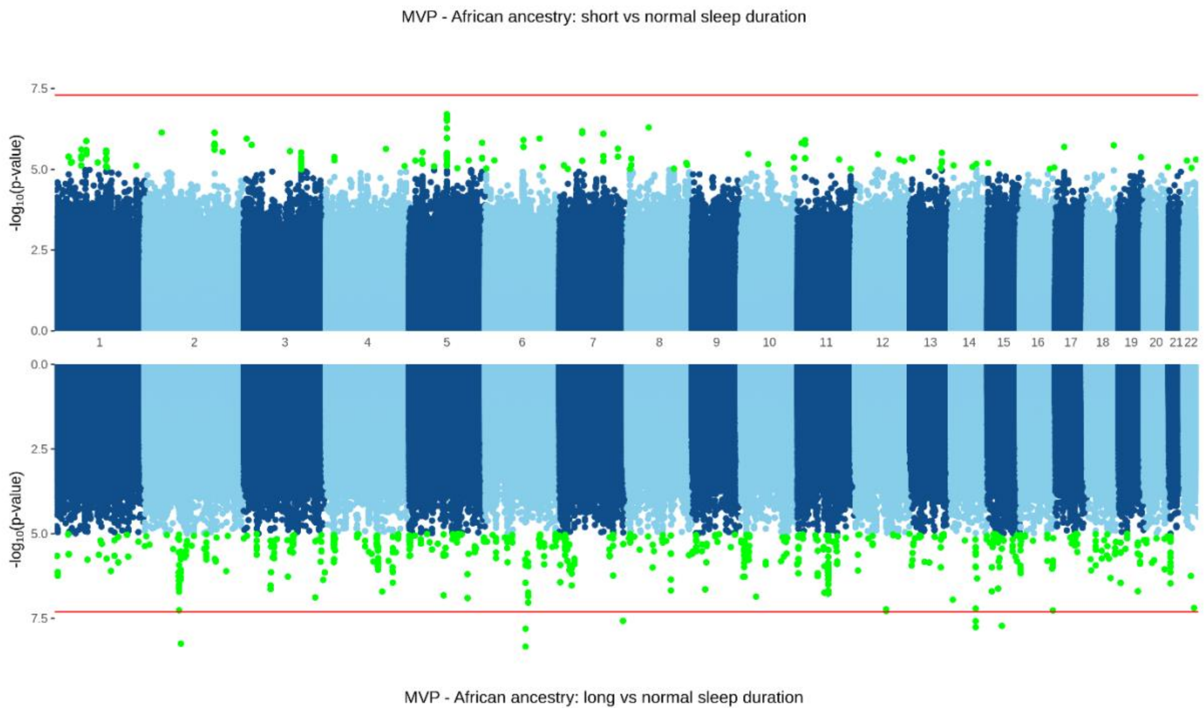

Supplementary Figure 12. Quantile-quantile plot for short sleep duration (left) and long sleep duration (right) in African MVP participants.

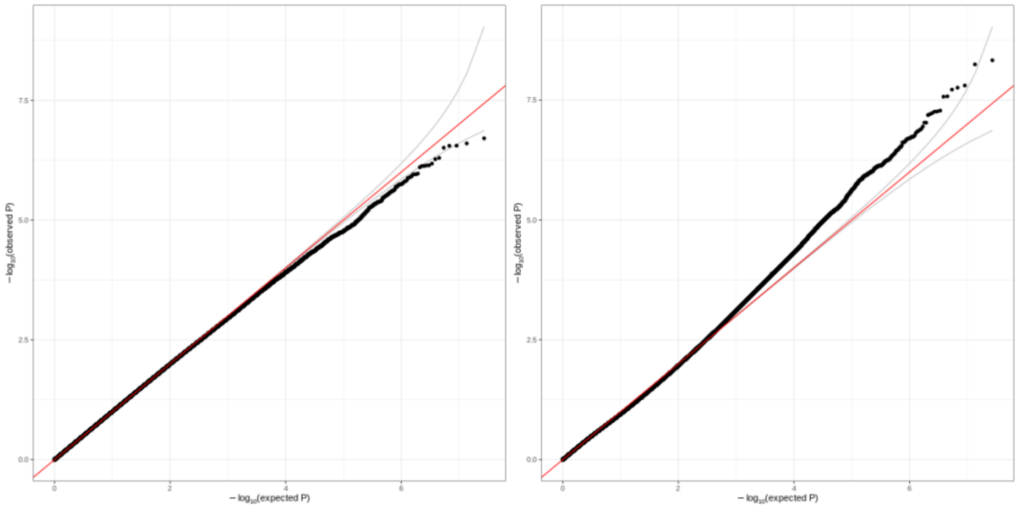

East Asian-ancestry population

Supplementary Figure 13. Mirrored Manhattan plot showing genetic associations with short sleep duration (top) and long sleep duration (bottom) in East Asian MVP participants. Loci reaching a nominal threshold of $1x10^{-5}$ highlighted in green.

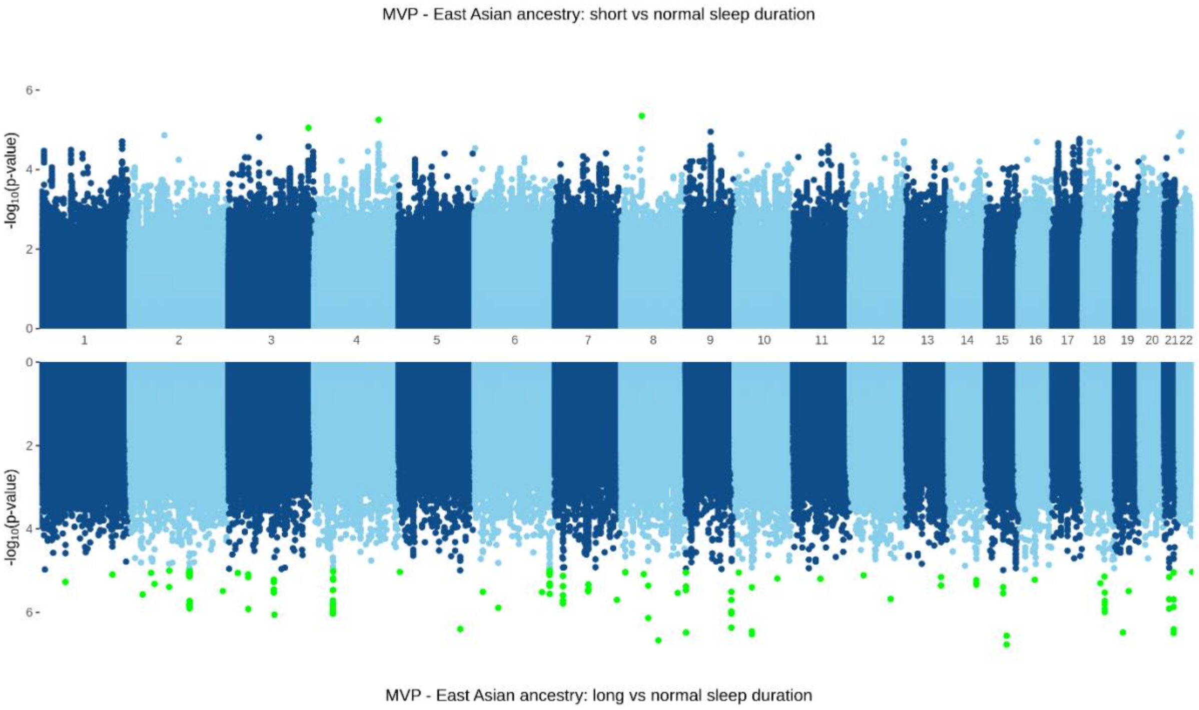

Supplementary Figure 14. Quantile-quantile plot for short sleep duration (left) and long sleep duration (right) in East Asian MVP participants.

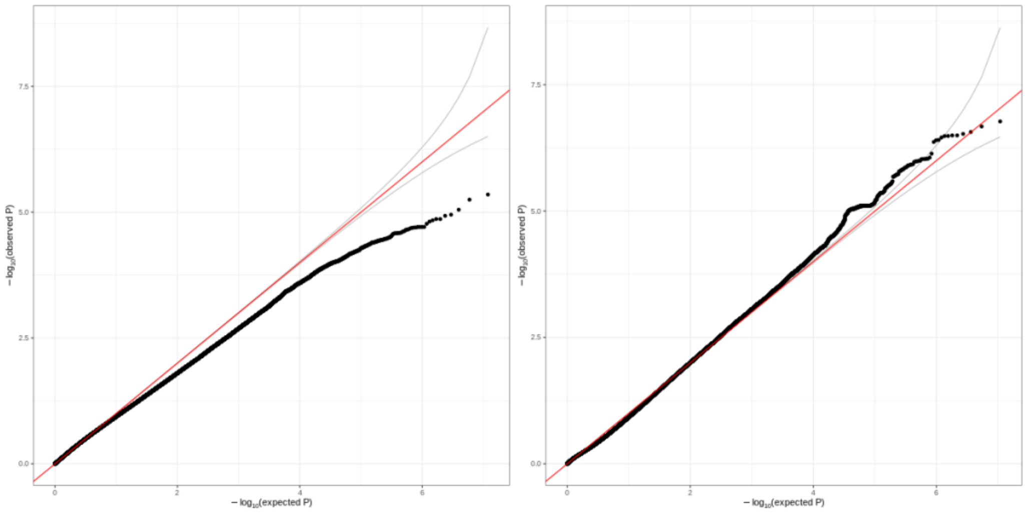

Admixed American population

Supplementary Figure 15. Mirrored Manhattan plot showing genetic associations with short sleep duration (top) and long sleep duration (bottom) in the East admixed-American MVP participants. Loci reaching a nominal threshold of $1x10^{-5}$ highlighted in green, and the red line indicating genome-wide significance threshold of $5x10^{-8}$.

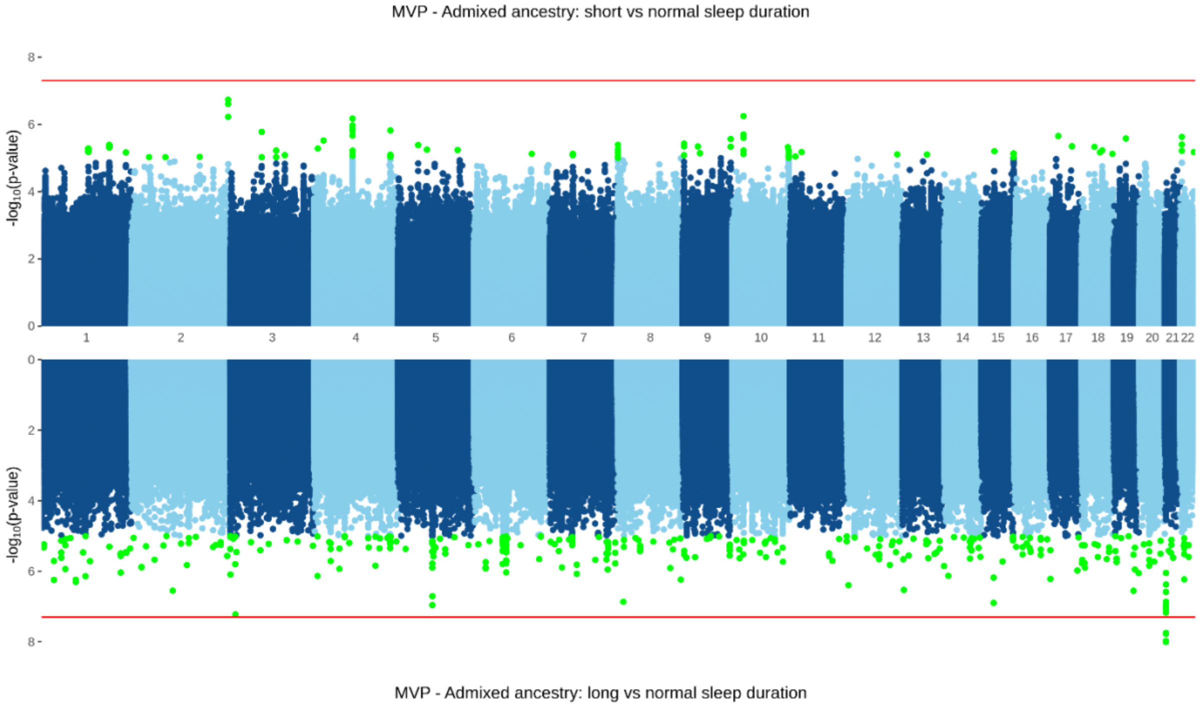

Supplementary Figure 16. Quantile-quantile plot for short sleep duration (left) and long sleep duration (right) in admixed-American MVP participants.

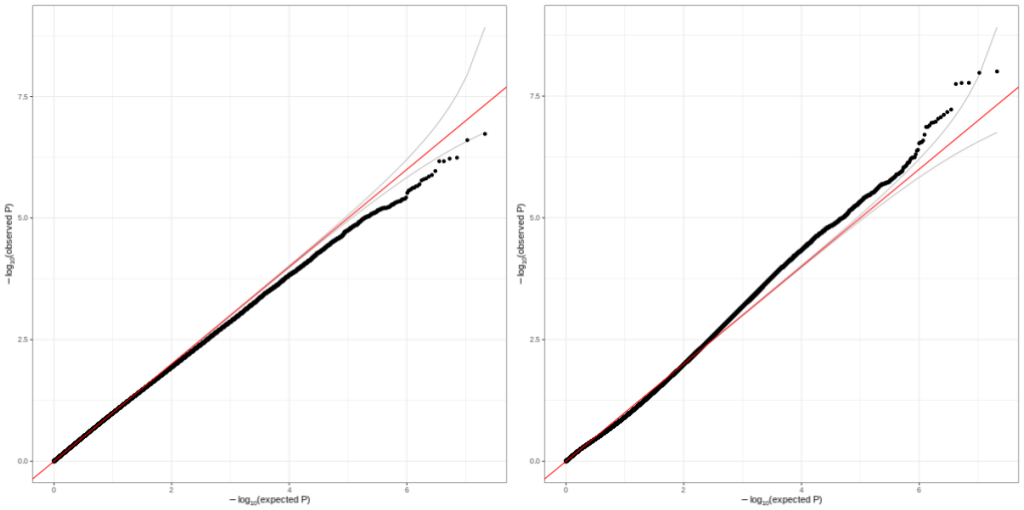

Ancestry-specific meta-analyses

European-ancestry population

Supplementary Figure 17 Mirrored Manhattan plot showing results of a meta-analysis in EUR groups in the UK Biobank and MVP cohorts. TOP: short (<6 hours, n=47,180) versus normal (7-8 hours, n=384,594), with 46 independent genetic-risk loci reaching genome-wide significance highlighted in green. BOTTOM: long (>9 hours, n=15,995) versus normal with one genome wide significant locus. All variants reaching a suggestive threshold of $1x10^{-5}$ are highlighted in green.

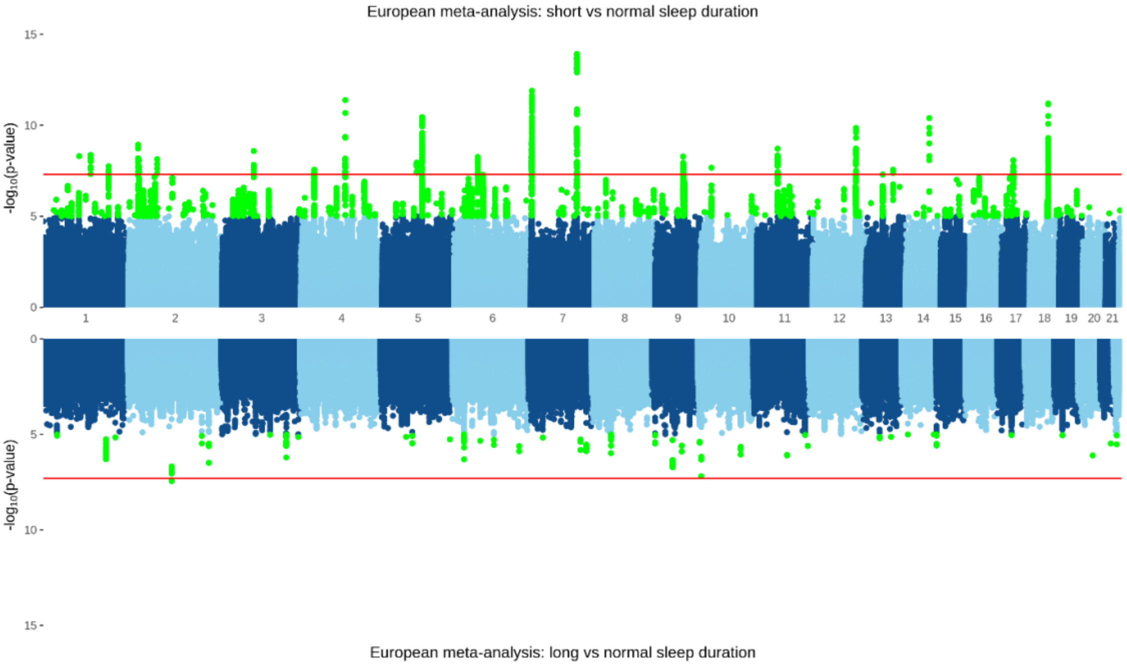

Supplementary Figure 18 Gene-based test for short (top) and long (bottom) sleep duration in EUR from UK Biobank and MVP cohorts. Bonferroni-adjusted significance threshold $P<2.7x10^{-6}$

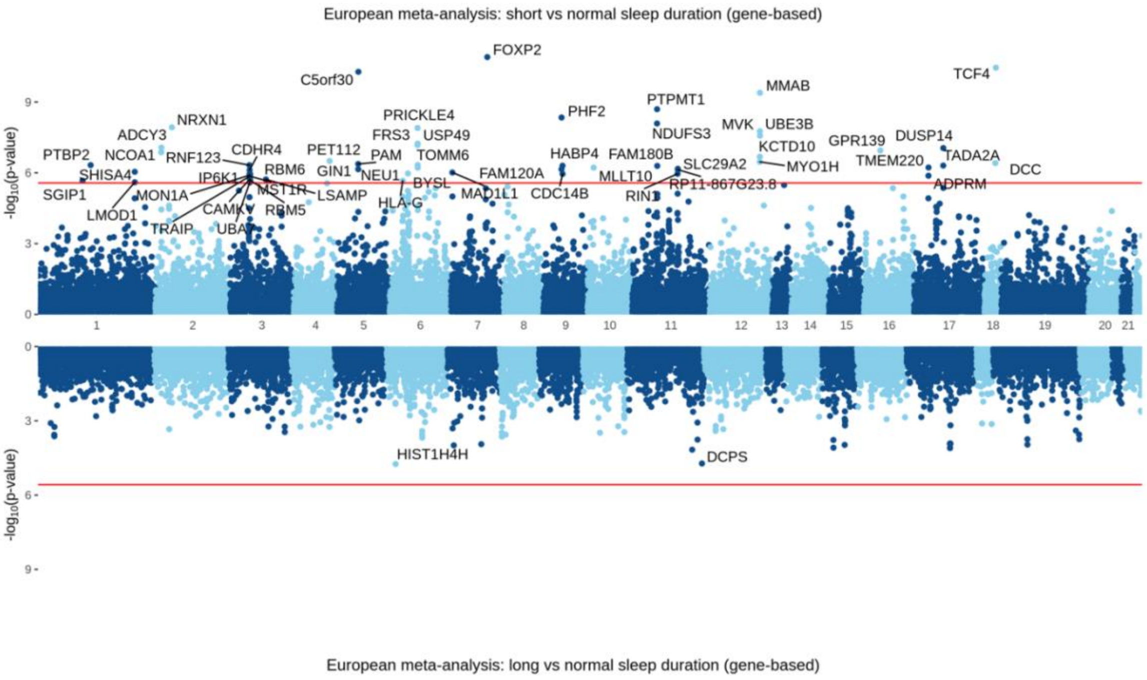

Figure 19 Regional plots showing novel loci for EUR short sleep analysis. Images generated by FUMA.

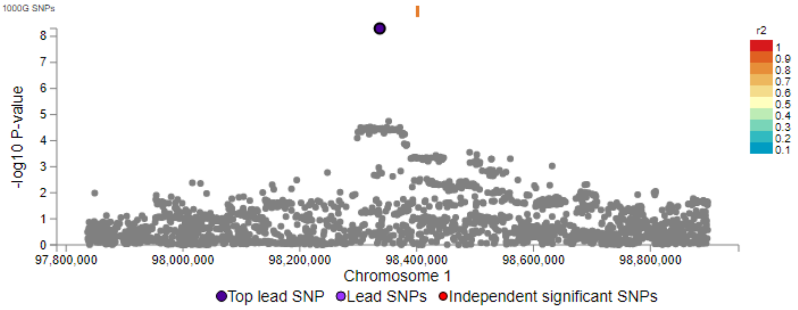

Ind. Sig. SNP	rs11165922
Chrom	1
BP	98336823
P-value	4.976e-09
SNPs within LD	2
GWAS SNPs within LD	1

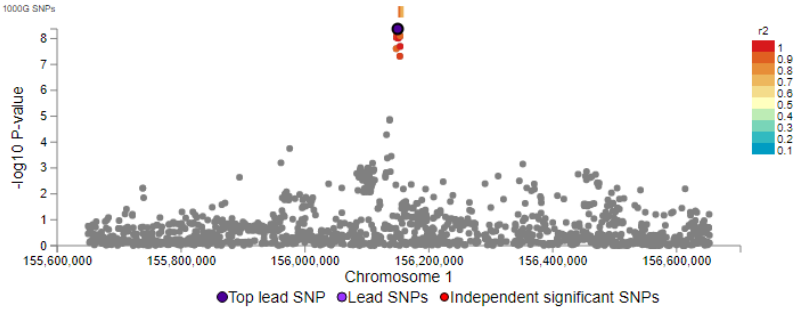

Ind. Sig. SNP	rs201640077
Chrom	1
BP	156149659
P-value	4.242e-09
SNPs within LD	17
GWAS SNPs within LD	14

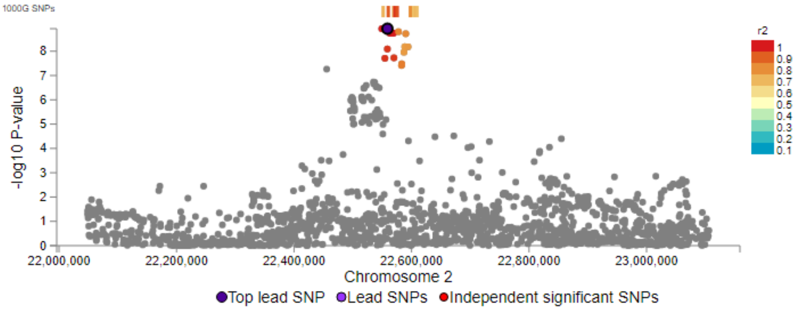

Ind. Sig. SNP	rs57537843
Chrom	2
BP	22558973
P-value	1.184e-09
SNPs within LD	33
GWAS SNPs within LD	17

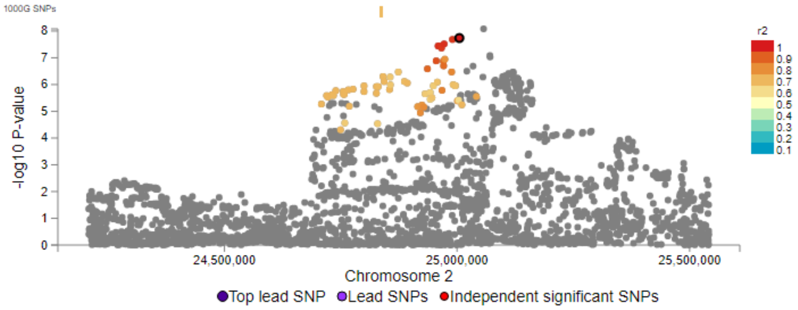

Ind. Sig. SNP	rs62144584
Chrom	2
BP	25004937
P-value	1.83e-08
SNPs within LD	60
GWAS SNPs within LD	59

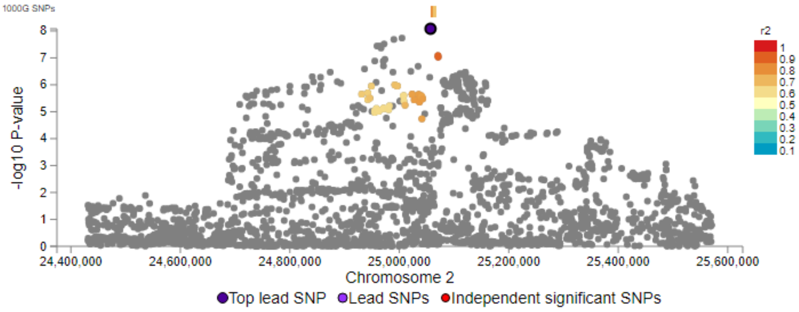

Ind. Sig. SNP	rs11689042
Chrom	2
BP	25057054
P-value	8.28e-09
SNPs within LD	39
GWAS SNPs within LD	36

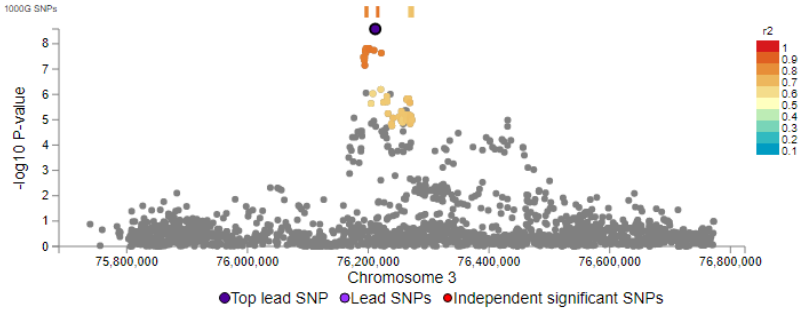

Ind. Sig. SNP	rs11128505
Chrom	3
BP	76211681
P-value	2.614e-09
SNPs within LD	54
GWAS SNPs within LD	48

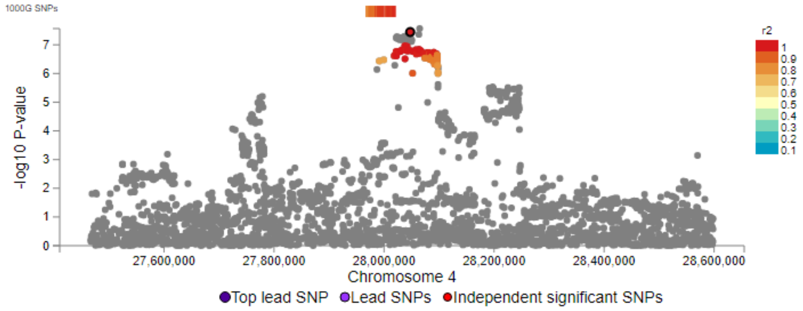

Ind. Sig. SNP	rs796217750
Chrom	4
BP	28047538
P-value	3.594e-08
SNPs within LD	164
GWAS SNPs within LD	119

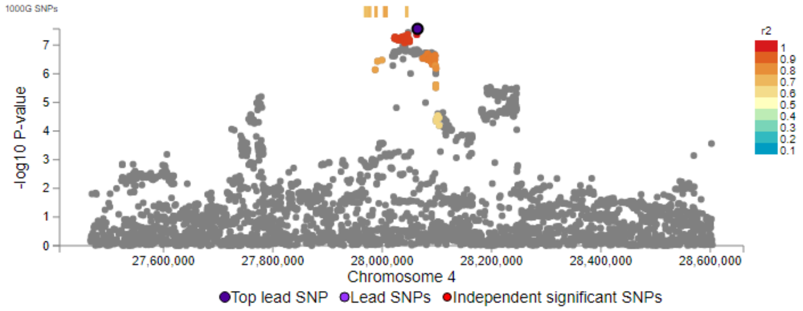

Ind. Sig. SNP	rs6852351
Chrom	4
BP	28064697
P-value	2.719e-08
SNPs within LD	158
GWAS SNPs within LD	140

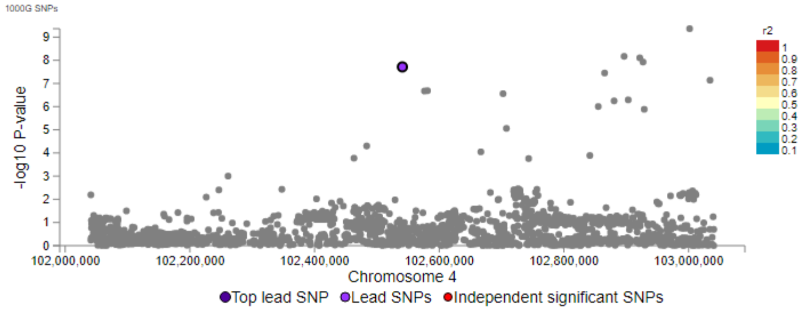

Ind. Sig. SNP	rs71614699
Chrom	4
BP	102540919
P-value	1.932e-08
SNPs within LD	1
GWAS SNPs within LD	1

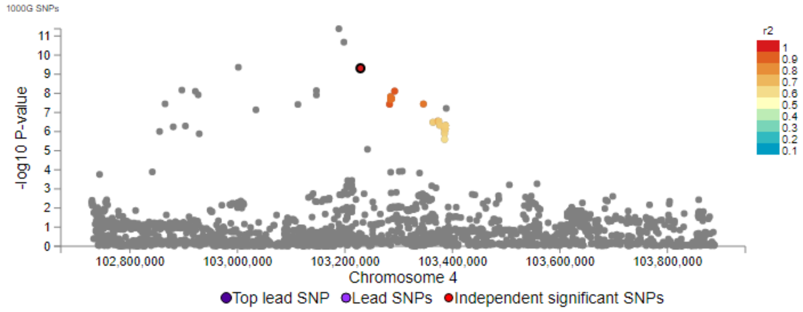

Ind. Sig. SNP	rs13105581
Chrom	4
BP	103228830
P-value	4.867e-10
SNPs within LD	19
GWAS SNPs within LD	19

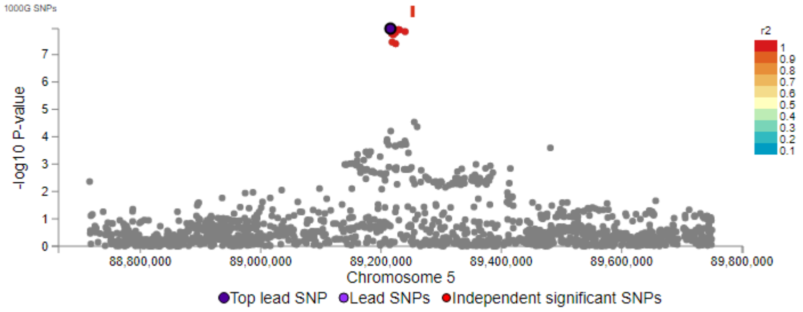

Ind. Sig. SNP	rs10079421
Chrom	5
BP	89215661
P-value	1.124e-08
SNPs within LD	13
GWAS SNPs within LD	10

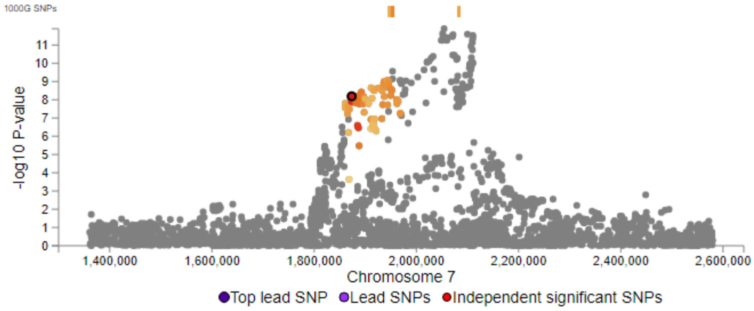

Ind. Sig. SNP	rs10268294
Chrom	7
BP	1873406
P-value	6.478e-09
SNPs within LD	90
GWAS SNPs within LD	85

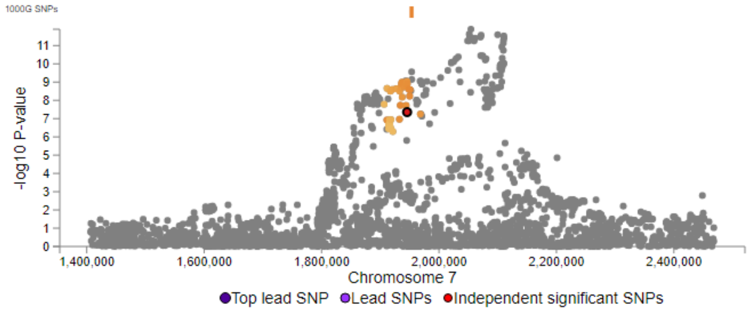

Ind. Sig. SNP	7:1945685_CAAA_C
Chrom	7
BP	1945685
P-value	4.399e-08
SNPs within LD	41
GWAS SNPs within LD	39

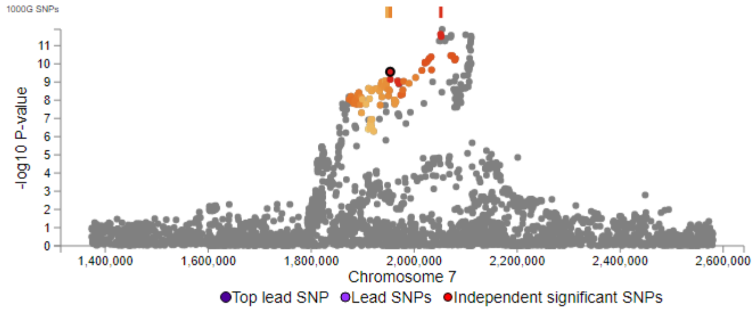

Ind. Sig. SNP	rs62442903
Chrom	7
BP	1953416
P-value	2.734e-10
SNPs within LD	90
GWAS SNPs within LD	86

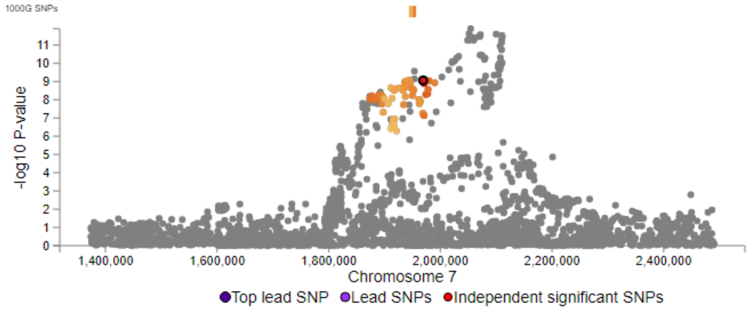

Ind. Sig. SNP	rs62442910
Chrom	7
BP	1969133
P-value	8.757e-10
SNPs within LD	69
GWAS SNPs within LD	66

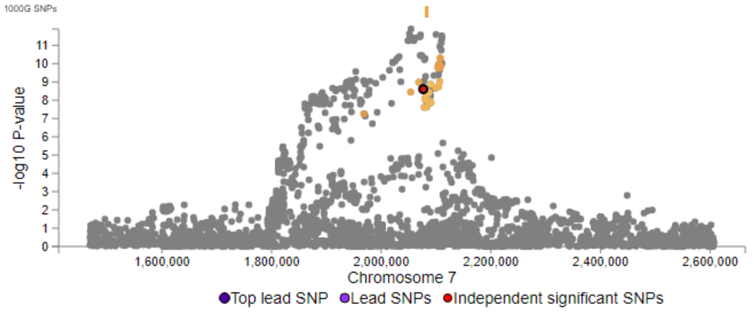

Ind. Sig. SNP	rs6948912
Chrom	7
BP	2076701
P-value	2.482e-09
SNPs within LD	32
GWAS SNPs within LD	30

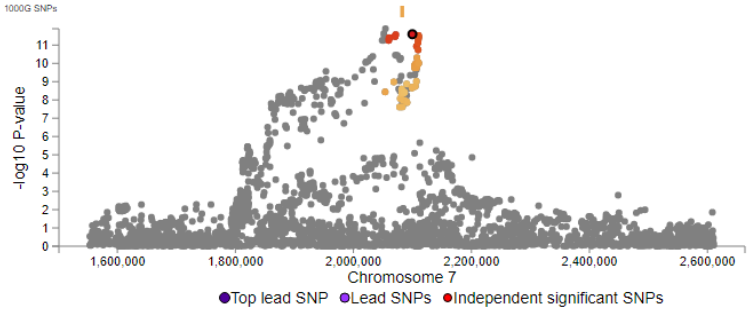

Ind. Sig. SNP	rs111595851
Chrom	7
BP	2099924
P-value	2.539e-12
SNPs within LD	46
GWAS SNPs within LD	44

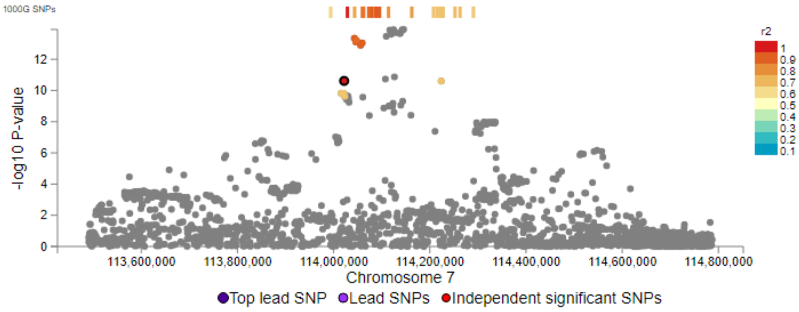

Ind. Sig. SNP	rs66571810
Chrom	7
BP	114021870
P-value	2.433e-11
SNPs within LD	33
GWAS SNPs within LD	13

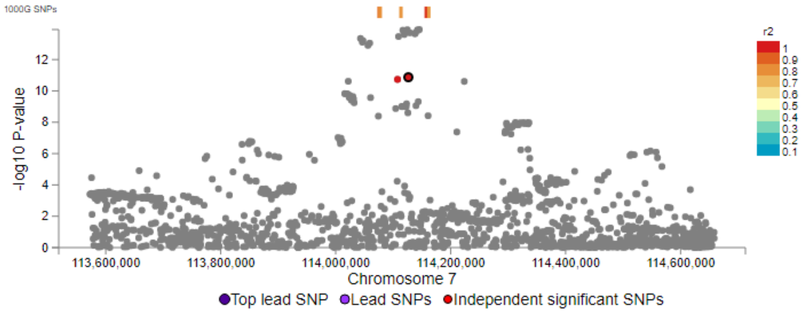

Ind. Sig. SNP	rs2079070
Chrom	7
BP	114126432
P-value	1.347e-11
SNPs within LD	7
GWAS SNPs within LD	2

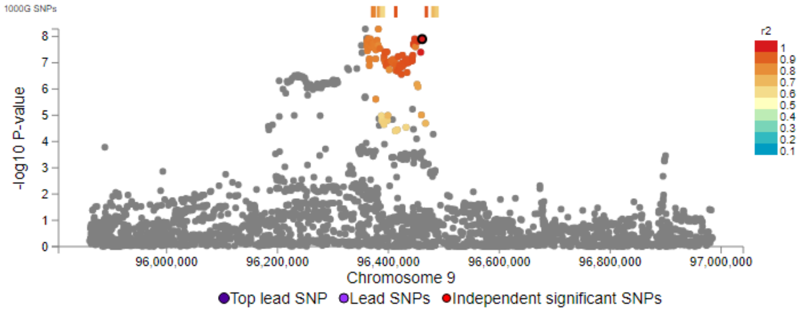

Ind. Sig. SNP	rs765110298
Chrom	9
BP	96460883
P-value	1.258e-08
SNPs within LD	128
GWAS SNPs within LD	111

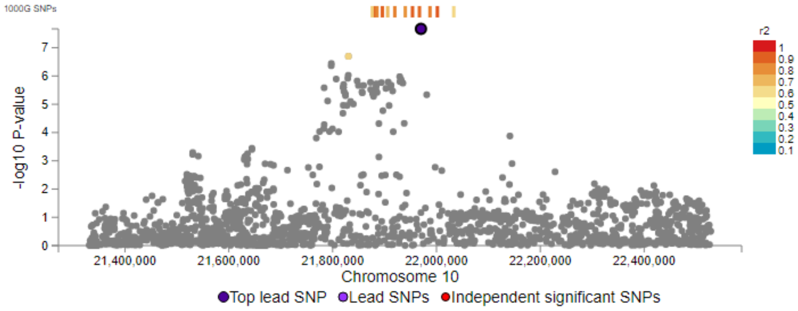

Ind. Sig. SNP	rs1243197
Chrom	10
BP	21970077
P-value	2.152e-08
SNPs within LD	17
GWAS SNPs within LD	2

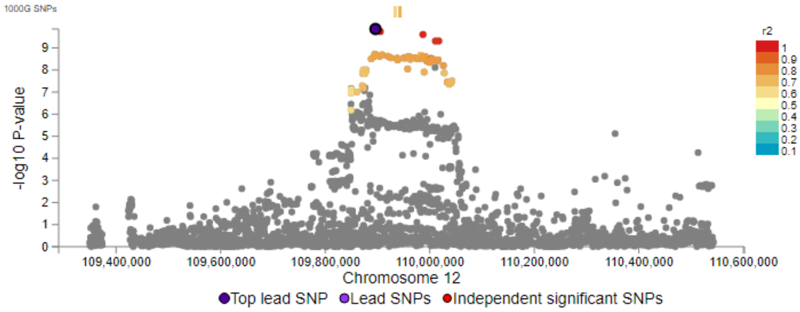

Ind. Sig. SNP	rs7313797
Chrom	12
BP	109896165
P-value	1.394e-10
SNPs within LD	79
GWAS SNPs within LD	77

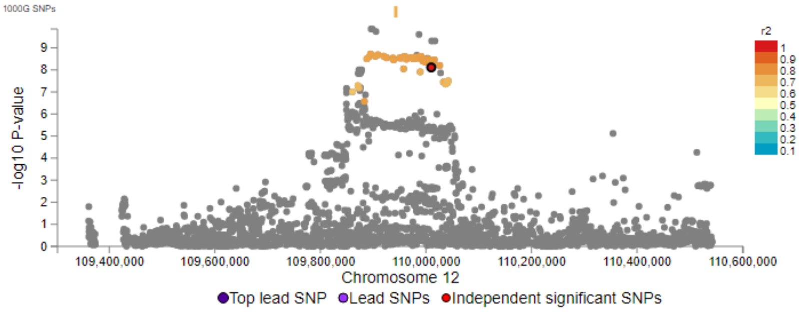

Ind. Sig. SNP	rs2111216
Chrom	12
BP	110010151
P-value	7.725e-09
SNPs within LD	58
GWAS SNPs within LD	57

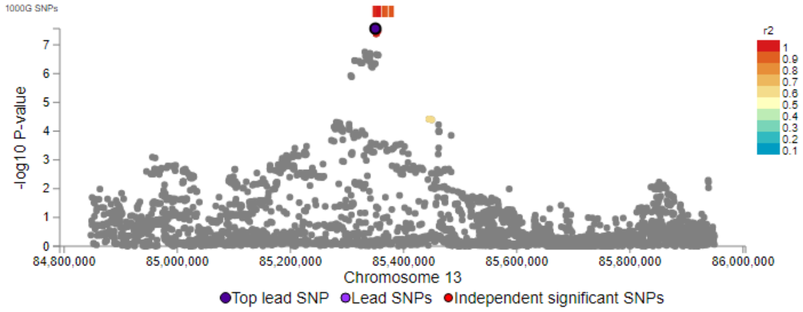

Ind. Sig. SNP	rs72629492
Chrom	13
BP	85350099
P-value	2.737e-08
SNPs within LD	29
GWAS SNPs within LD	12

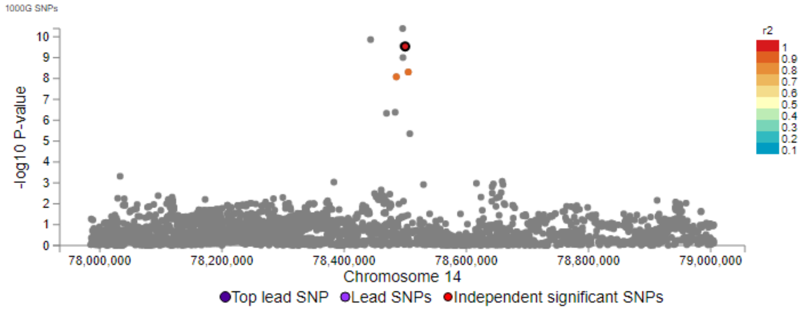

Ind. Sig. SNP	rs76251410
Chrom	14
BP	78499897
P-value	2.922e-10
SNPs within LD	3
GWAS SNPs within LD	3

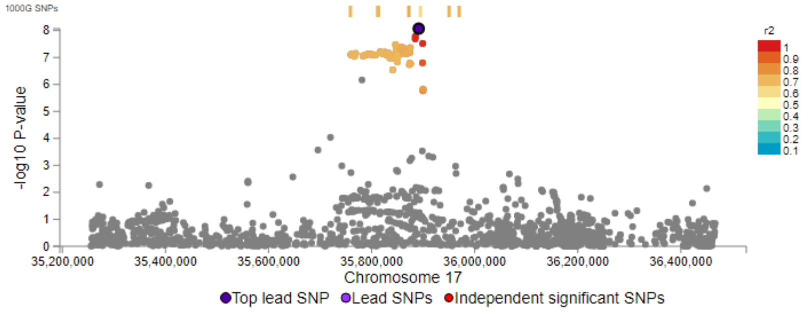

Ind. Sig. SNP	rs3110628
Chrom	17
BP	35891384
P-value	8.524e-09
SNPs within LD	73
GWAS SNPs within LD	65

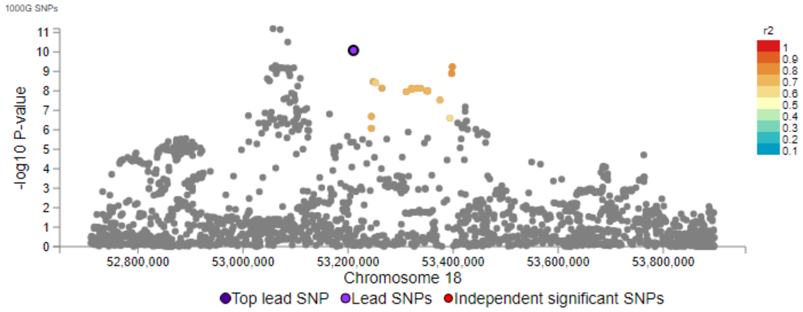

Ind. Sig. SNP	rs613872
Chrom	18
BP	53210302
P-value	8.35e-11
SNPs within LD	17
GWAS SNPs within LD	17

Figure 20 Regional plot for rs62158206, a genome-wide significant association with long sleep in EUR-only meta-analysis of UKBB and MVP. Image generated using FUMA.

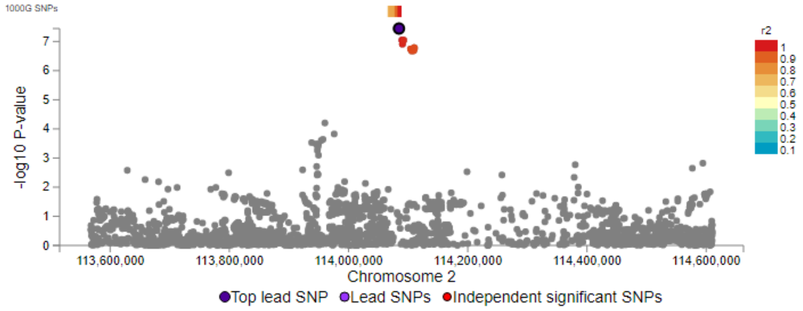

Ind. Sig. SNP	rs62158206
Chrom	2
BP	114084596
P-value	3.589e-08
SNPs within LD	24
GWAS SNPs within LD	10

African-ancestry population

Supplementary Figure 21 Mirrored Manhattan plot showing results of a meta-analysis in AFR in the UK Biobank and MVP cohorts. TOP: short (<6 hours, n=11,352) versus normal (7-8 hours, n=15,305), with no genome-wide significance loci. BOTTOM: long (>9 hours, n=1,128) versus normal with one genome-wide significant locus. All variants reaching a suggestive threshold of $1x10^{-5}$ are highlighted in green.

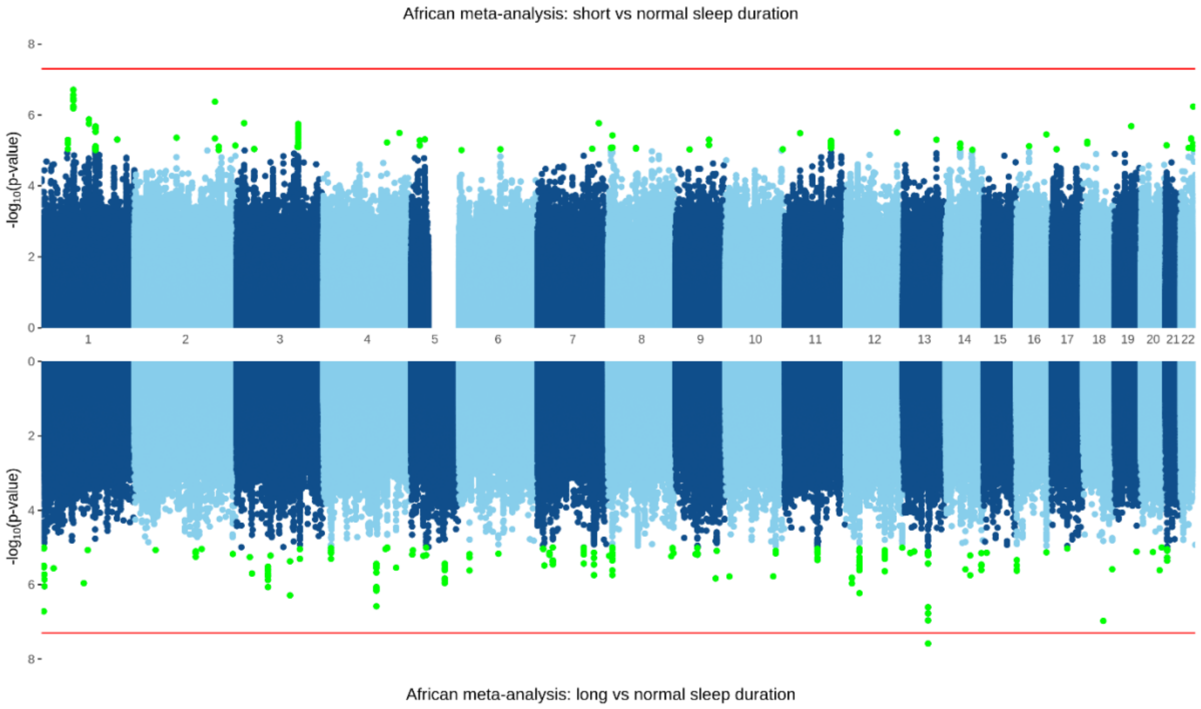

Supplementary Figure 22 Gene-based test for short (top) and long (bottom) sleep duration in African participants from UK Biobank and MVP cohorts, with top associations labelled.

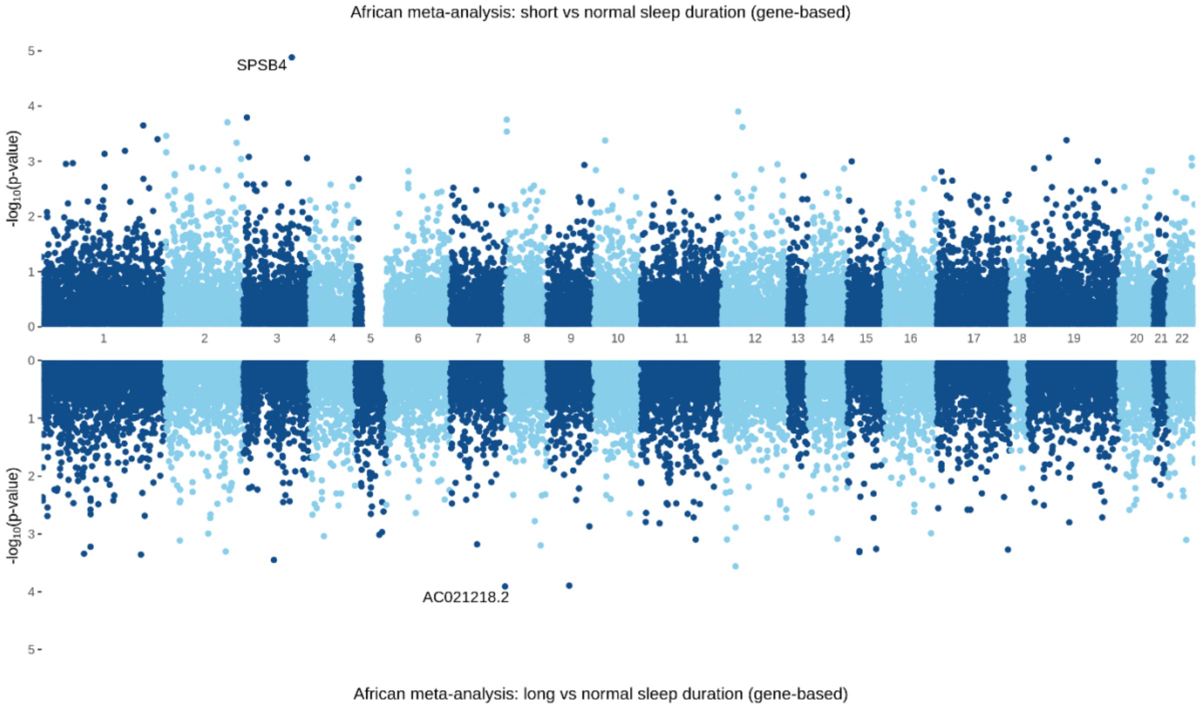

Figure 23 Regional plot for rs1412139, the strongest association for short sleep in an AFR meta-analysis of UKBB and MVP. Image generated using FUMA.

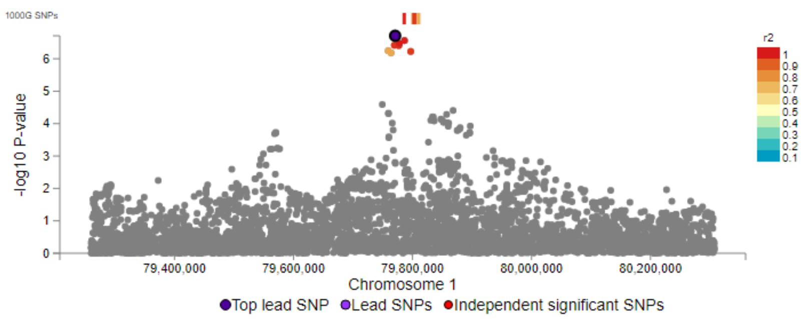

Ind. Sig. SNP	rs1412139
Chrom	1
BP	79770776
P-value	1.949e-07
SNPs within LD	13
GWAS SNPs within LD	8

Figure 24 Regional plot for rs148926698, genome-wide significant association for long sleep in an AFR meta-analysis of UKBB and MVP. Image generated using FUMA.

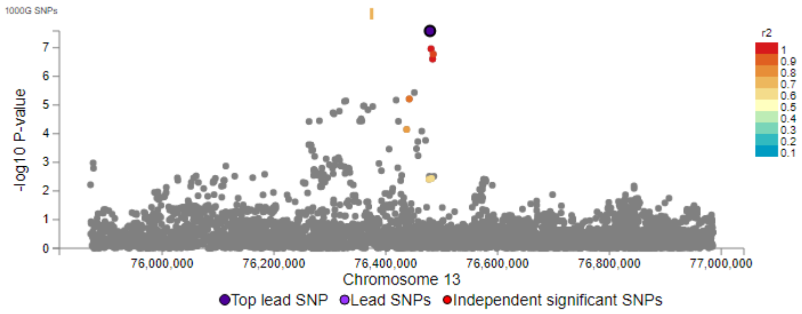

Ind. Sig. SNP	rs148926968
Chrom	13
BP	76479075
P-value	2.601e-08
SNPs within LD	9
GWAS SNPs within LD	8

2. Case-case analysis

We conducted a case-case analysis, comparing the short versus long sleep duration in the EUR sample of UK Biobank and MVP. This analysis revealed two independent genome-wide significant loci on chromosome 2. Firstly, rs62158206, which was genome-wide significant in the EUR meta-analyses of both short and long sleep duration (with opposite directions of effect). Secondly, rs4618068, which was a sub-threshold association in the EUR meta-analyses of both short ($p=5.67x10^{-7}$) and long ($p=2.05x10^{-7}$), again with contrasting effect directions. See supplementary table 27 and

Supplementary Figure 27 for further details

Figure 25 Manhattan plot showing results of a short (<6 hours, N=47,180) versus long (>9 hours, N=15,995) meta-analysis in EUR in the UK Biobank and MVP cohorts.

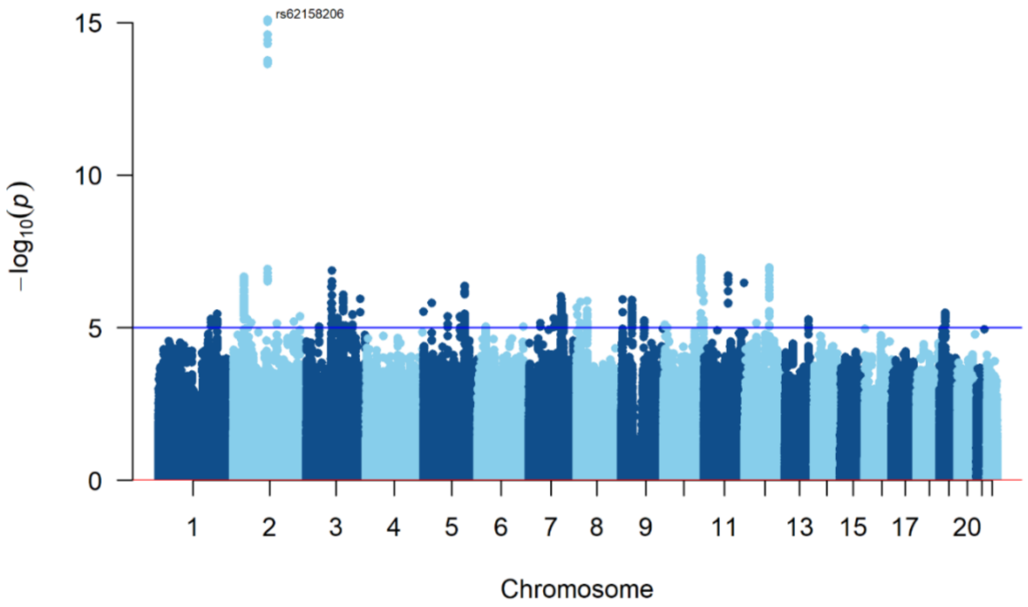

Figure 26 Quantile-quantile plot for short versus long sleep duration in EUR participants from the UKBB and MVP cohorts.

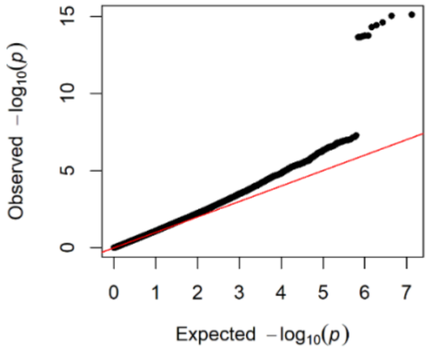

Supplementary Figure 27 Forest plots showing top associations in the short vs normal, long vs normal and short vs long analyses (EUR-only meta-analyses). Forest plots show the beta-coefficient and standard error for each SNP.

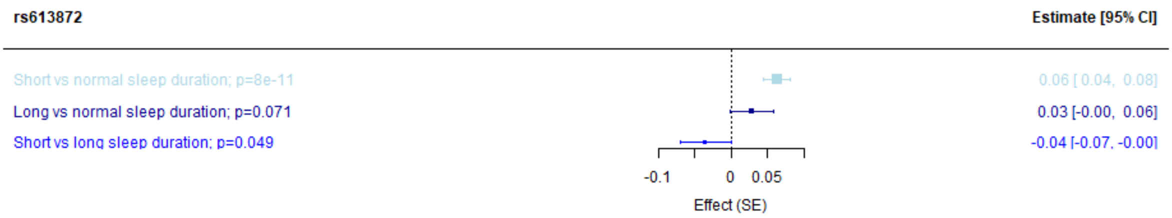

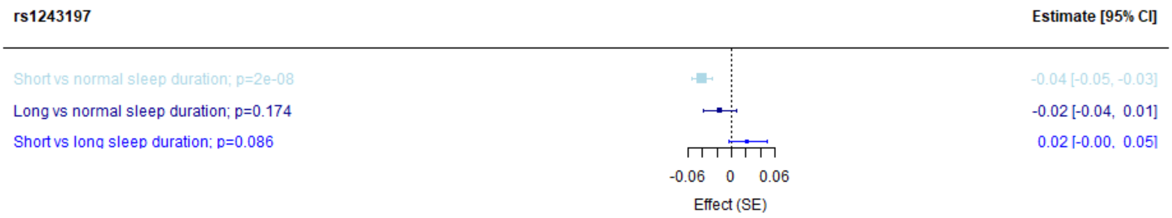

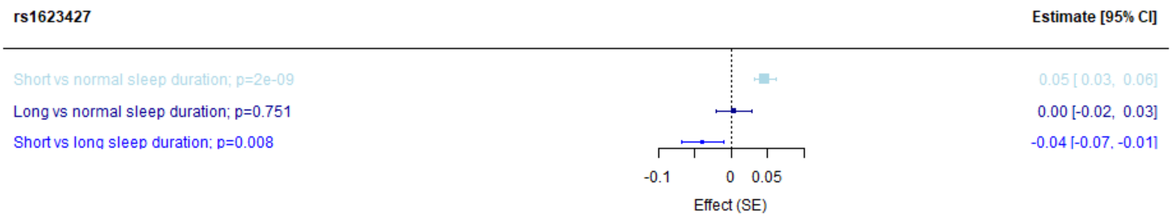

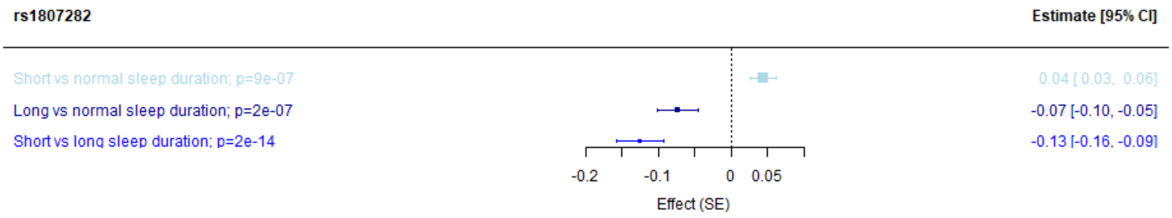

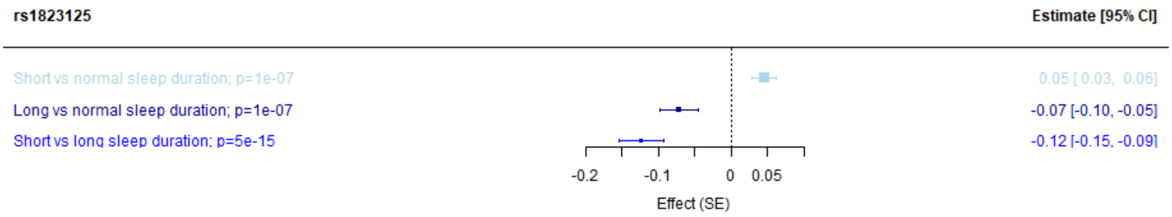

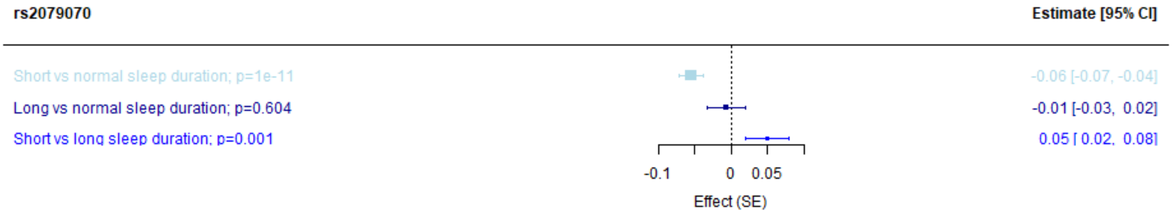
rs2079070
Estimate [95% CI]
Short vs normal sleep duration; p=1e-11
Long vs normal sleep duration; p=0.604
Short vs long sleep duration; p=0.001
-0.06 [-0.07, -0.04]
-0.01 [-0.03, 0.02]
0.05 [0.02, 0.08]
-0.1
0
0.05
Effect (SE)

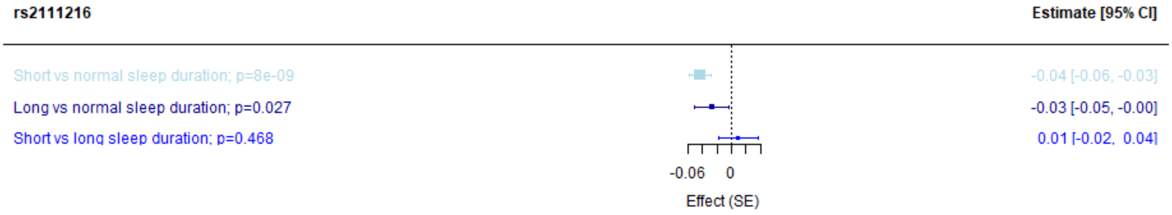
rs2111216
Estimate [95% CI]
Short vs normal sleep duration; p=8e-09
Long vs normal sleep duration; p=0.027
Short vs long sleep duration; p=0.468
-0.04 [-0.06, -0.03]
-0.03 [-0.05, -0.00]
0.01 [-0.02, 0.04]
-0.06
0
Effect (SE)

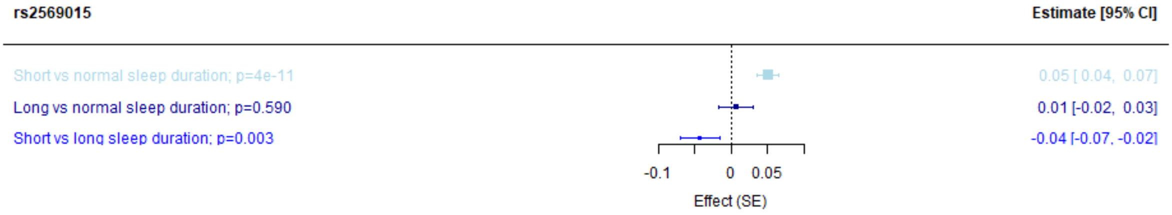
rs2569015
Estimate [95% CI]
Short vs normal sleep duration; p=4e-11
Long vs normal sleep duration; p=0.590
Short vs long sleep duration; p=0.003
0.05 [0.04, 0.07]
0.01 [-0.02, 0.03]
-0.04 [-0.07, -0.02]
-0.1
0
0.05
Effect (SE)

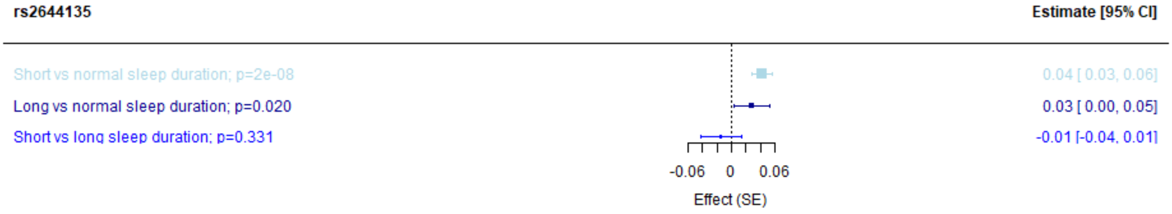
rs2644135
Estimate [95% CI]
Short vs normal sleep duration; p=2e-08
Long vs normal sleep duration; p=0.020
Short vs long sleep duration; p=0.331
0.04 [0.03, 0.06]
0.03 [0.00, 0.05]
-0.01 [-0.04, 0.01]
-0.06
0
0.06
Effect (SE)

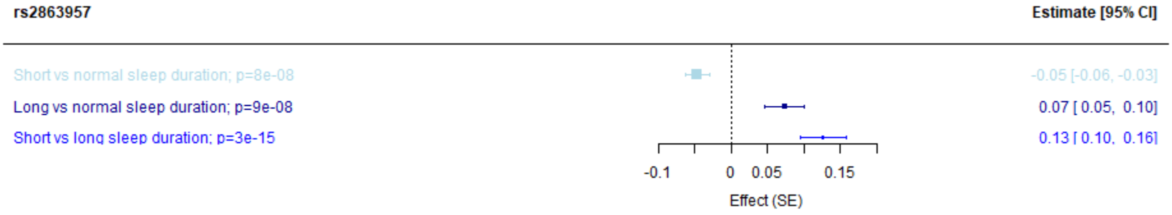
rs2863957
Estimate [95% CI]
Short vs normal sleep duration; p=8e-08
Long vs normal sleep duration; p=9e-08
Short vs long sleep duration; p=3e-15
-0.05 [-0.06, -0.03]
0.07 [0.05, 0.10]
0.13 [0.10, 0.16]
-0.1
0
0.05
0.15
Effect (SE)

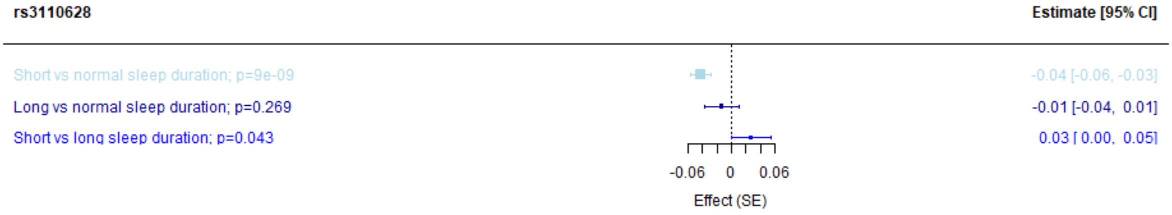
rs3110628
Estimate [95% CI]
Short vs normal sleep duration; p=9e-09
Long vs normal sleep duration; p=0.269
Short vs long sleep duration; p=0.043
-0.04 [-0.06, -0.03]
-0.01 [-0.04, 0.01]
0.03 [0.00, 0.05]
-0.06
0
0.06
Effect (SE)

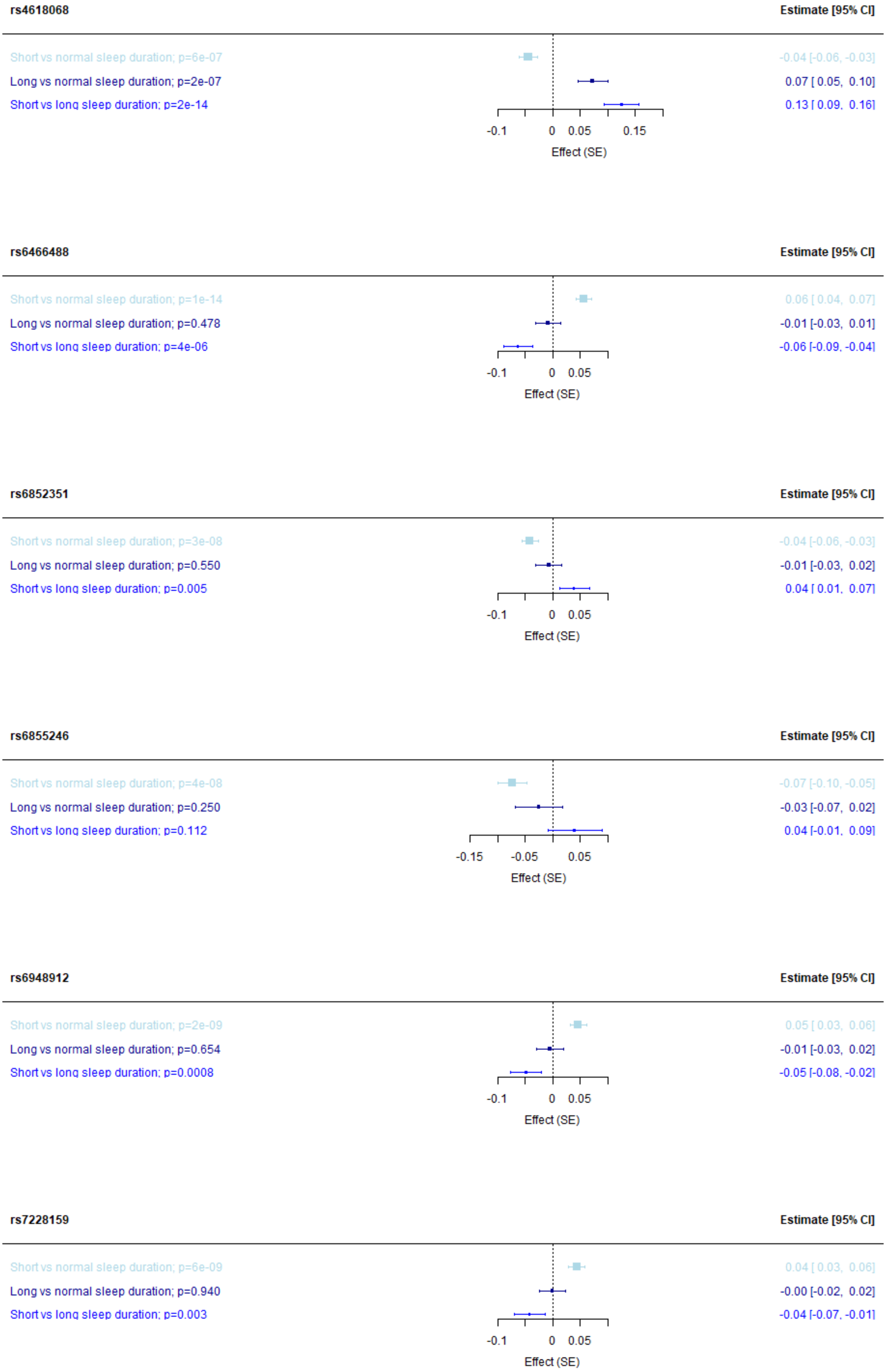
rs4618068
Estimate [95% CI]
Short vs normal sleep duration; p=6e-07
Long vs normal sleep duration; p=2e-07
Short vs long sleep duration; p=2e-14
-0.04 [-0.06, -0.03]
0.07 [0.05, 0.10]
0.13 [0.09, 0.16]
-0.1
0
0.05
0.15
Effect (SE)
rs6466488
Estimate [95% CI]
Short vs normal sleep duration; p=1e-14
Long vs normal sleep duration; p=0.478
Short vs long sleep duration; p=4e-06
0.06 [0.04, 0.07]
-0.01 [-0.03, 0.01]
-0.06 [-0.09, -0.04]
-0.1
0
0.05
Effect (SE)
rs6852351
Estimate [95% CI]
Short vs normal sleep duration; p=3e-08
Long vs normal sleep duration; p=0.550
Short vs long sleep duration; p=0.005
-0.04 [-0.06, -0.03]
-0.01 [-0.03, 0.02]
0.04 [0.01, 0.07]
-0.1
0
0.05
Effect (SE)
rs6855246
Estimate [95% CI]
Short vs normal sleep duration; p=4e-08
Long vs normal sleep duration; p=0.250
Short vs long sleep duration; p=0.112
-0.07 [-0.10, -0.05]
-0.03 [-0.07, 0.02]
0.04 [-0.01, 0.09]
-0.15
-0.05
0.05
Effect (SE)
rs6948912
Estimate [95% CI]
Short vs normal sleep duration; p=2e-09
Long vs normal sleep duration; p=0.654
Short vs long sleep duration; p=0.0008
0.05 [0.03, 0.06]
-0.01 [-0.03, 0.02]
-0.05 [-0.08, -0.02]
-0.1
0
0.05
Effect (SE)
rs7228159
Estimate [95% CI]
Short vs normal sleep duration; p=6e-09
Long vs normal sleep duration; p=0.940
Short vs long sleep duration; p=0.003
0.04 [0.03, 0.06]
-0.00 [-0.02, 0.02]
-0.04 [-0.07, -0.01]
-0.1
0
0.05
Effect (SE)

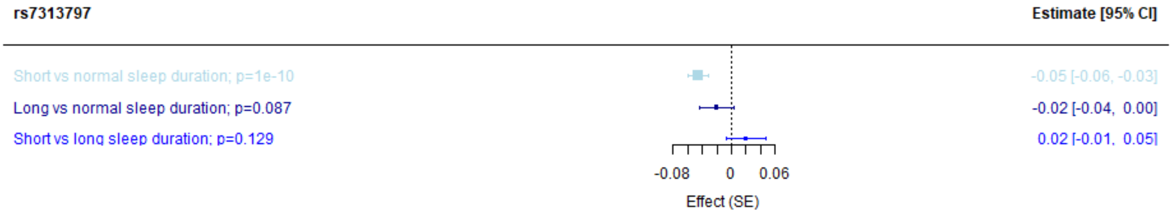
rs7313797
Estimate [95% CI]
Short vs normal sleep duration; p=1e-10
Long vs normal sleep duration; p=0.087
Short vs long sleep duration; p=0.129
-0.05 [-0.06, -0.03]
-0.02 [-0.04, 0.00]
0.02 [-0.01, 0.05]
-0.08
0
0.06
Effect (SE)

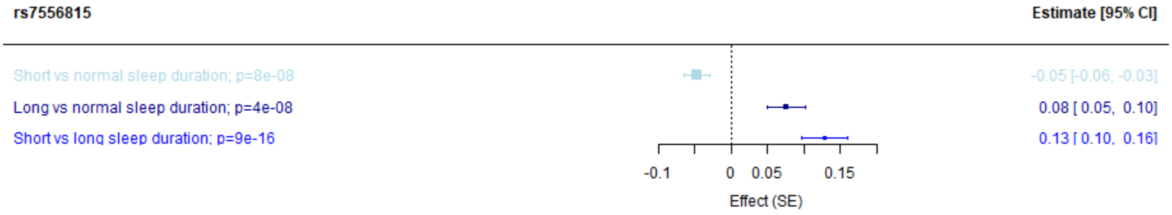
rs7556815
Estimate [95% CI]
Short vs normal sleep duration; p=8e-08
Long vs normal sleep duration; p=4e-08
Short vs long sleep duration; p=9e-16
-0.05 [-0.06, -0.03]
0.08 [0.05, 0.10]
0.13 [0.10, 0.16]
-0.1
0
0.05
0.15
Effect (SE)

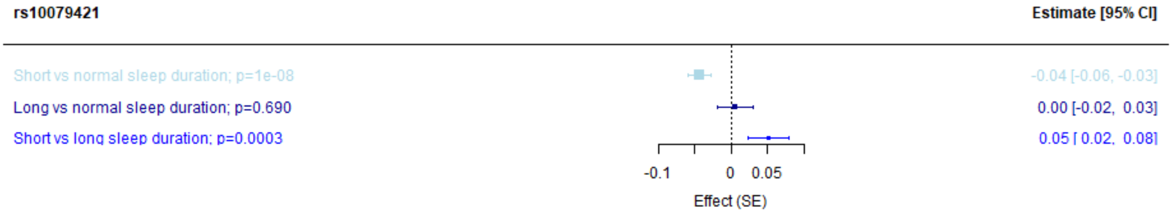
rs10079421
Estimate [95% CI]
Short vs normal sleep duration; p=1e-08
Long vs normal sleep duration; p=0.690
Short vs long sleep duration; p=0.0003
-0.04 [-0.06, -0.03]
0.00 [-0.02, 0.03]
0.05 [0.02, 0.08]
-0.1
0
0.05
Effect (SE)

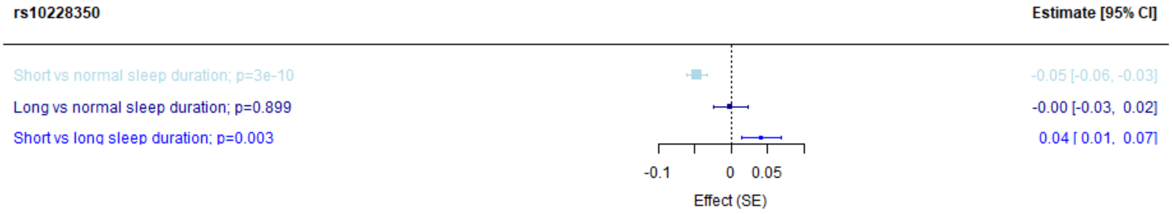
rs10228350
Estimate [95% CI]
Short vs normal sleep duration; p=3e-10
Long vs normal sleep duration; p=0.899
Short vs long sleep duration; p=0.003
-0.05 [-0.06, -0.03]
-0.00 [-0.03, 0.02]
0.04 [0.01, 0.07]
-0.1
0
0.05
Effect (SE)

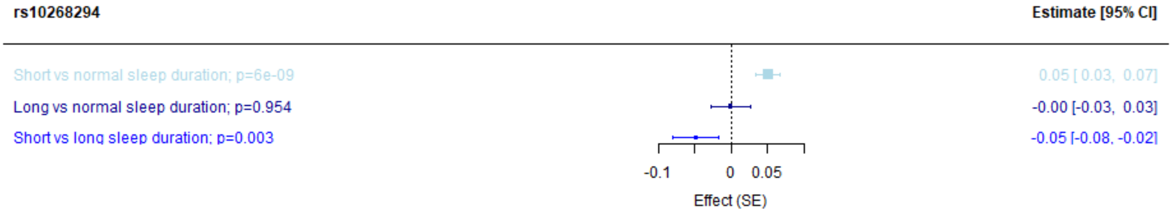
rs10268294
Estimate [95% CI]
Short vs normal sleep duration; p=6e-09
Long vs normal sleep duration; p=0.954
Short vs long sleep duration; p=0.003
0.05 [0.03, 0.07]
-0.00 [-0.03, 0.03]
-0.05 [-0.08, -0.02]
-0.1
0
0.05
Effect (SE)

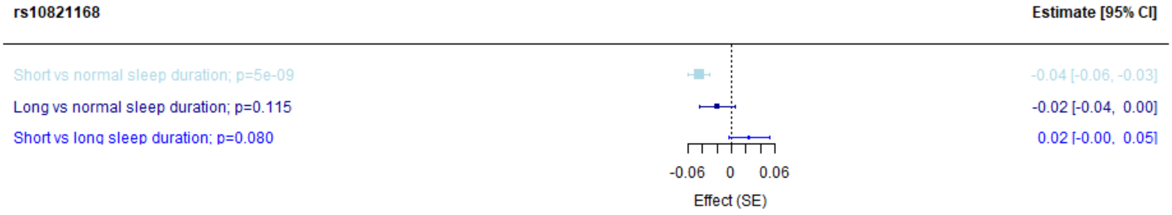
rs10821168
Estimate [95% CI]
Short vs normal sleep duration; p=5e-09
Long vs normal sleep duration; p=0.115
Short vs long sleep duration; p=0.080
-0.04 [-0.06, -0.03]
-0.02 [-0.04, 0.00]
0.02 [-0.00, 0.05]
-0.06
0
0.06
Effect (SE)

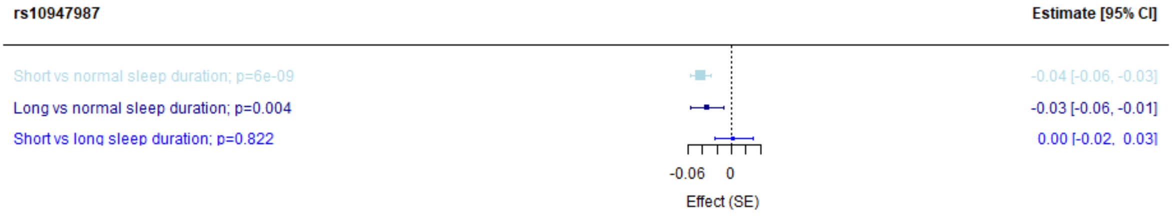
rs10947987
Estimate [95% CI]
Short vs normal sleep duration; p=6e-09
Long vs normal sleep duration; p=0.004
Short vs long sleep duration; p=0.822
-0.04 [-0.06, -0.03]
-0.03 [-0.06, -0.01]
0.00 [-0.02, 0.03]
-0.06
0
Effect (SE)

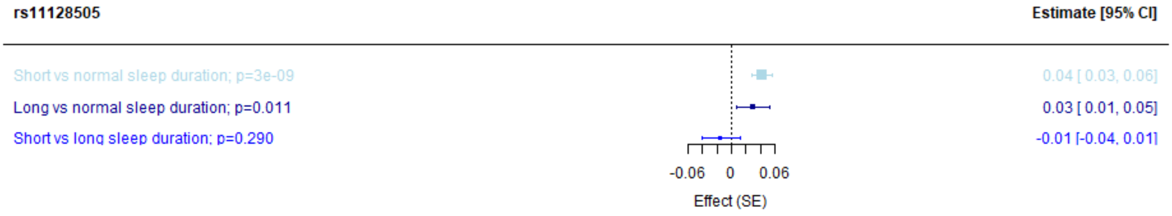
rs11128505
Estimate [95% CI]
Short vs normal sleep duration; p=3e-09
Long vs normal sleep duration; p=0.011
Short vs long sleep duration; p=0.290
0.04 [0.03, 0.06]
0.03 [0.01, 0.05]
-0.01 [-0.04, 0.01]
-0.06
0
0.06
Effect (SE)

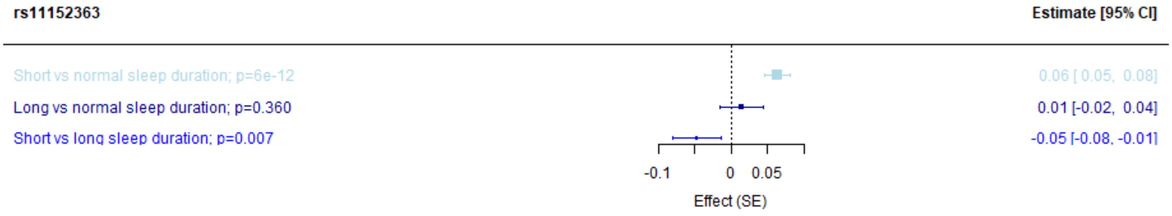
rs11152363
Estimate [95% CI]
Short vs normal sleep duration; p=6e-12
Long vs normal sleep duration; p=0.360
Short vs long sleep duration; p=0.007
0.06 [0.05, 0.08]
0.01 [-0.02, 0.04]
-0.05 [-0.08, -0.01]
-0.1
0
0.05
Effect (SE)

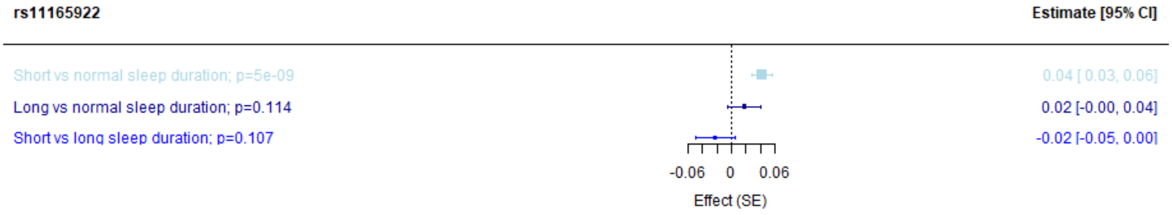
rs11165922
Estimate [95% CI]
Short vs normal sleep duration; p=5e-09
Long vs normal sleep duration; p=0.114
Short vs long sleep duration; p=0.107
0.04 [0.03, 0.06]
0.02 [-0.00, 0.04]
-0.02 [-0.05, 0.00]
-0.06
0
0.06
Effect (SE)

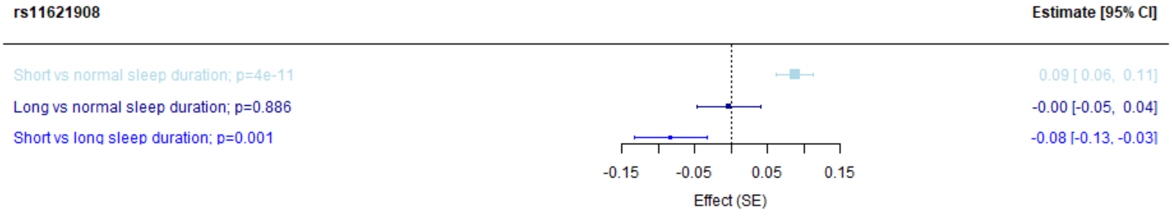
rs11621908
Estimate [95% CI]
Short vs normal sleep duration; p=4e-11
Long vs normal sleep duration; p=0.886
Short vs long sleep duration; p=0.001
0.09 [0.06, 0.11]
-0.00 [-0.05, 0.04]
-0.08 [-0.13, -0.03]
-0.15
-0.05
0.05
0.15
Effect (SE)

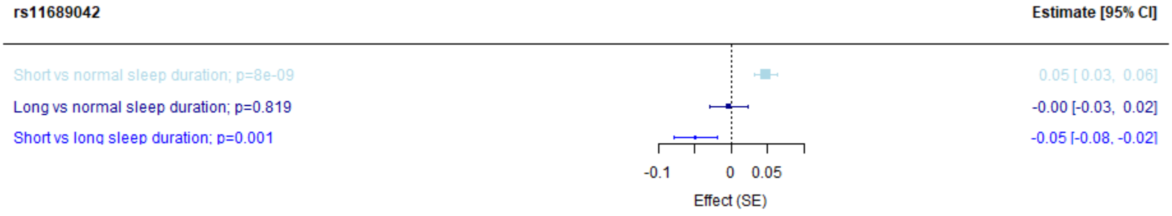
rs11689042
Estimate [95% CI]
Short vs normal sleep duration; p=8e-09
Long vs normal sleep duration; p=0.819
Short vs long sleep duration; p=0.001
0.05 [0.03, 0.06]
-0.00 [-0.03, 0.02]
-0.05 [-0.08, -0.02]
-0.1
0
0.05
Effect (SE)

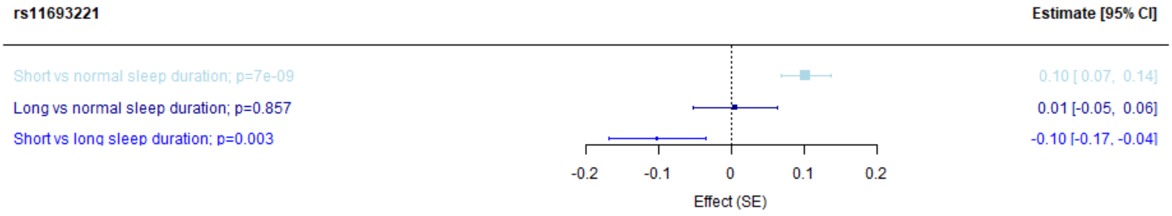
rs11693221
Estimate [95% CI]
Short vs normal sleep duration; p=7e-09
Long vs normal sleep duration; p=0.857
Short vs long sleep duration; p=0.003
0.10 [0.07, 0.14]
0.01 [-0.05, 0.06]
-0.10 [-0.17, -0.04]
-0.2 -0.1 0 0.1 0.2
Effect (SE)

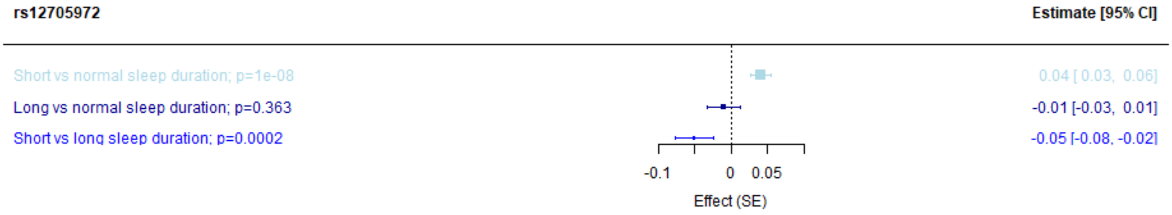
rs12705972
Estimate [95% CI]
Short vs normal sleep duration; p=1e-08
Long vs normal sleep duration; p=0.363
Short vs long sleep duration; p=0.0002
0.04 [0.03, 0.06]
-0.01 [-0.03, 0.01]
-0.05 [-0.08, -0.02]
-0.1 0 0.05
Effect (SE)

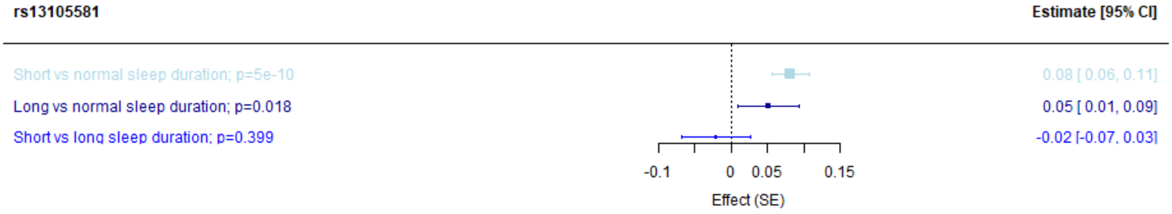
rs13105581
Estimate [95% CI]
Short vs normal sleep duration; p=5e-10
Long vs normal sleep duration; p=0.018
Short vs long sleep duration; p=0.399
0.08 [0.06, 0.11]
0.05 [0.01, 0.09]
-0.02 [-0.07, 0.03]
-0.1 0 0.05 0.15
Effect (SE)

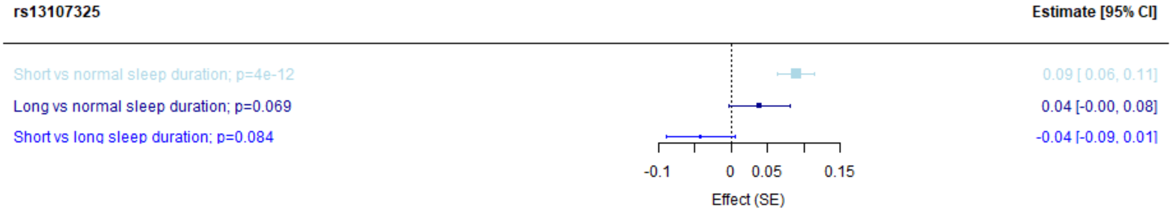
rs13107325
Estimate [95% CI]
Short vs normal sleep duration; p=4e-12
Long vs normal sleep duration; p=0.069
Short vs long sleep duration; p=0.084
0.09 [0.06, 0.11]
0.04 [-0.00, 0.08]
-0.04 [-0.09, 0.01]
-0.1 0 0.05 0.15
Effect (SE)

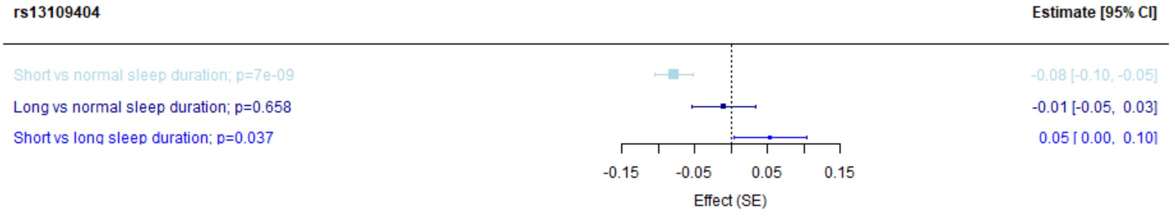
rs13109404
Estimate [95% CI]
Short vs normal sleep duration; p=7e-09
Long vs normal sleep duration; p=0.658
Short vs long sleep duration; p=0.037
-0.08 [-0.10, -0.05]
-0.01 [-0.05, 0.03]
0.05 [0.00, 0.10]
-0.15 -0.05 0.05 0.15
Effect (SE)

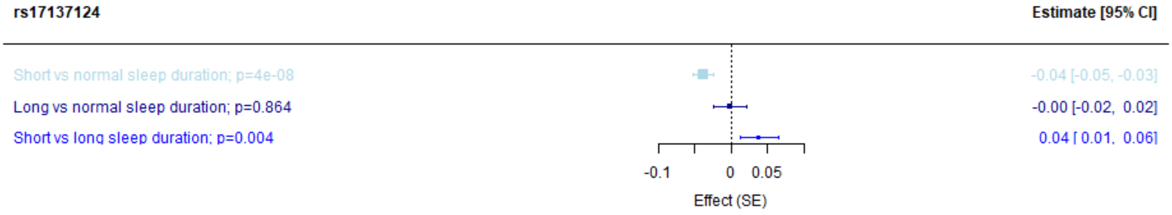
rs17137124
Estimate [95% CI]
Short vs normal sleep duration; p=4e-08
Long vs normal sleep duration; p=0.864
Short vs long sleep duration; p=0.004
-0.04 [-0.05, -0.03]
-0.00 [-0.02, 0.02]
0.04 [0.01, 0.06]
-0.1 0 0.05
Effect (SE)

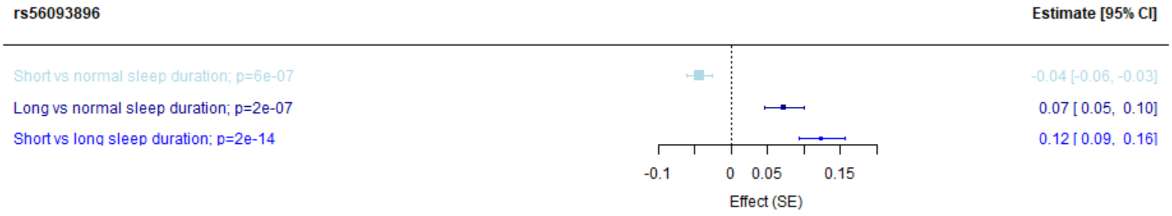
rs56093896
Estimate [95% CI]
Short vs normal sleep duration; p=6e-07
Long vs normal sleep duration; p=2e-07
Short vs long sleep duration; p=2e-14
-0.04 [-0.06, -0.03]
0.07 [0.05, 0.10]
0.12 [0.09, 0.16]
-0.1
0
0.05
0.15
Effect (SE)

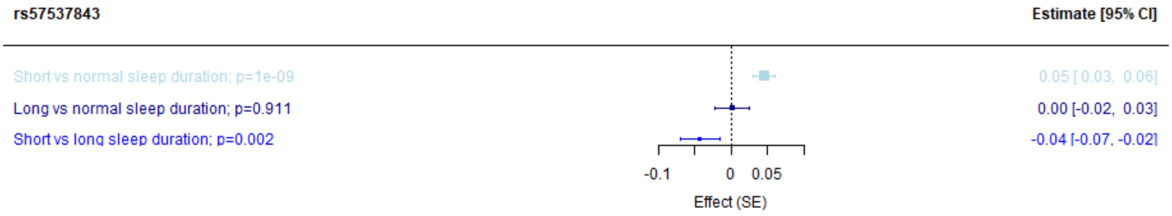
rs57537843
Estimate [95% CI]
Short vs normal sleep duration; p=1e-09
Long vs normal sleep duration; p=0.911
Short vs long sleep duration; p=0.002
0.05 [0.03, 0.06]
0.00 [-0.02, 0.03]
-0.04 [-0.07, -0.02]
-0.1
0
0.05
Effect (SE)

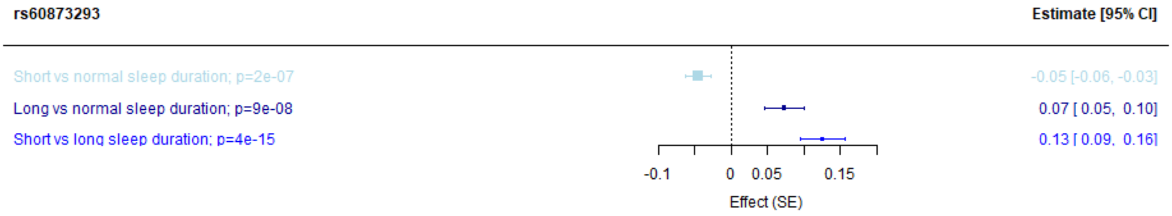
rs60873293
Estimate [95% CI]
Short vs normal sleep duration; p=2e-07
Long vs normal sleep duration; p=9e-08
Short vs long sleep duration; p=4e-15
-0.05 [-0.06, -0.03]
0.07 [0.05, 0.10]
0.13 [0.09, 0.16]
-0.1
0
0.05
0.15
Effect (SE)

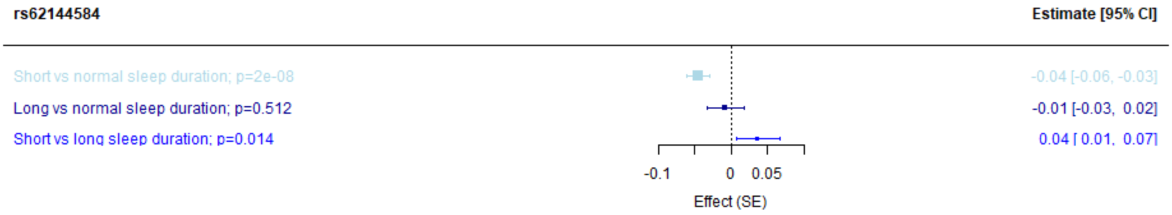
rs62144584
Estimate [95% CI]
Short vs normal sleep duration; p=2e-08
Long vs normal sleep duration; p=0.512
Short vs long sleep duration; p=0.014
-0.04 [-0.06, -0.03]
-0.01 [-0.03, 0.02]
0.04 [0.01, 0.07]
-0.1
0
0.05
Effect (SE)

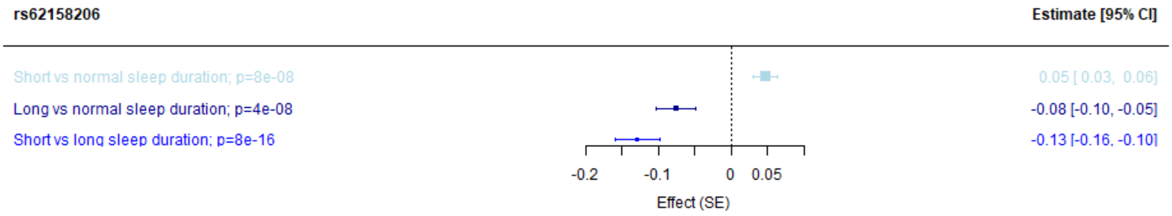
rs62158206
Estimate [95% CI]
Short vs normal sleep duration; p=8e-08
Long vs normal sleep duration; p=4e-08
Short vs long sleep duration; p=8e-16
0.05 [0.03, 0.06]
-0.08 [-0.10, -0.05]
-0.13 [-0.16, -0.10]
-0.2
-0.1
0
0.05
Effect (SE)

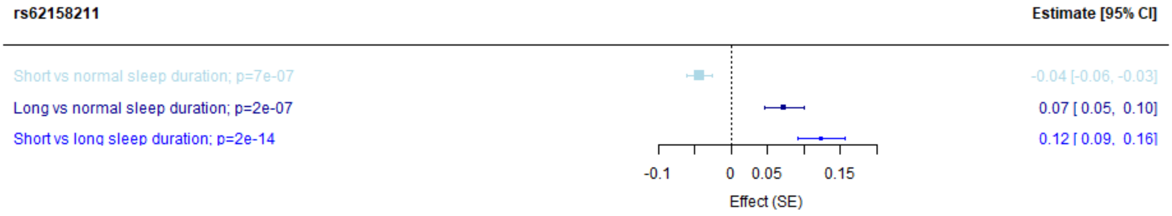
rs62158211
Estimate [95% CI]
Short vs normal sleep duration; p=7e-07
Long vs normal sleep duration; p=2e-07
Short vs long sleep duration; p=2e-14
-0.04 [-0.06, -0.03]
0.07 [0.05, 0.10]
0.12 [0.09, 0.16]
-0.1
0
0.05
0.15
Effect (SE)

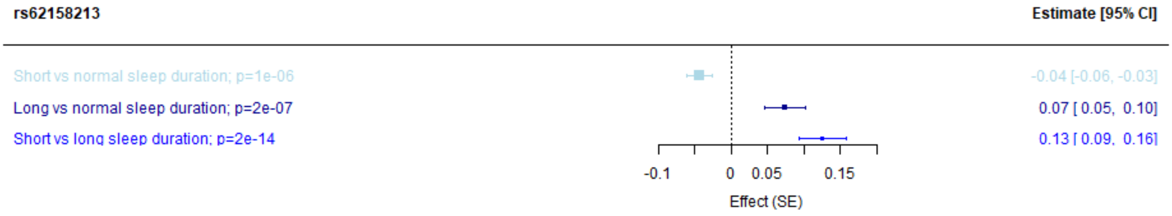
rs62158213
Estimate [95% CI]
Short vs normal sleep duration; p=1e-06
Long vs normal sleep duration; p=2e-07
Short vs long sleep duration; p=2e-14
-0.04 [-0.06, -0.03]
0.07 [0.05, 0.10]
0.13 [0.09, 0.16]
-0.1
0
0.05
0.15
Effect (SE)

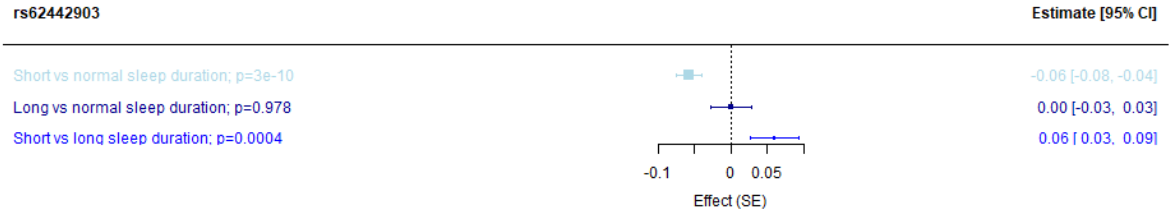
rs62442903
Estimate [95% CI]
Short vs normal sleep duration; p=3e-10
Long vs normal sleep duration; p=0.978
Short vs long sleep duration; p=0.0004
-0.06 [-0.08, -0.04]
0.00 [-0.03, 0.03]
0.06 [0.03, 0.09]
-0.1
0
0.05
Effect (SE)

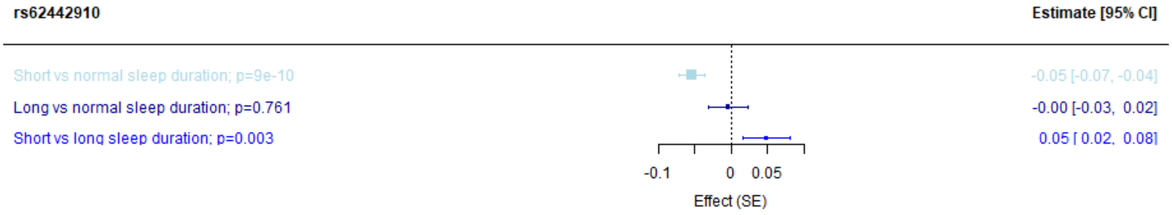
rs62442910
Estimate [95% CI]
Short vs normal sleep duration; p=9e-10
Long vs normal sleep duration; p=0.761
Short vs long sleep duration; p=0.003
-0.05 [-0.07, -0.04]
-0.00 [-0.03, 0.02]
0.05 [0.02, 0.08]
-0.1
0
0.05
Effect (SE)

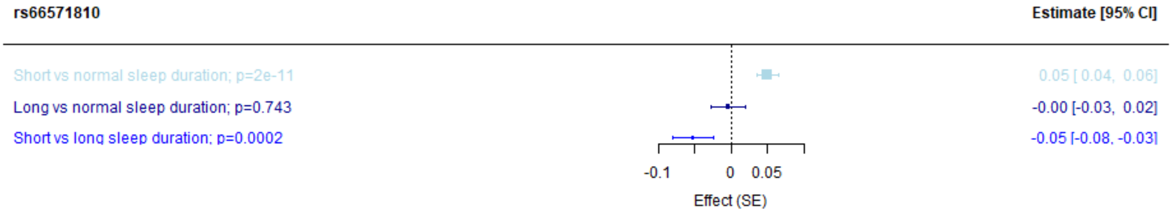
rs66571810
Estimate [95% CI]
Short vs normal sleep duration; p=2e-11
Long vs normal sleep duration; p=0.743
Short vs long sleep duration; p=0.0002
0.05 [0.04, 0.06]
-0.00 [-0.03, 0.02]
-0.05 [-0.08, -0.03]
-0.1
0
0.05
Effect (SE)

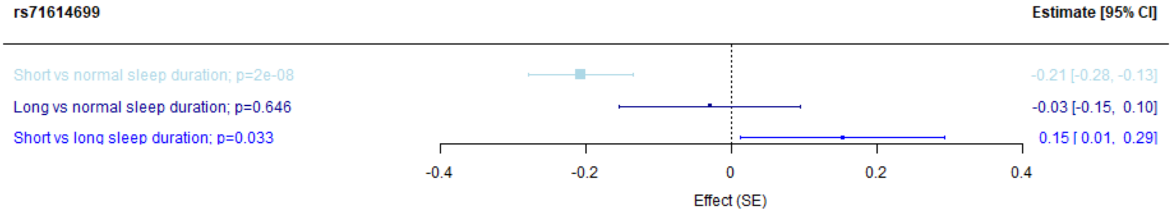
rs71614699
Estimate [95% CI]
Short vs normal sleep duration; p=2e-08
Long vs normal sleep duration; p=0.646
Short vs long sleep duration; p=0.033
-0.21 [-0.28, -0.13]
-0.03 [-0.15, 0.10]
0.15 [0.01, 0.29]
-0.4
-0.2
0
0.2
0.4
Effect (SE)

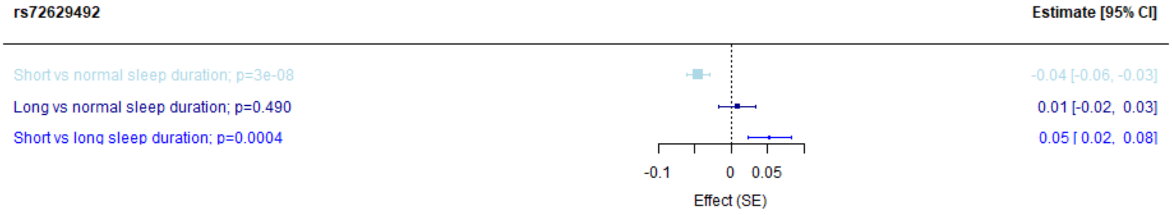
rs72629492
Estimate [95% CI]
Short vs normal sleep duration; p=3e-08
Long vs normal sleep duration; p=0.490
Short vs long sleep duration; p=0.0004
-0.04 [-0.06, -0.03]
0.01 [-0.02, 0.03]
0.05 [0.02, 0.08]
-0.1
0
0.05
Effect (SE)

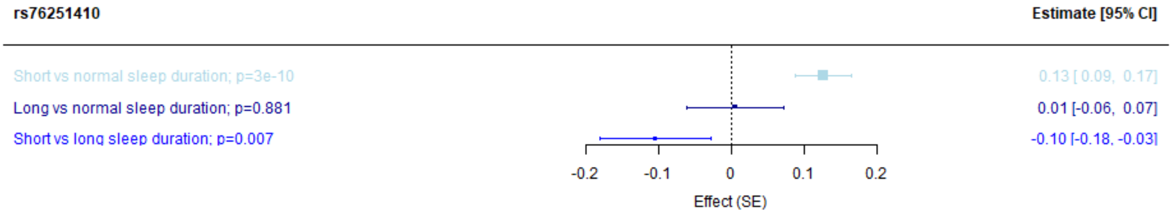
rs76251410
Estimate [95% CI]
Short vs normal sleep duration; p=3e-10
Long vs normal sleep duration; p=0.881
Short vs long sleep duration; p=0.007
0.13 [0.09, 0.17]
0.01 [-0.06, 0.07]
-0.10 [-0.18, -0.03]
-0.2
-0.1
0
0.1
0.2
Effect (SE)

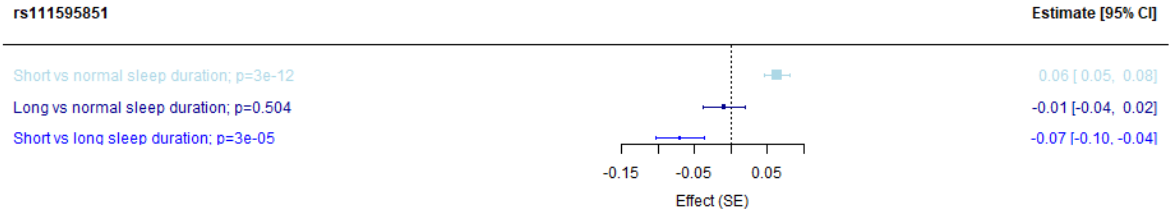
rs111595851
Estimate [95% CI]
Short vs normal sleep duration; p=3e-12
Long vs normal sleep duration; p=0.504
Short vs long sleep duration; p=3e-05
0.06 [0.05, 0.08]
-0.01 [-0.04, 0.02]
-0.07 [-0.10, -0.04]
-0.15
-0.05
0.05
Effect (SE)

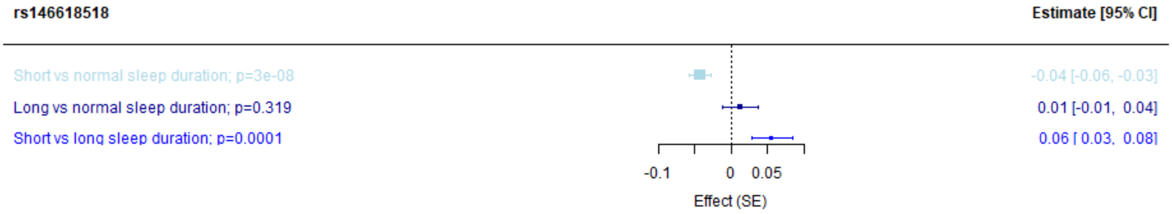
rs146618518
Estimate [95% CI]
Short vs normal sleep duration; p=3e-08
Long vs normal sleep duration; p=0.319
Short vs long sleep duration; p=0.0001
-0.04 [-0.06, -0.03]
0.01 [-0.01, 0.04]
0.06 [0.03, 0.08]
-0.1
0
0.05
Effect (SE)

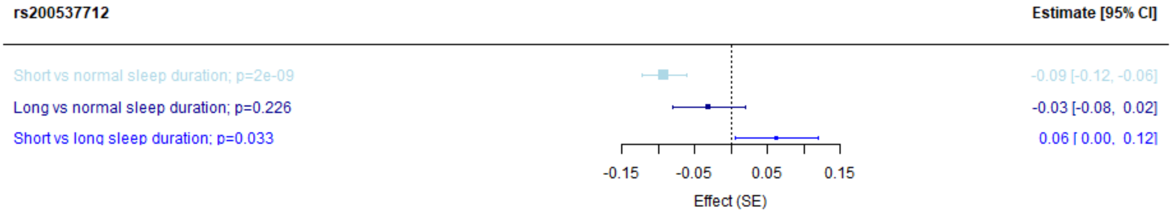
rs200537712
Estimate [95% CI]
Short vs normal sleep duration; p=2e-09
Long vs normal sleep duration; p=0.226
Short vs long sleep duration; p=0.033
-0.09 [-0.12, -0.06]
-0.03 [-0.08, 0.02]
0.06 [0.00, 0.12]
-0.15
-0.05
0.05
0.15
Effect (SE)

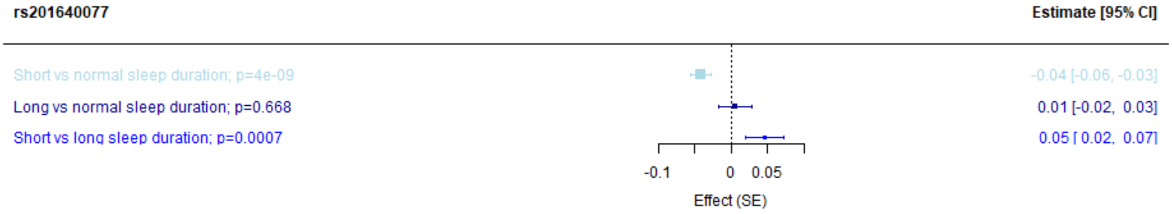
rs201640077
Estimate [95% CI]
Short vs normal sleep duration; p=4e-09
Long vs normal sleep duration; p=0.668
Short vs long sleep duration; p=0.0007
-0.04 [-0.06, -0.03]
0.01 [-0.02, 0.03]
0.05 [0.02, 0.07]
-0.1
0
0.05
Effect (SE)

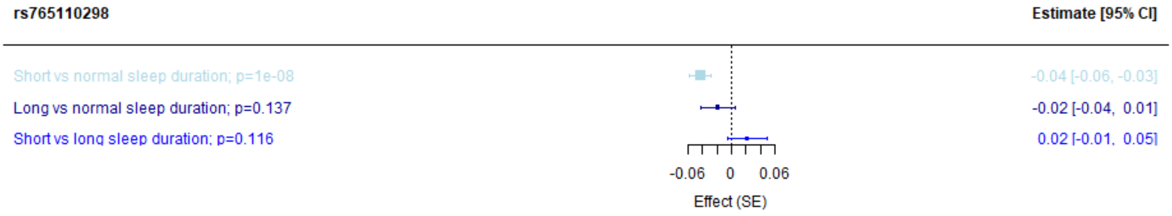
rs765110298
Estimate [95% CI]
Short vs normal sleep duration; p=1e-08
Long vs normal sleep duration; p=0.137
Short vs long sleep duration; p=0.116
-0.04 [-0.06, -0.03]
-0.02 [-0.04, 0.01]
0.02 [-0.01, 0.05]
-0.06
0
0.06
Effect (SE)

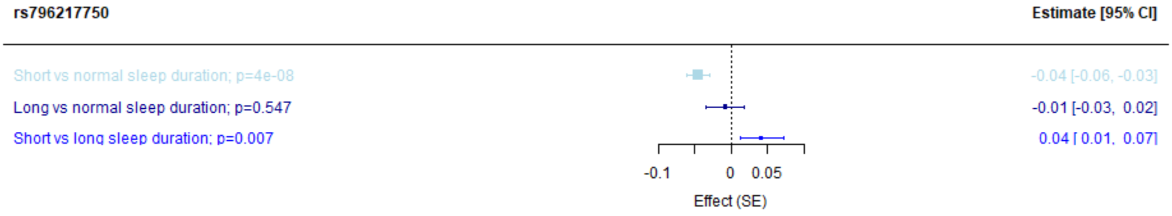
rs796217750
Estimate [95% CI]
Short vs normal sleep duration; p=4e-08
Long vs normal sleep duration; p=0.547
Short vs long sleep duration; p=0.007
-0.04 [-0.06, -0.03]
-0.01 [-0.03, 0.02]
0.04 [0.01, 0.07]
-0.1
0
0.05
Effect (SE)

3. Analysis with nightshift workers excluded

We extracted information on all those participants who responded 'Usually' or 'Always' to the question "Does your work involve night shifts?" (data field 3426; question asked to 51,930 participants who indicated that their work involved shift patterns at least occasionally). A total of 11,087 individuals indicated that their job usually or always involved night shift work, and we re-ran our main analyses on short versus normal and long versus normal sleep duration with these participants excluded. This resulted in a total of N=18,284 short sleep cases, N= 6,445 long sleep cases and N= 250,079 controls. The mirrored Manhattan plots for short (top) and long (bottom) are below (Supplementary Figure 28).

We calculated the genetic correlation (rg) between our primary analyses and these sub-analyses and found the rg to be very close to 1 in both cases (rg short sleep with and without night shift workers = 0.99, SE 0.008, p=0; rg long sleep with and without night shift workers = 1.02, SE 0.0012, p=0 (Supplementary Figure 31). The correlation between long and short sleep duration in this subpopulation was 0.23 (SE=0.060, p=0.0001, as compared to rg 0.24 (SE 0.060, p=$4.74x10^{-5}$ between long and short sleep in the main UK Biobank analysis). Given this near perfect genetic correlation. And considering additional challenges in accessing the necessary phenotype data in the MVP sample, we have conducted this sub-analysis in the UK Biobank cohort only.

Supplementary Figure 28 Mirrored Manhattan plot showing genetic associations with short sleep duration (top) and long sleep duration (bottom) in EUR UK Biobank participants, with participants who report working nightshifts excluded. Loci reaching genome-wide significance threshold of $5x10^{-8}$ highlighted in green.

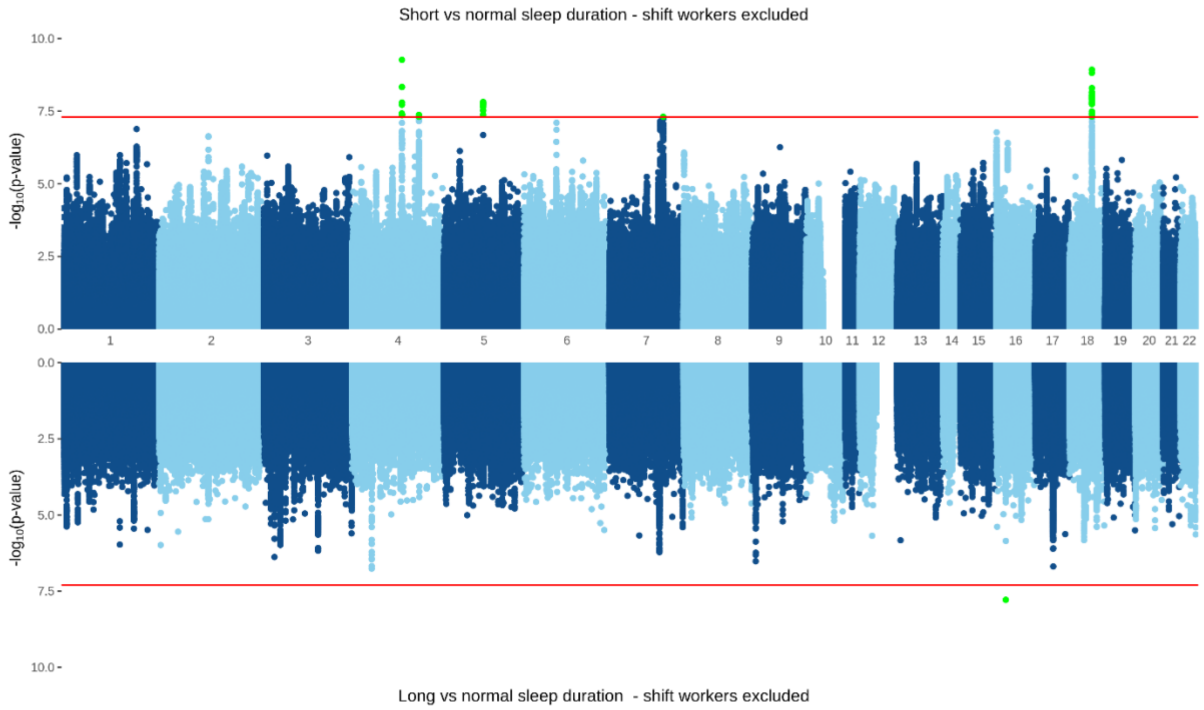

4. Sex-stratified analysis

We conducted further sub-analyses in male only and female only populations. In the UK Biobank only analysis, both sex-stratified analyses were underpowered (female short sleep cases = 10,662, female long sleep cases = 3,565, female controls = 136,242; male short sleep cases = 8,379, male long sleep cases = 2,989, and male controls = 117,978) (Supplementary Figure *29*) and revealed no genome-wide significant associations. The genetic correlation for between these sex-stratified analyses and the main analyses were over 0.97 in all cases (Supplementary Figure *31*).

Supplementary Figure 29 Mirrored Manhattan plot showing genetic associations with short sleep duration (top) and long sleep duration (bottom) in female only EUR UK Biobank participants. Loci reaching genome-wide significance threshold of 5x10-8 highlighted in green.

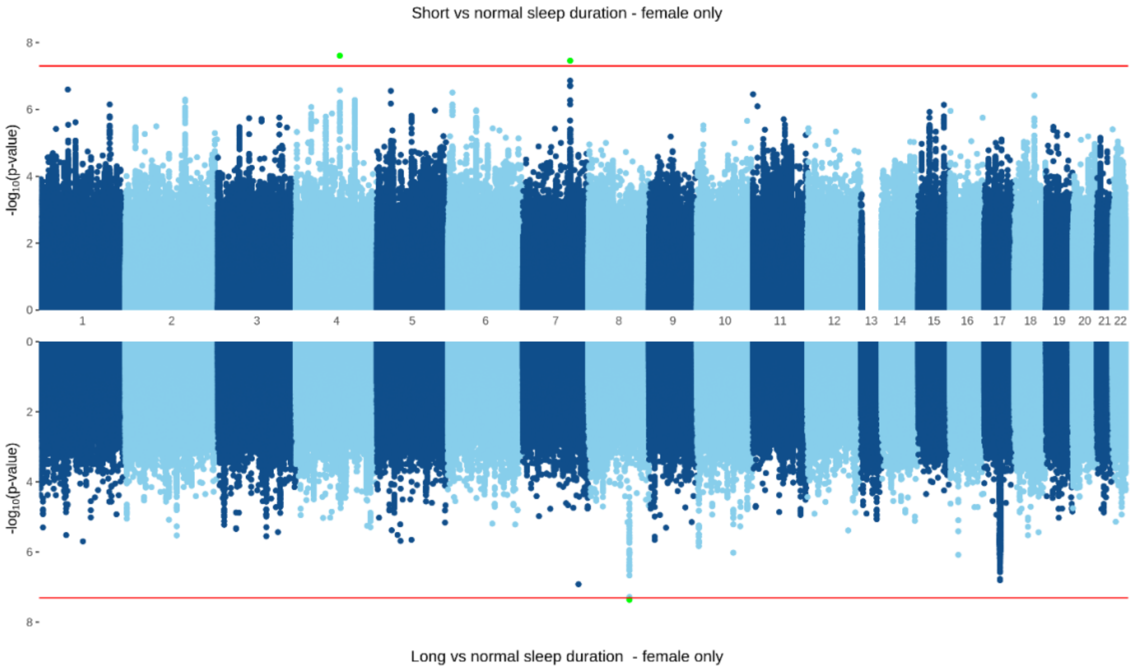

Figure 30 Mirrored Manhattan plot showing genetic associations with short sleep duration (top) and long sleep duration (bottom) in male only EUR UK Biobank participants.

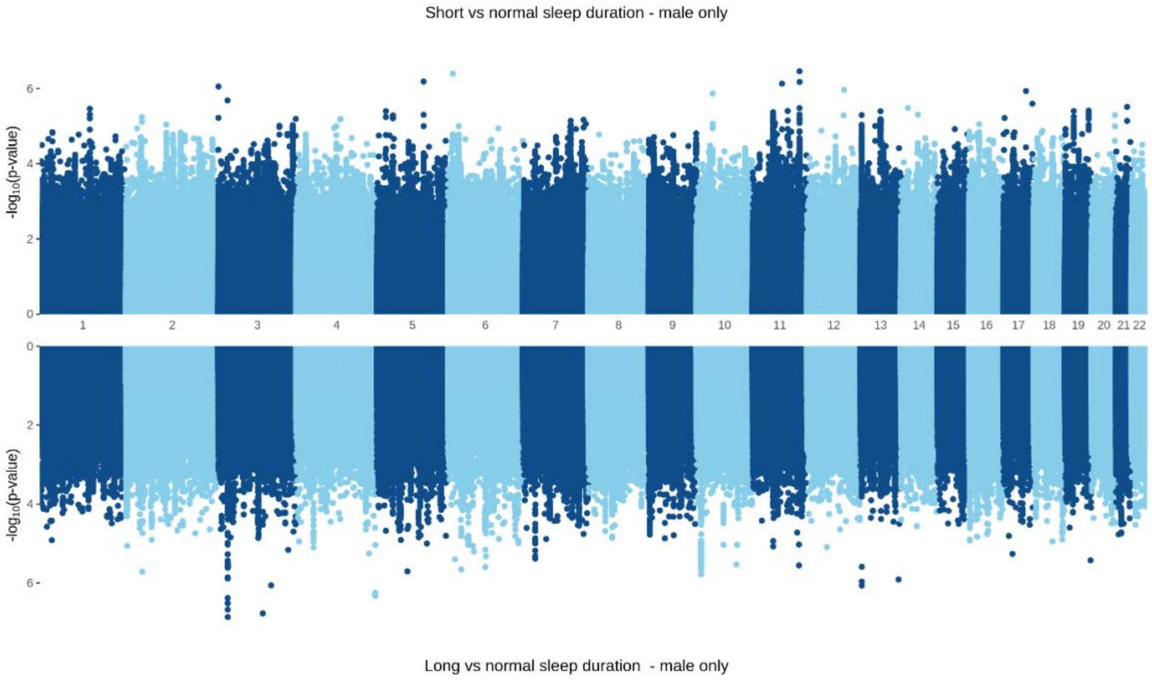

5. Comparison of results between primary EUR analyses and sensitivity analyses

Genetic correlation between main analyses

We calculated the genetic correlation between our main analyses, and the analyses excluding shift workers and stratified by sex. Across all comparisons, the patterns of genetic correlation are very similar. The genetic correlation between all analyses of short sleep is 0.98 or greater. The genetic correlation between all analyses of long sleep is 0.90 or greater. This lower score of 0.90 is between male and female long sleepers.

Similarly, the patterns of genetic correlation between long and short sleep are consistent across all groups, being 0.24 (SE=0.06) in the main analysis, 0.23 (SE=0.06) in the shift worker excluded analysis, 0.32 (SE=0.12) in the male only analysis and 0.26 (SE=0.11) in the female only analysis.

These findings indicate that the genetic architecture of both long and short sleep duration is independent from sex.

Supplementary Figure 31 Genetic correlation analysis between primary analyses of short and long sleep duration and sensitivity analyses of a) shift workers excluded and b) stratified by sex. Darker shades indicate stronger correlation.

	Short sleep - main analysis	Short sleep - shift workers excluded	Short sleep - male only	Short sleep - female only	Long sleep - main analysis	Long sleep - shift workers excluded	Long sleep - male only	Long sleep - female only
**Short sleep - main analysis**		1.00 (0.0008); p=0	0.99 (0.022); p=0	1.00 (0.013); p=0	0.24 (0.060); p=4.74e-05	0.22 (0.067); p=0.0008	0.22 (0.09); p=0.0194	0.25 (0.078); p=0.0014
**Short sleep - shift workers excluded**	1.00 (0.0008); p=0		1.00 (0.026); p=0	1.01 (0.013); p=0	0.26 (0.067); p=0.0001	0.23 (0.060); p=0.0001	0.28 (0.096); p=0.0041	0.23 (0.086); p=0.0077
**Short sleep - male only**	0.99 (0.022); p=0	1.00 (0.026); p=0		0.98 (0.067); p=7.83e-48	0.27 (0.09); p=0.0026	0.26 (0.094); p=0.0051	0.32 (0.12); p=0.0093	0.22 (0.13); p=0.073
**Short sleep - female only**	1.00 (0.013); p=0	1.01 (0.013); p=0	0.98 (0.067); p=7.83e-48		0.24 (0.078); p=0.0019	0.21 (0.086); p=0.015	0.20 (0.11); p=0.078	0.26 (0.11); p=0.014
**Long sleep - main analysis**	0.24 (0.060); p=4.74e-05	0.26 (0.067); p=0.0001	0.27 (0.09); p=0.0026	0.24 (0.078); p=0.0019		1.00 (0.0012); p=0	0.98 (0.068); p=1.77e-47	0.97 (0.049); p=2.24e-87
**Long sleep - shift workers excluded**	0.22 (0.067); p=0.0008	0.23 (0.060); p=0.0001	0.26 (0.094); p=0.0051	0.21 (0.086); p=0.015	1.00 (0.0012); p=0		1.01 (0.079); p=4.48e-38	0.99 (0.06); p=1.051e-60
**Long sleep - male only**	0.22 (0.09); p=0.0194	0.28 (0.096); p=0.0041	0.32 (0.12); p=0.0093	0.20 (0.11); p=0.078	0.98 (0.068); p=1.77e-47	1.01 (0.079); p=4.48e-38		0.90 (0.22); p=6.13e-05
**Long sleep - female only**	0.25 (0.078); p=0.0014	0.23 (0.086); p=0.0077	0.22 (0.13); p=0.073	0.26 (0.11); p=0.014	0.97 (0.049); p=2.24e-87	0.99 (0.06); p=1.051e-60	0.90 (0.22); p=6.13e-05	

Figure 32 Forest plots for genome-wide significant associations from EUR meta-analysis of short vs normal and long vs normal sleep duration in MVP and UKBB samples, and the same association in the UKBB only sensitivity analyses (shift-workers excluded and sex-stratified). Forest plots show the beta-coefficient and standard error for each SNP.

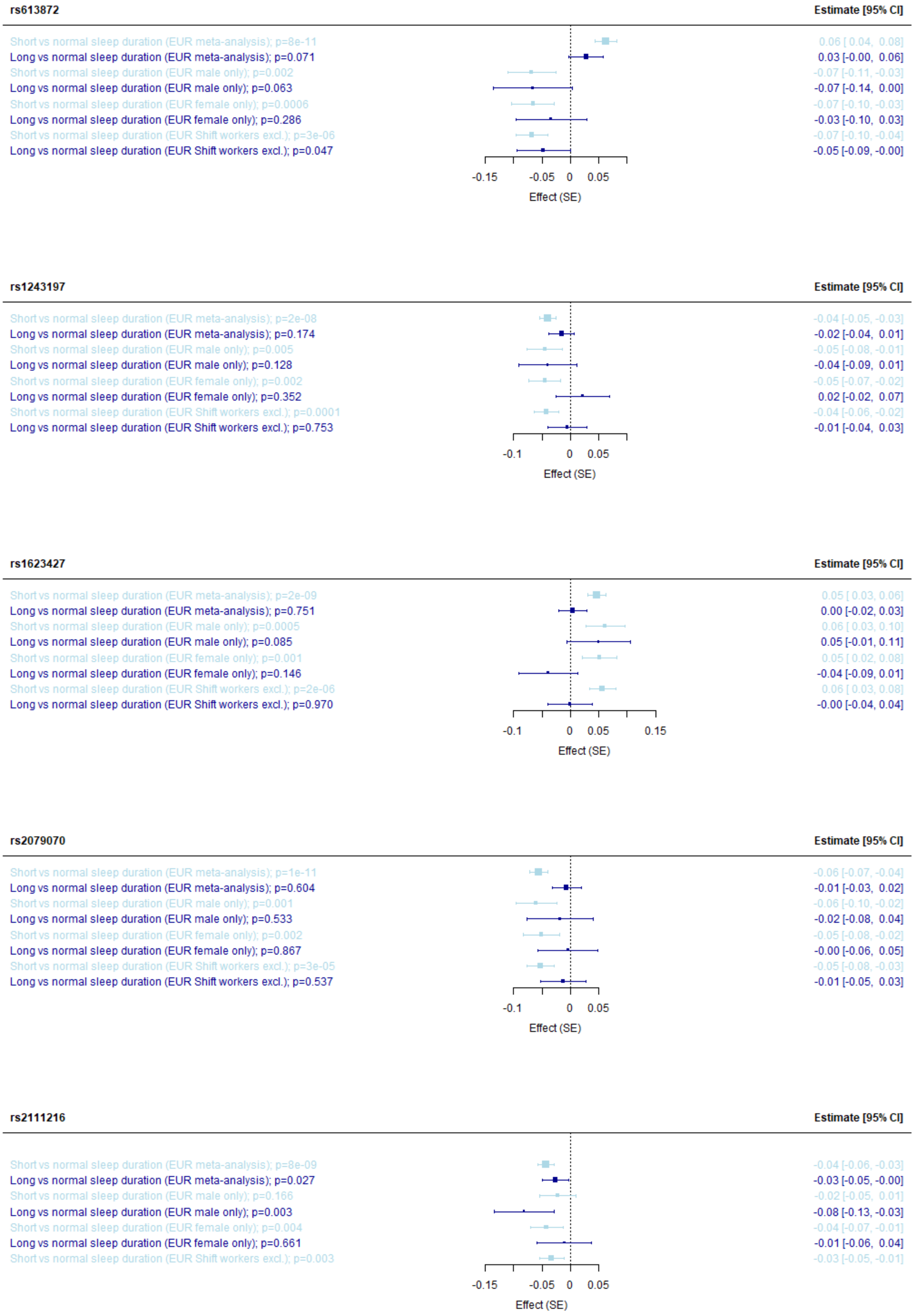

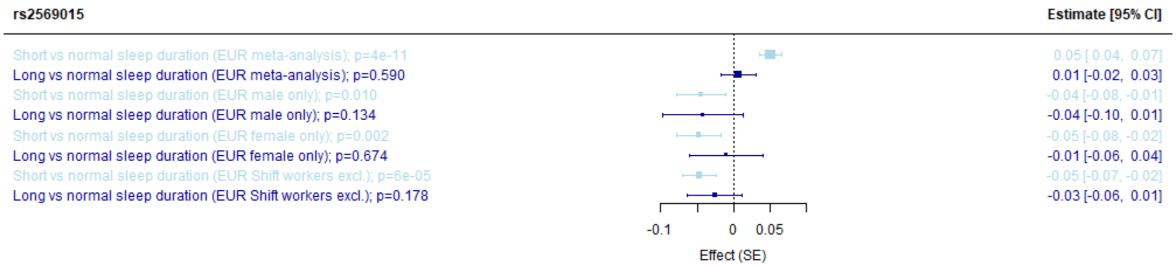
rs2569015
Estimate [95% CI]
Short vs normal sleep duration (EUR meta-analysis); p=4e-11
Long vs normal sleep duration (EUR meta-analysis); p=0.590
Short vs normal sleep duration (EUR male only); p=0.010
Long vs normal sleep duration (EUR male only); p=0.134
Short vs normal sleep duration (EUR female only); p=0.002
Long vs normal sleep duration (EUR female only); p=0.674
Short vs normal sleep duration (EUR Shift workers excl.); p=6e-05
Long vs normal sleep duration (EUR Shift workers excl.); p=0.178
0.05 [0.04, 0.07]
0.01 [-0.02, 0.03]
-0.04 [-0.08, -0.01]
-0.04 [-0.10, 0.01]
-0.05 [-0.08, -0.02]
-0.01 [-0.06, 0.04]
-0.05 [-0.07, -0.02]
-0.03 [-0.06, 0.01]
-0.1
0
0.05
Effect (SE)

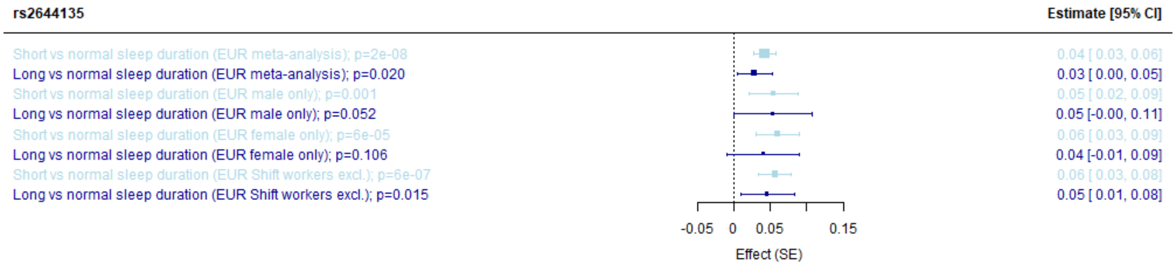
rs2644135
Estimate [95% CI]
Short vs normal sleep duration (EUR meta-analysis); p=2e-08
Long vs normal sleep duration (EUR meta-analysis); p=0.020
Short vs normal sleep duration (EUR male only); p=0.001
Long vs normal sleep duration (EUR male only); p=0.052
Short vs normal sleep duration (EUR female only); p=6e-05
Long vs normal sleep duration (EUR female only); p=0.106
Short vs normal sleep duration (EUR Shift workers excl.); p=6e-07
Long vs normal sleep duration (EUR Shift workers excl.); p=0.015
0.04 [0.03, 0.06]
0.03 [0.00, 0.05]
0.05 [0.02, 0.09]
0.05 [-0.00, 0.11]
0.06 [0.03, 0.09]
0.04 [-0.01, 0.09]
0.06 [0.03, 0.08]
0.05 [0.01, 0.08]
-0.05
0
0.05
0.15
Effect (SE)

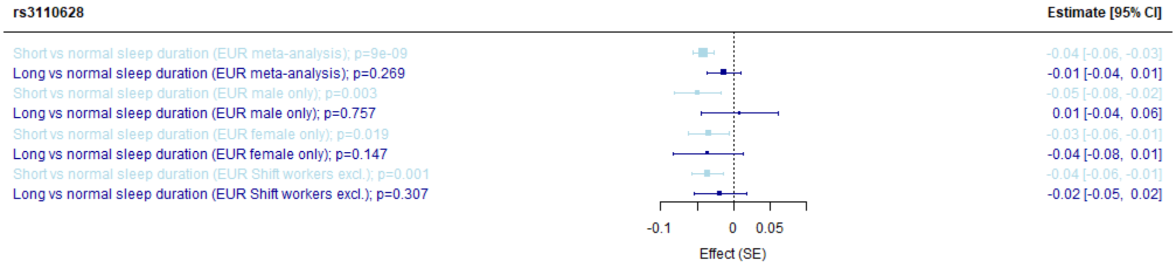
rs3110628
Estimate [95% CI]
Short vs normal sleep duration (EUR meta-analysis); p=9e-09
Long vs normal sleep duration (EUR meta-analysis); p=0.269
Short vs normal sleep duration (EUR male only); p=0.003
Long vs normal sleep duration (EUR male only); p=0.757
Short vs normal sleep duration (EUR female only); p=0.019
Long vs normal sleep duration (EUR female only); p=0.147
Short vs normal sleep duration (EUR Shift workers excl.); p=0.001
Long vs normal sleep duration (EUR Shift workers excl.); p=0.307
-0.04 [-0.06, -0.03]
-0.01 [-0.04, 0.01]
-0.05 [-0.08, -0.02]
0.01 [-0.04, 0.06]
-0.03 [-0.06, -0.01]
-0.04 [-0.08, 0.01]
-0.04 [-0.06, -0.01]
-0.02 [-0.05, 0.02]
-0.1
0
0.05
Effect (SE)

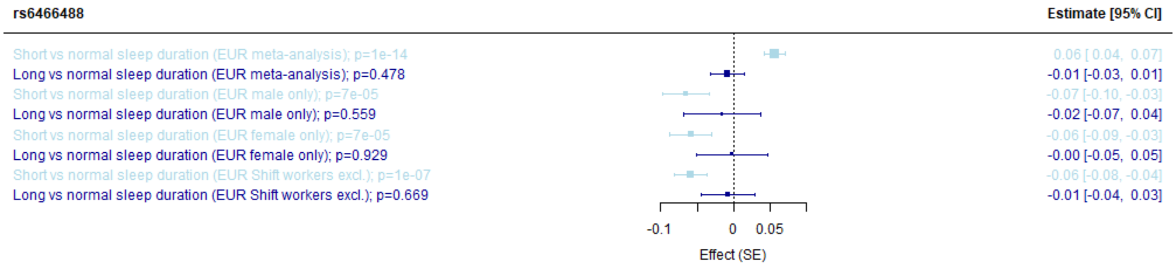
rs6466488
Estimate [95% CI]
Short vs normal sleep duration (EUR meta-analysis); p=1e-14
Long vs normal sleep duration (EUR meta-analysis); p=0.478
Short vs normal sleep duration (EUR male only); p=7e-05
Long vs normal sleep duration (EUR male only); p=0.559
Short vs normal sleep duration (EUR female only); p=7e-05
Long vs normal sleep duration (EUR female only); p=0.929
Short vs normal sleep duration (EUR Shift workers excl.); p=1e-07
Long vs normal sleep duration (EUR Shift workers excl.); p=0.669
0.06 [0.04, 0.07]
-0.01 [-0.03, 0.01]
-0.07 [-0.10, -0.03]
-0.02 [-0.07, 0.04]
-0.06 [-0.09, -0.03]
-0.00 [-0.05, 0.05]
-0.06 [-0.08, -0.04]
-0.01 [-0.04, 0.03]
-0.1
0
0.05
Effect (SE)

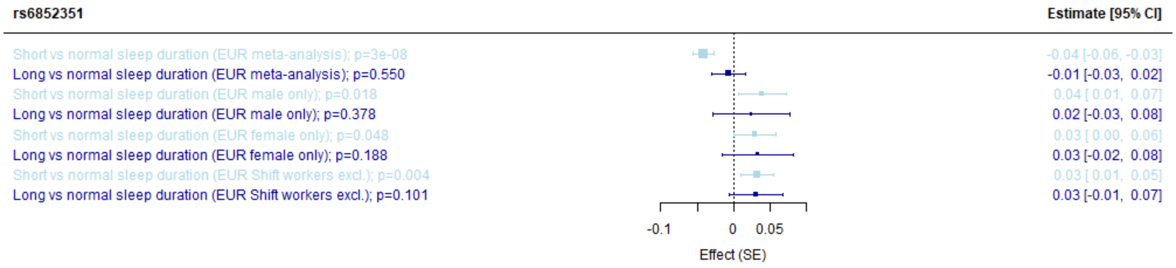
rs6852351
Estimate [95% CI]
Short vs normal sleep duration (EUR meta-analysis); p=3e-08
Long vs normal sleep duration (EUR meta-analysis); p=0.550
Short vs normal sleep duration (EUR male only); p=0.018
Long vs normal sleep duration (EUR male only); p=0.378
Short vs normal sleep duration (EUR female only); p=0.048
Long vs normal sleep duration (EUR female only); p=0.188
Short vs normal sleep duration (EUR Shift workers excl.); p=0.004
Long vs normal sleep duration (EUR Shift workers excl.); p=0.101
-0.04 [-0.06, -0.03]
-0.01 [-0.03, 0.02]
0.04 [0.01, 0.07]
0.02 [-0.03, 0.08]
0.03 [0.00, 0.06]
0.03 [-0.02, 0.08]
0.03 [0.01, 0.05]
0.03 [-0.01, 0.07]
-0.1
0
0.05
Effect (SE)

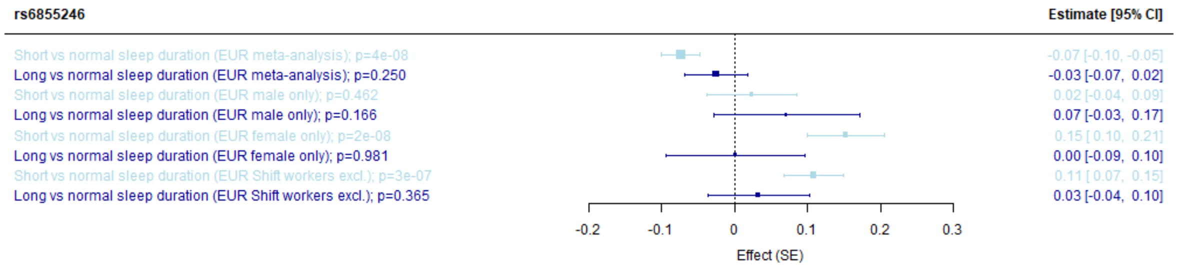

rs6855246
Estimate [95% CI]
Short vs normal sleep duration (EUR meta-analysis); p=4e-08
Long vs normal sleep duration (EUR meta-analysis); p=0.250
Short vs normal sleep duration (EUR male only); p=0.462
Long vs normal sleep duration (EUR male only); p=0.166
Short vs normal sleep duration (EUR female only); p=2e-08
Long vs normal sleep duration (EUR female only); p=0.981
Short vs normal sleep duration (EUR Shift workers excl.); p=3e-07
Long vs normal sleep duration (EUR Shift workers excl.); p=0.365
-0.07 [-0.10, -0.05]
-0.03 [-0.07, 0.02]
0.02 [-0.04, 0.09]
0.07 [-0.03, 0.17]
0.15 [0.10, 0.21]
0.00 [-0.09, 0.10]
0.11 [0.07, 0.15]
0.03 [-0.04, 0.10]
-0.2
-0.1
0
0.1
0.2
0.3
Effect (SE)

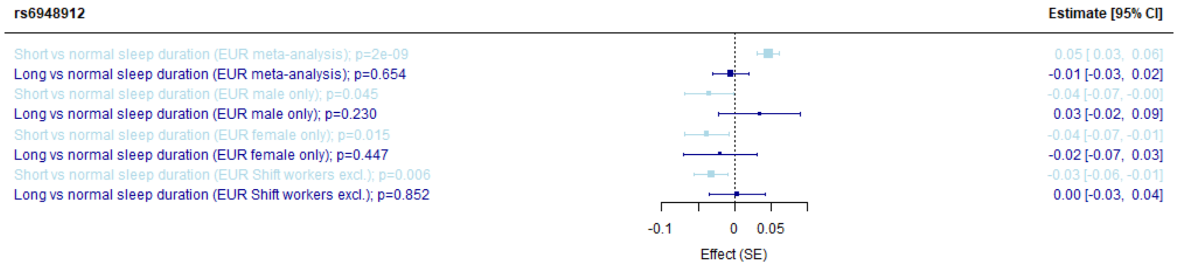

rs6948912
Estimate [95% CI]
Short vs normal sleep duration (EUR meta-analysis); p=2e-09
Long vs normal sleep duration (EUR meta-analysis); p=0.654
Short vs normal sleep duration (EUR male only); p=0.045
Long vs normal sleep duration (EUR male only); p=0.230
Short vs normal sleep duration (EUR female only); p=0.015
Long vs normal sleep duration (EUR female only); p=0.447
Short vs normal sleep duration (EUR Shift workers excl.); p=0.006
Long vs normal sleep duration (EUR Shift workers excl.); p=0.852
0.05 [0.03, 0.06]
-0.01 [-0.03, 0.02]
-0.04 [-0.07, -0.00]
0.03 [-0.02, 0.09]
-0.04 [-0.07, -0.01]
-0.02 [-0.07, 0.03]
-0.03 [-0.06, -0.01]
0.00 [-0.03, 0.04]
-0.1
0
0.05
Effect (SE)

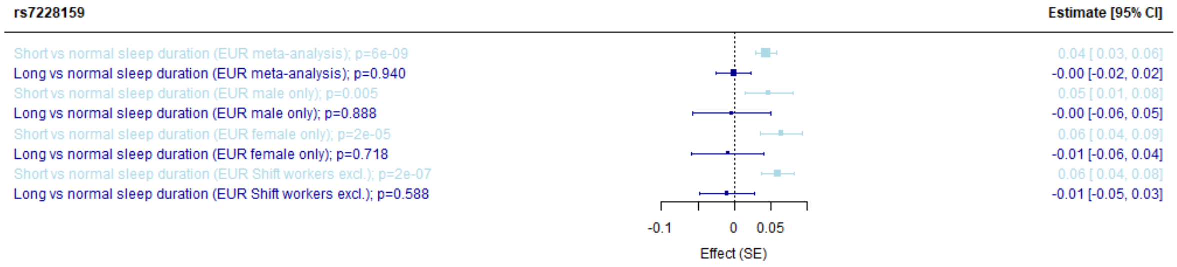

rs7228159
Estimate [95% CI]
Short vs normal sleep duration (EUR meta-analysis); p=6e-09
Long vs normal sleep duration (EUR meta-analysis); p=0.940
Short vs normal sleep duration (EUR male only); p=0.005
Long vs normal sleep duration (EUR male only); p=0.888
Short vs normal sleep duration (EUR female only); p=2e-05
Long vs normal sleep duration (EUR female only); p=0.718
Short vs normal sleep duration (EUR Shift workers excl.); p=2e-07
Long vs normal sleep duration (EUR Shift workers excl.); p=0.588
0.04 [0.03, 0.06]
-0.00 [-0.02, 0.02]
0.05 [0.01, 0.08]
-0.00 [-0.06, 0.05]
0.06 [0.04, 0.09]
-0.01 [-0.06, 0.04]
0.06 [0.04, 0.08]
-0.01 [-0.05, 0.03]
-0.1
0
0.05
Effect (SE)

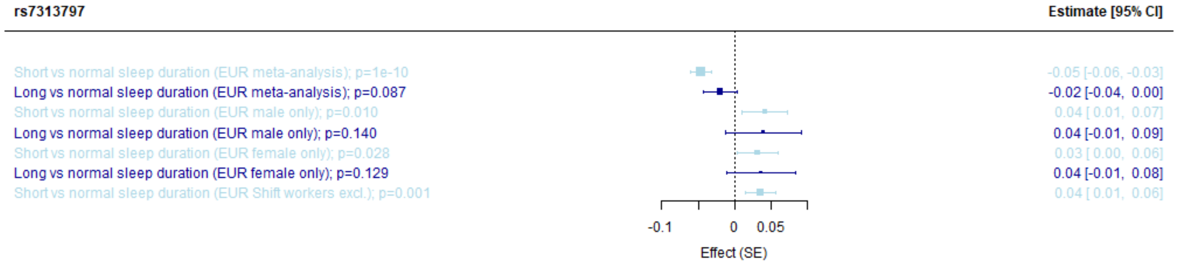

rs7313797
Estimate [95% CI]
Short vs normal sleep duration (EUR meta-analysis); p=1e-10
Long vs normal sleep duration (EUR meta-analysis); p=0.087
Short vs normal sleep duration (EUR male only); p=0.010
Long vs normal sleep duration (EUR male only); p=0.140
Short vs normal sleep duration (EUR female only); p=0.028
Long vs normal sleep duration (EUR female only); p=0.129
Short vs normal sleep duration (EUR Shift workers excl.); p=0.001
-0.05 [-0.06, -0.03]
-0.02 [-0.04, 0.00]
0.04 [0.01, 0.07]
0.04 [-0.01, 0.09]
0.03 [0.00, 0.06]
0.04 [-0.01, 0.08]
0.04 [0.01, 0.06]
-0.1
0
0.05
Effect (SE)

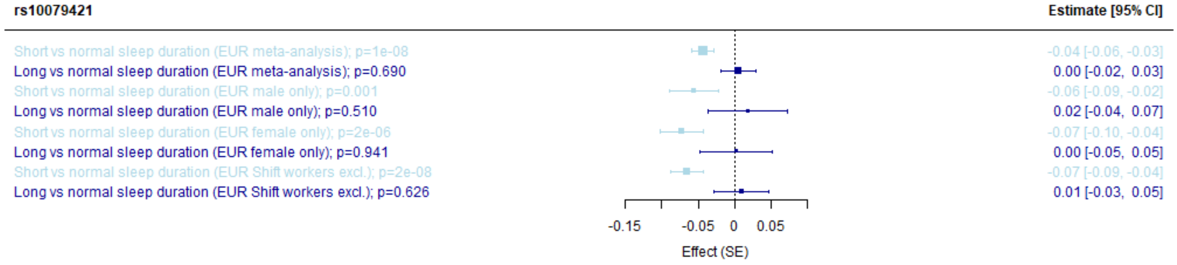

rs10079421
Estimate [95% CI]
Short vs normal sleep duration (EUR meta-analysis); p=1e-08
Long vs normal sleep duration (EUR meta-analysis); p=0.690
Short vs normal sleep duration (EUR male only); p=0.001
Long vs normal sleep duration (EUR male only); p=0.510
Short vs normal sleep duration (EUR female only); p=2e-06
Long vs normal sleep duration (EUR female only); p=0.941
Short vs normal sleep duration (EUR Shift workers excl.); p=2e-08
Long vs normal sleep duration (EUR Shift workers excl.); p=0.626
-0.04 [-0.06, -0.03]
0.00 [-0.02, 0.03]
-0.06 [-0.09, -0.02]
0.02 [-0.04, 0.07]
-0.07 [-0.10, -0.04]
0.00 [-0.05, 0.05]
-0.07 [-0.09, -0.04]
0.01 [-0.03, 0.05]
-0.15
-0.05
0
0.05
Effect (SE)

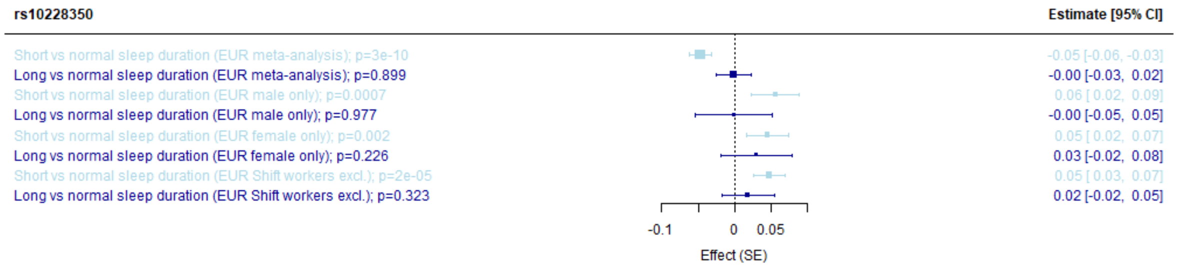
rs10228350
Estimate [95% CI]
Short vs normal sleep duration (EUR meta-analysis); p=3e-10
Long vs normal sleep duration (EUR meta-analysis); p=0.899
Short vs normal sleep duration (EUR male only); p=0.0007
Long vs normal sleep duration (EUR male only); p=0.977
Short vs normal sleep duration (EUR female only); p=0.002
Long vs normal sleep duration (EUR female only); p=0.226
Short vs normal sleep duration (EUR Shift workers excl.); p=2e-05
Long vs normal sleep duration (EUR Shift workers excl.); p=0.323
-0.05 [-0.06, -0.03]
-0.00 [-0.03, 0.02]
0.06 [0.02, 0.09]
-0.00 [-0.05, 0.05]
0.05 [0.02, 0.07]
0.03 [-0.02, 0.08]
0.05 [0.03, 0.07]
0.02 [-0.02, 0.05]
-0.1
0
0.05
Effect (SE)

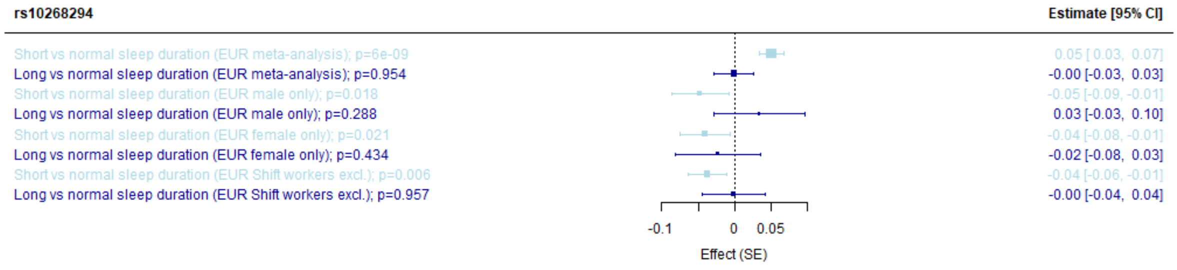
rs10268294
Estimate [95% CI]
Short vs normal sleep duration (EUR meta-analysis); p=6e-09
Long vs normal sleep duration (EUR meta-analysis); p=0.954
Short vs normal sleep duration (EUR male only); p=0.018
Long vs normal sleep duration (EUR male only); p=0.288
Short vs normal sleep duration (EUR female only); p=0.021
Long vs normal sleep duration (EUR female only); p=0.434
Short vs normal sleep duration (EUR Shift workers excl.); p=0.006
Long vs normal sleep duration (EUR Shift workers excl.); p=0.957
0.05 [0.03, 0.07]
-0.00 [-0.03, 0.03]
-0.05 [-0.09, -0.01]
0.03 [-0.03, 0.10]
-0.04 [-0.08, -0.01]
-0.02 [-0.08, 0.03]
-0.04 [-0.06, -0.01]
-0.00 [-0.04, 0.04]
-0.1
0
0.05
Effect (SE)

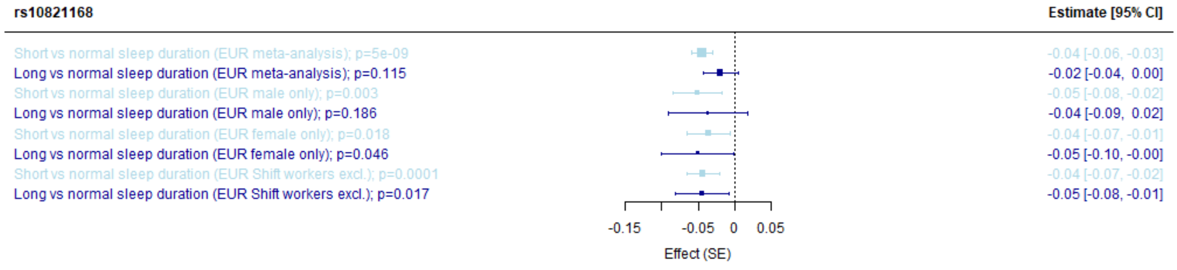
rs10821168
Estimate [95% CI]
Short vs normal sleep duration (EUR meta-analysis); p=5e-09
Long vs normal sleep duration (EUR meta-analysis); p=0.115
Short vs normal sleep duration (EUR male only); p=0.003
Long vs normal sleep duration (EUR male only); p=0.186
Short vs normal sleep duration (EUR female only); p=0.018
Long vs normal sleep duration (EUR female only); p=0.046
Short vs normal sleep duration (EUR Shift workers excl.); p=0.0001
Long vs normal sleep duration (EUR Shift workers excl.); p=0.017
-0.04 [-0.06, -0.03]
-0.02 [-0.04, 0.00]
-0.05 [-0.08, -0.02]
-0.04 [-0.09, 0.02]
-0.04 [-0.07, -0.01]
-0.05 [-0.10, -0.00]
-0.04 [-0.07, -0.02]
-0.05 [-0.08, -0.01]
-0.15
-0.05
0
0.05
Effect (SE)

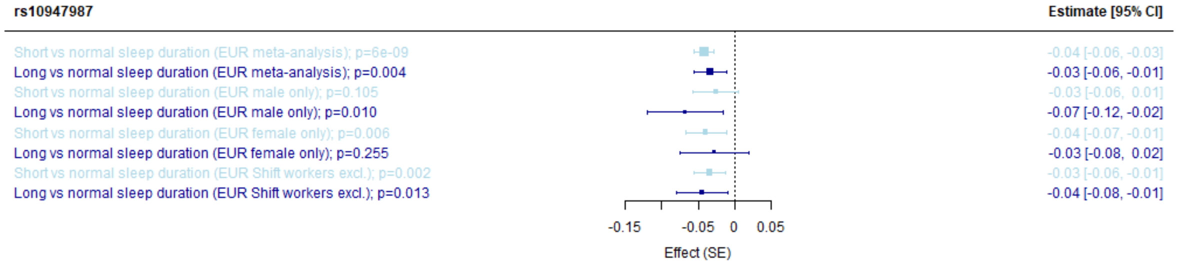
rs10947987
Estimate [95% CI]
Short vs normal sleep duration (EUR meta-analysis); p=6e-09
Long vs normal sleep duration (EUR meta-analysis); p=0.004
Short vs normal sleep duration (EUR male only); p=0.105
Long vs normal sleep duration (EUR male only); p=0.010
Short vs normal sleep duration (EUR female only); p=0.006
Long vs normal sleep duration (EUR female only); p=0.255
Short vs normal sleep duration (EUR Shift workers excl.); p=0.002
Long vs normal sleep duration (EUR Shift workers excl.); p=0.013
-0.04 [-0.06, -0.03]
-0.03 [-0.06, -0.01]
-0.03 [-0.06, 0.01]
-0.07 [-0.12, -0.02]
-0.04 [-0.07, -0.01]
-0.03 [-0.08, 0.02]
-0.03 [-0.06, -0.01]
-0.04 [-0.08, -0.01]
-0.15
-0.05
0
0.05
Effect (SE)

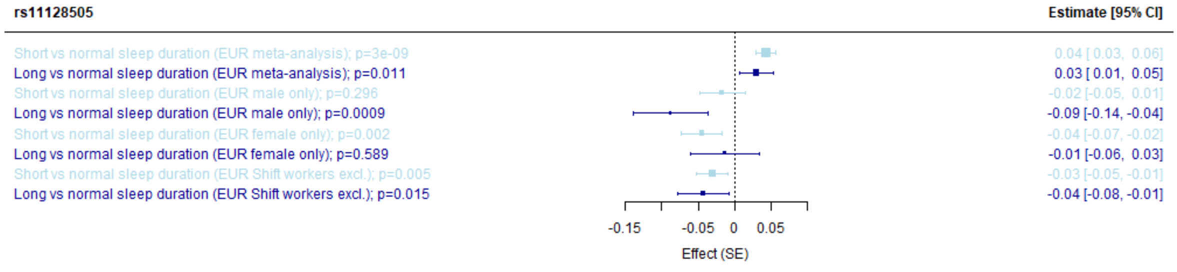
rs11128505
Estimate [95% CI]
Short vs normal sleep duration (EUR meta-analysis); p=3e-09
Long vs normal sleep duration (EUR meta-analysis); p=0.011
Short vs normal sleep duration (EUR male only); p=0.296
Long vs normal sleep duration (EUR male only); p=0.0009
Short vs normal sleep duration (EUR female only); p=0.002
Long vs normal sleep duration (EUR female only); p=0.589
Short vs normal sleep duration (EUR Shift workers excl.); p=0.005
Long vs normal sleep duration (EUR Shift workers excl.); p=0.015
0.04 [0.03, 0.06]
0.03 [0.01, 0.05]
-0.02 [-0.05, 0.01]
-0.09 [-0.14, -0.04]
-0.04 [-0.07, -0.02]
-0.01 [-0.06, 0.03]
-0.03 [-0.05, -0.01]
-0.04 [-0.08, -0.01]
-0.15
-0.05
0
0.05
Effect (SE)

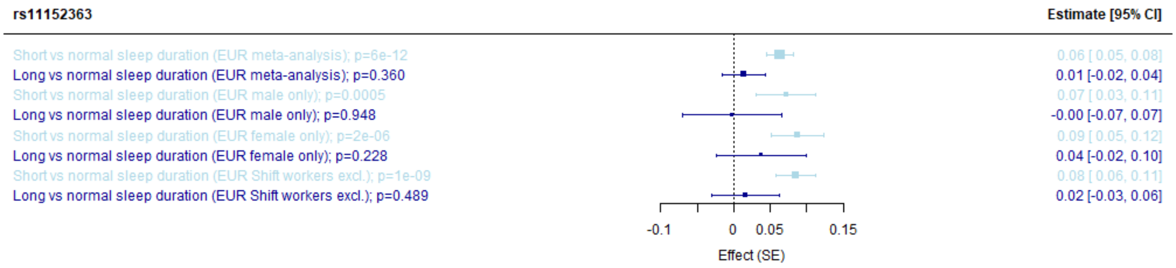
rs11152363
Estimate [95% CI]
Short vs normal sleep duration (EUR meta-analysis); p=6e-12
Long vs normal sleep duration (EUR meta-analysis); p=0.360
Short vs normal sleep duration (EUR male only); p=0.0005
Long vs normal sleep duration (EUR male only); p=0.948
Short vs normal sleep duration (EUR female only); p=2e-06
Long vs normal sleep duration (EUR female only); p=0.228
Short vs normal sleep duration (EUR Shift workers excl.); p=1e-09
Long vs normal sleep duration (EUR Shift workers excl.); p=0.489
0.06 [0.05, 0.08]
0.01 [-0.02, 0.04]
0.07 [0.03, 0.11]
-0.00 [-0.07, 0.07]
0.09 [0.05, 0.12]
0.04 [-0.02, 0.10]
0.08 [0.06, 0.11]
0.02 [-0.03, 0.06]
-0.1
0
0.05
0.15
Effect (SE)

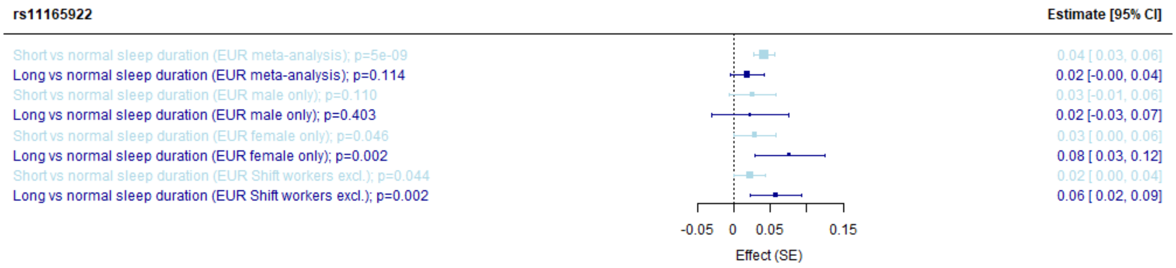
rs11165922
Estimate [95% CI]
Short vs normal sleep duration (EUR meta-analysis); p=5e-09
Long vs normal sleep duration (EUR meta-analysis); p=0.114
Short vs normal sleep duration (EUR male only); p=0.110
Long vs normal sleep duration (EUR male only); p=0.403
Short vs normal sleep duration (EUR female only); p=0.046
Long vs normal sleep duration (EUR female only); p=0.002
Short vs normal sleep duration (EUR Shift workers excl.); p=0.044
Long vs normal sleep duration (EUR Shift workers excl.); p=0.002
0.04 [0.03, 0.06]
0.02 [-0.00, 0.04]
0.03 [-0.01, 0.06]
0.02 [-0.03, 0.07]
0.03 [0.00, 0.06]
0.08 [0.03, 0.12]
0.02 [0.00, 0.04]
0.06 [0.02, 0.09]
-0.05
0
0.05
0.15
Effect (SE)

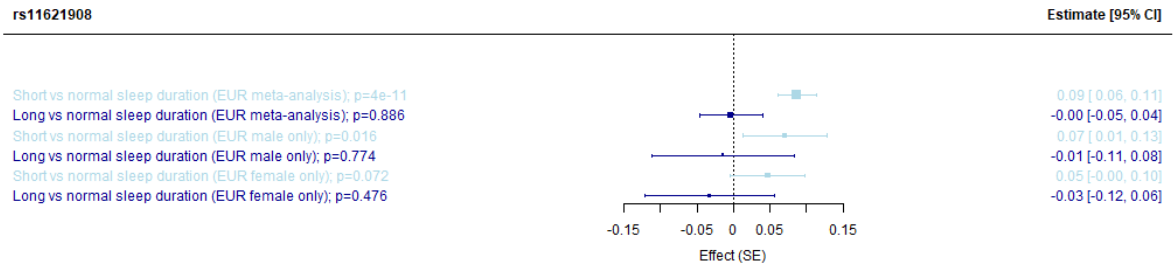
rs11621908
Estimate [95% CI]
Short vs normal sleep duration (EUR meta-analysis); p=4e-11
Long vs normal sleep duration (EUR meta-analysis); p=0.886
Short vs normal sleep duration (EUR male only); p=0.016
Long vs normal sleep duration (EUR male only); p=0.774
Short vs normal sleep duration (EUR female only); p=0.072
Long vs normal sleep duration (EUR female only); p=0.476
0.09 [0.06, 0.11]
-0.00 [-0.05, 0.04]
0.07 [0.01, 0.13]
-0.01 [-0.11, 0.08]
0.05 [-0.00, 0.10]
-0.03 [-0.12, 0.06]
-0.15
-0.05
0
0.05
0.15
Effect (SE)

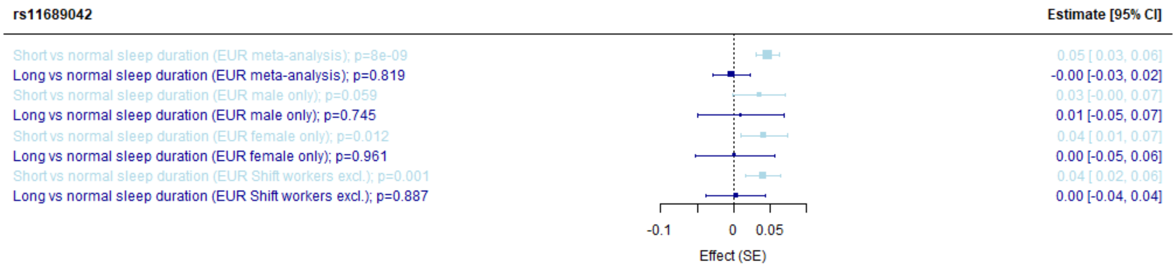
rs11689042
Estimate [95% CI]
Short vs normal sleep duration (EUR meta-analysis); p=8e-09
Long vs normal sleep duration (EUR meta-analysis); p=0.819
Short vs normal sleep duration (EUR male only); p=0.059
Long vs normal sleep duration (EUR male only); p=0.745
Short vs normal sleep duration (EUR female only); p=0.012
Long vs normal sleep duration (EUR female only); p=0.961
Short vs normal sleep duration (EUR Shift workers excl.); p=0.001
Long vs normal sleep duration (EUR Shift workers excl.); p=0.887
0.05 [0.03, 0.06]
-0.00 [-0.03, 0.02]
0.03 [-0.00, 0.07]
0.01 [-0.05, 0.07]
0.04 [0.01, 0.07]
0.00 [-0.05, 0.06]
0.04 [0.02, 0.06]
0.00 [-0.04, 0.04]
-0.1
0
0.05
Effect (SE)

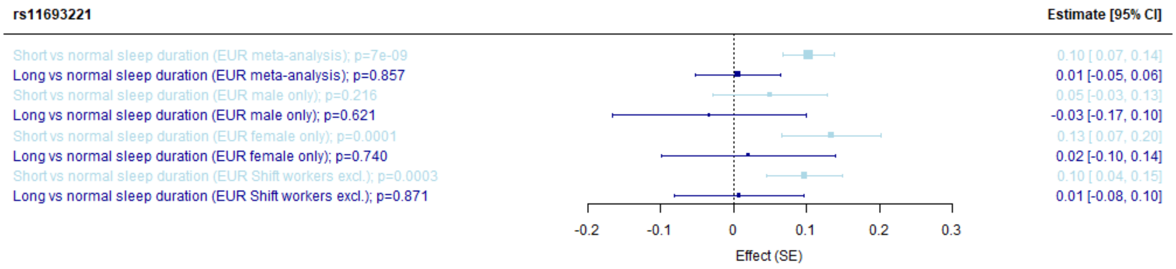
rs11693221
Estimate [95% CI]
Short vs normal sleep duration (EUR meta-analysis); p=7e-09
Long vs normal sleep duration (EUR meta-analysis); p=0.857
Short vs normal sleep duration (EUR male only); p=0.216
Long vs normal sleep duration (EUR male only); p=0.621
Short vs normal sleep duration (EUR female only); p=0.0001
Long vs normal sleep duration (EUR female only); p=0.740
Short vs normal sleep duration (EUR Shift workers excl.); p=0.0003
Long vs normal sleep duration (EUR Shift workers excl.); p=0.871
0.10 [0.07, 0.14]
0.01 [-0.05, 0.06]
0.05 [-0.03, 0.13]
-0.03 [-0.17, 0.10]
0.13 [0.07, 0.20]
0.02 [-0.10, 0.14]
0.10 [0.04, 0.15]
0.01 [-0.08, 0.10]
-0.2
-0.1
0
0.1
0.2
0.3
Effect (SE)

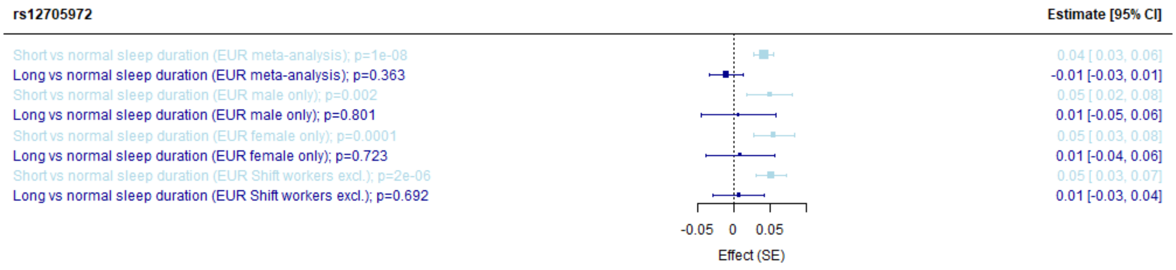
rs12705972
Estimate [95% CI]
Short vs normal sleep duration (EUR meta-analysis); p=1e-08
Long vs normal sleep duration (EUR meta-analysis); p=0.363
Short vs normal sleep duration (EUR male only); p=0.002
Long vs normal sleep duration (EUR male only); p=0.801
Short vs normal sleep duration (EUR female only); p=0.0001
Long vs normal sleep duration (EUR female only); p=0.723
Short vs normal sleep duration (EUR Shift workers excl.); p=2e-06
Long vs normal sleep duration (EUR Shift workers excl.); p=0.692
0.04 [0.03, 0.06]
-0.01 [-0.03, 0.01]
0.05 [0.02, 0.08]
0.01 [-0.05, 0.06]
0.05 [0.03, 0.08]
0.01 [-0.04, 0.06]
0.05 [0.03, 0.07]
0.01 [-0.03, 0.04]
-0.05
0
0.05
Effect (SE)

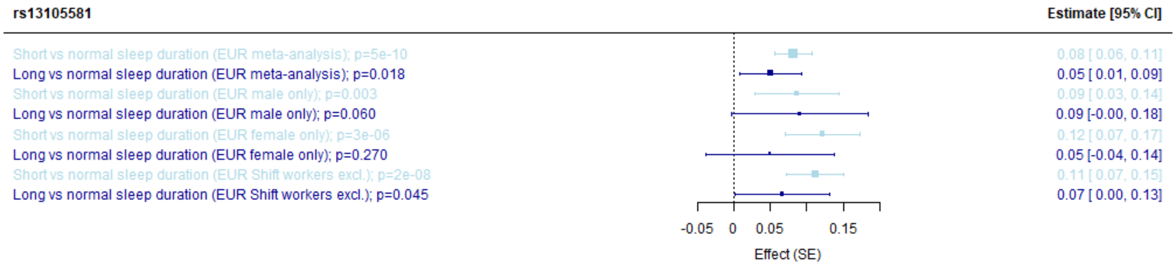
rs13105581
Estimate [95% CI]
Short vs normal sleep duration (EUR meta-analysis); p=5e-10
Long vs normal sleep duration (EUR meta-analysis); p=0.018
Short vs normal sleep duration (EUR male only); p=0.003
Long vs normal sleep duration (EUR male only); p=0.060
Short vs normal sleep duration (EUR female only); p=3e-06
Long vs normal sleep duration (EUR female only); p=0.270
Short vs normal sleep duration (EUR Shift workers excl.); p=2e-08
Long vs normal sleep duration (EUR Shift workers excl.); p=0.045
0.08 [0.06, 0.11]
0.05 [0.01, 0.09]
0.09 [0.03, 0.14]
0.09 [-0.00, 0.18]
0.12 [0.07, 0.17]
0.05 [-0.04, 0.14]
0.11 [0.07, 0.15]
0.07 [0.00, 0.13]
-0.05
0
0.05
0.15
Effect (SE)

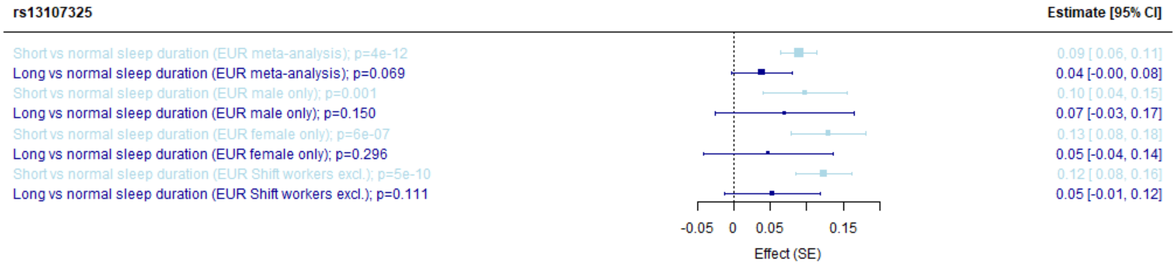
rs13107325
Estimate [95% CI]
Short vs normal sleep duration (EUR meta-analysis); p=4e-12
Long vs normal sleep duration (EUR meta-analysis); p=0.069
Short vs normal sleep duration (EUR male only); p=0.001
Long vs normal sleep duration (EUR male only); p=0.150
Short vs normal sleep duration (EUR female only); p=6e-07
Long vs normal sleep duration (EUR female only); p=0.296
Short vs normal sleep duration (EUR Shift workers excl.); p=5e-10
Long vs normal sleep duration (EUR Shift workers excl.); p=0.111
0.09 [0.06, 0.11]
0.04 [-0.00, 0.08]
0.10 [0.04, 0.15]
0.07 [-0.03, 0.17]
0.13 [0.08, 0.18]
0.05 [-0.04, 0.14]
0.12 [0.08, 0.16]
0.05 [-0.01, 0.12]
-0.05
0
0.05
0.15
Effect (SE)

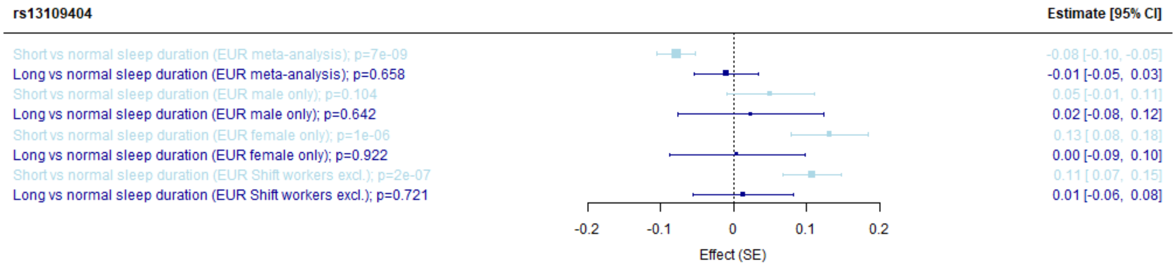
rs13109404
Estimate [95% CI]
Short vs normal sleep duration (EUR meta-analysis); p=7e-09
Long vs normal sleep duration (EUR meta-analysis); p=0.658
Short vs normal sleep duration (EUR male only); p=0.104
Long vs normal sleep duration (EUR male only); p=0.642
Short vs normal sleep duration (EUR female only); p=1e-06
Long vs normal sleep duration (EUR female only); p=0.922
Short vs normal sleep duration (EUR Shift workers excl.); p=2e-07
Long vs normal sleep duration (EUR Shift workers excl.); p=0.721
-0.08 [-0.10, -0.05]
-0.01 [-0.05, 0.03]
0.05 [-0.01, 0.11]
0.02 [-0.08, 0.12]
0.13 [0.08, 0.18]
0.00 [-0.09, 0.10]
0.11 [0.07, 0.15]
0.01 [-0.06, 0.08]
-0.2
-0.1
0
0.1
0.2
Effect (SE)

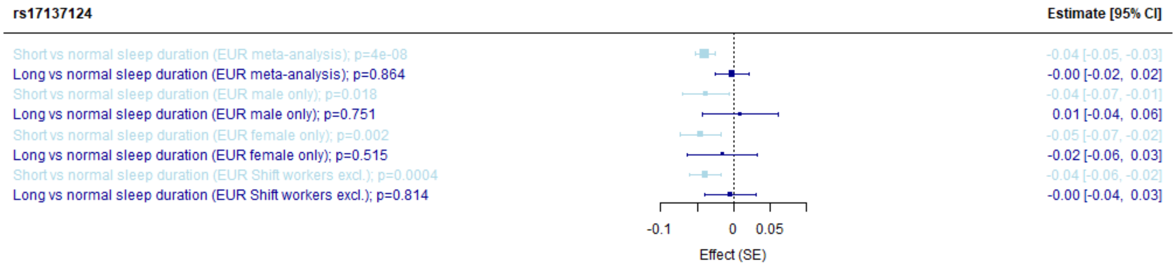
rs17137124
Estimate [95% CI]
Short vs normal sleep duration (EUR meta-analysis); p=4e-08
Long vs normal sleep duration (EUR meta-analysis); p=0.864
Short vs normal sleep duration (EUR male only); p=0.018
Long vs normal sleep duration (EUR male only); p=0.751
Short vs normal sleep duration (EUR female only); p=0.002
Long vs normal sleep duration (EUR female only); p=0.515
Short vs normal sleep duration (EUR Shift workers excl.); p=0.0004
Long vs normal sleep duration (EUR Shift workers excl.); p=0.814
-0.04 [-0.05, -0.03]
-0.00 [-0.02, 0.02]
-0.04 [-0.07, -0.01]
0.01 [-0.04, 0.06]
-0.05 [-0.07, -0.02]
-0.02 [-0.06, 0.03]
-0.04 [-0.06, -0.02]
-0.00 [-0.04, 0.03]
-0.1
0
0.05
Effect (SE)

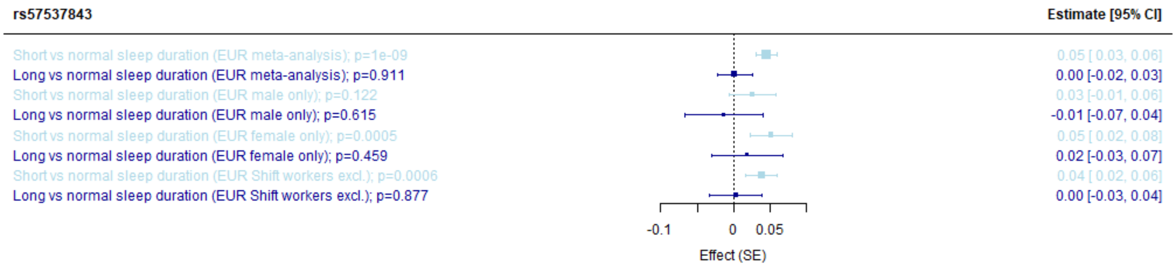
rs57537843
Estimate [95% CI]
Short vs normal sleep duration (EUR meta-analysis); p=1e-09
Long vs normal sleep duration (EUR meta-analysis); p=0.911
Short vs normal sleep duration (EUR male only); p=0.122
Long vs normal sleep duration (EUR male only); p=0.615
Short vs normal sleep duration (EUR female only); p=0.0005
Long vs normal sleep duration (EUR female only); p=0.459
Short vs normal sleep duration (EUR Shift workers excl.); p=0.0006
Long vs normal sleep duration (EUR Shift workers excl.); p=0.877
0.05 [0.03, 0.06]
0.00 [-0.02, 0.03]
0.03 [-0.01, 0.06]
-0.01 [-0.07, 0.04]
0.05 [0.02, 0.08]
0.02 [-0.03, 0.07]
0.04 [0.02, 0.06]
0.00 [-0.03, 0.04]
-0.1
0
0.05
Effect (SE)

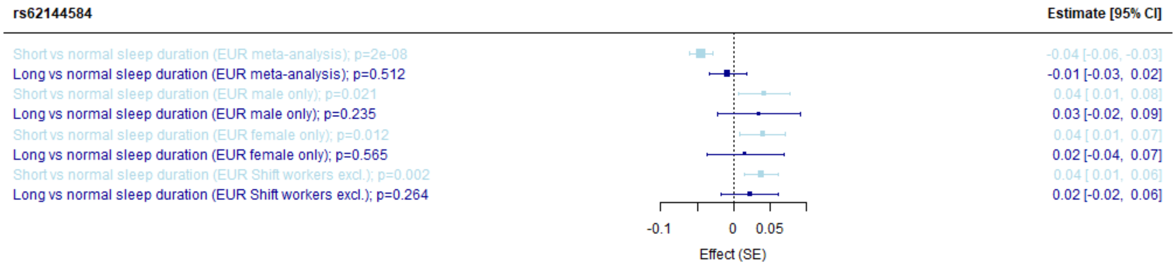
rs62144584
Estimate [95% CI]
Short vs normal sleep duration (EUR meta-analysis); p=2e-08
Long vs normal sleep duration (EUR meta-analysis); p=0.512
Short vs normal sleep duration (EUR male only); p=0.021
Long vs normal sleep duration (EUR male only); p=0.235
Short vs normal sleep duration (EUR female only); p=0.012
Long vs normal sleep duration (EUR female only); p=0.565
Short vs normal sleep duration (EUR Shift workers excl.); p=0.002
Long vs normal sleep duration (EUR Shift workers excl.); p=0.264
-0.04 [-0.06, -0.03]
-0.01 [-0.03, 0.02]
0.04 [0.01, 0.08]
0.03 [-0.02, 0.09]
0.04 [0.01, 0.07]
0.02 [-0.04, 0.07]
0.04 [0.01, 0.06]
0.02 [-0.02, 0.06]
-0.1
0
0.05
Effect (SE)

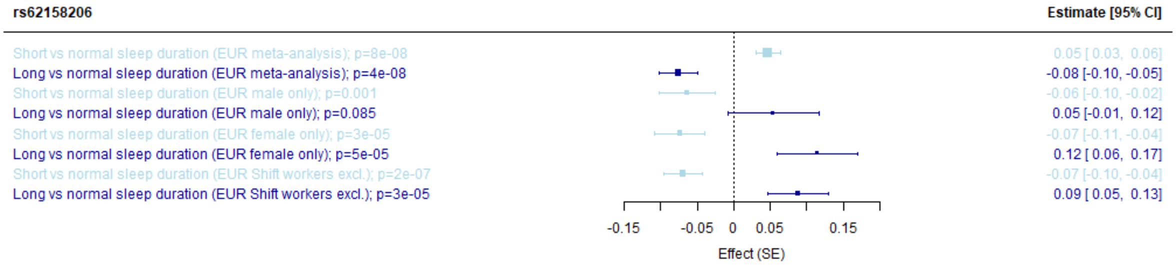
rs62158206
Estimate [95% CI]
Short vs normal sleep duration (EUR meta-analysis); p=8e-08
Long vs normal sleep duration (EUR meta-analysis); p=4e-08
Short vs normal sleep duration (EUR male only); p=0.001
Long vs normal sleep duration (EUR male only); p=0.085
Short vs normal sleep duration (EUR female only); p=3e-05
Long vs normal sleep duration (EUR female only); p=5e-05
Short vs normal sleep duration (EUR Shift workers excl.); p=2e-07
Long vs normal sleep duration (EUR Shift workers excl.); p=3e-05
0.05 [0.03, 0.06]
-0.08 [-0.10, -0.05]
-0.06 [-0.10, -0.02]
0.05 [-0.01, 0.12]
-0.07 [-0.11, -0.04]
0.12 [0.06, 0.17]
-0.07 [-0.10, -0.04]
0.09 [0.05, 0.13]
-0.15
-0.05
0
0.05
0.15
Effect (SE)

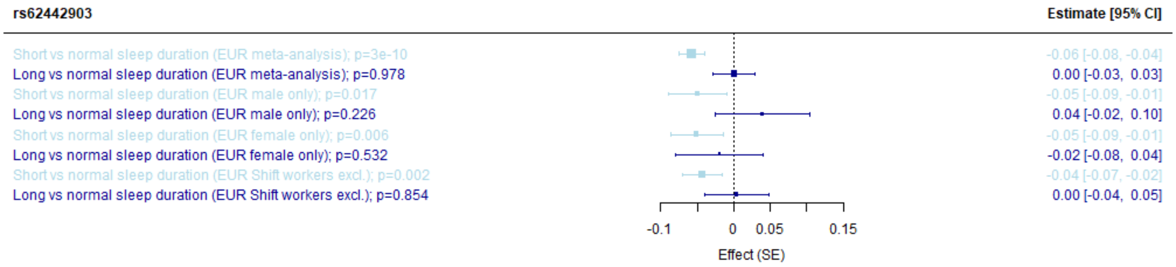
rs62442903
Estimate [95% CI]
Short vs normal sleep duration (EUR meta-analysis); p=3e-10
Long vs normal sleep duration (EUR meta-analysis); p=0.978
Short vs normal sleep duration (EUR male only); p=0.017
Long vs normal sleep duration (EUR male only); p=0.226
Short vs normal sleep duration (EUR female only); p=0.006
Long vs normal sleep duration (EUR female only); p=0.532
Short vs normal sleep duration (EUR Shift workers excl.); p=0.002
Long vs normal sleep duration (EUR Shift workers excl.); p=0.854
-0.06 [-0.08, -0.04]
0.00 [-0.03, 0.03]
-0.05 [-0.09, -0.01]
0.04 [-0.02, 0.10]
-0.05 [-0.09, -0.01]
-0.02 [-0.08, 0.04]
-0.04 [-0.07, -0.02]
0.00 [-0.04, 0.05]
-0.1
0
0.05
0.15
Effect (SE)

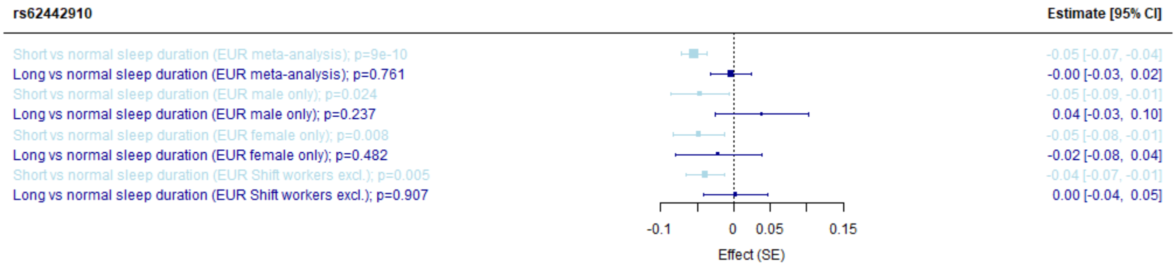
rs62442910
Estimate [95% CI]
Short vs normal sleep duration (EUR meta-analysis); p=9e-10
Long vs normal sleep duration (EUR meta-analysis); p=0.761
Short vs normal sleep duration (EUR male only); p=0.024
Long vs normal sleep duration (EUR male only); p=0.237
Short vs normal sleep duration (EUR female only); p=0.008
Long vs normal sleep duration (EUR female only); p=0.482
Short vs normal sleep duration (EUR Shift workers excl.); p=0.005
Long vs normal sleep duration (EUR Shift workers excl.); p=0.907
-0.05 [-0.07, -0.04]
-0.00 [-0.03, 0.02]
-0.05 [-0.09, -0.01]
0.04 [-0.03, 0.10]
-0.05 [-0.08, -0.01]
-0.02 [-0.08, 0.04]
-0.04 [-0.07, -0.01]
0.00 [-0.04, 0.05]
-0.1
0
0.05
0.15
Effect (SE)

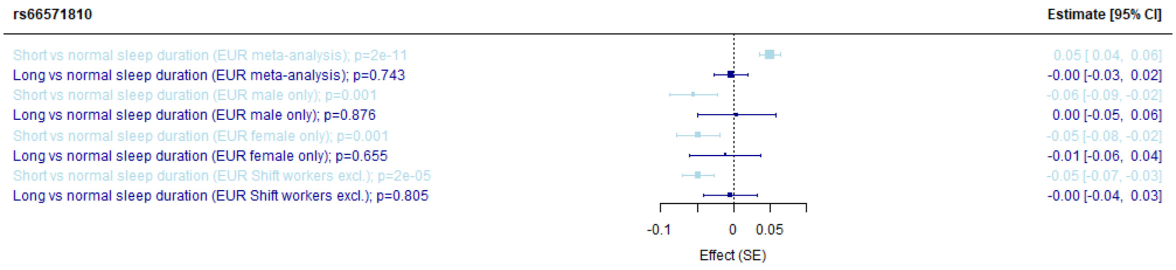
rs66571810
Estimate [95% CI]
Short vs normal sleep duration (EUR meta-analysis); p=2e-11
Long vs normal sleep duration (EUR meta-analysis); p=0.743
Short vs normal sleep duration (EUR male only); p=0.001
Long vs normal sleep duration (EUR male only); p=0.876
Short vs normal sleep duration (EUR female only); p=0.001
Long vs normal sleep duration (EUR female only); p=0.655
Short vs normal sleep duration (EUR Shift workers excl.); p=2e-05
Long vs normal sleep duration (EUR Shift workers excl.); p=0.805
0.05 [0.04, 0.06]
-0.00 [-0.03, 0.02]
-0.06 [-0.09, -0.02]
0.00 [-0.05, 0.06]
-0.05 [-0.08, -0.02]
-0.01 [-0.06, 0.04]
-0.05 [-0.07, -0.03]
-0.00 [-0.04, 0.03]
-0.1
0
0.05
Effect (SE)

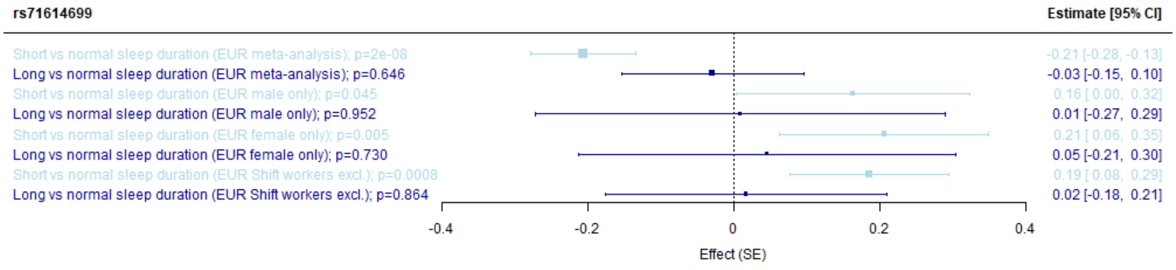
rs71614699
Estimate [95% CI]
Short vs normal sleep duration (EUR meta-analysis); p=2e-08
Long vs normal sleep duration (EUR meta-analysis); p=0.646
Short vs normal sleep duration (EUR male only); p=0.045
Long vs normal sleep duration (EUR male only); p=0.952
Short vs normal sleep duration (EUR female only); p=0.005
Long vs normal sleep duration (EUR female only); p=0.730
Short vs normal sleep duration (EUR Shift workers excl.); p=0.0008
Long vs normal sleep duration (EUR Shift workers excl.); p=0.864
-0.21 [-0.28, -0.13]
-0.03 [-0.15, 0.10]
0.16 [0.00, 0.32]
0.01 [-0.27, 0.29]
0.21 [0.06, 0.35]
0.05 [-0.21, 0.30]
0.19 [0.08, 0.29]
0.02 [-0.18, 0.21]
-0.4
-0.2
0
0.2
0.4
Effect (SE)

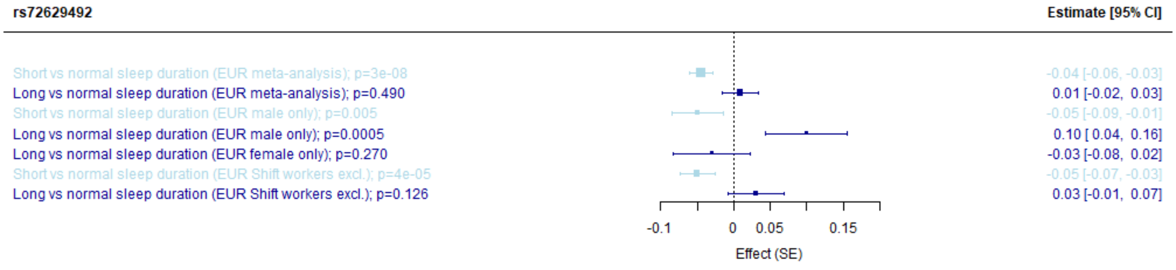
rs72629492
Estimate [95% CI]
Short vs normal sleep duration (EUR meta-analysis); p=3e-08
Long vs normal sleep duration (EUR meta-analysis); p=0.490
Short vs normal sleep duration (EUR male only); p=0.005
Long vs normal sleep duration (EUR male only); p=0.0005
Long vs normal sleep duration (EUR female only); p=0.270
Short vs normal sleep duration (EUR Shift workers excl.); p=4e-05
Long vs normal sleep duration (EUR Shift workers excl.); p=0.126
-0.04 [-0.06, -0.03]
0.01 [-0.02, 0.03]
-0.05 [-0.09, -0.01]
0.10 [0.04, 0.16]
-0.03 [-0.08, 0.02]
-0.05 [-0.07, -0.03]
0.03 [-0.01, 0.07]
-0.1
0
0.05
0.15
Effect (SE)

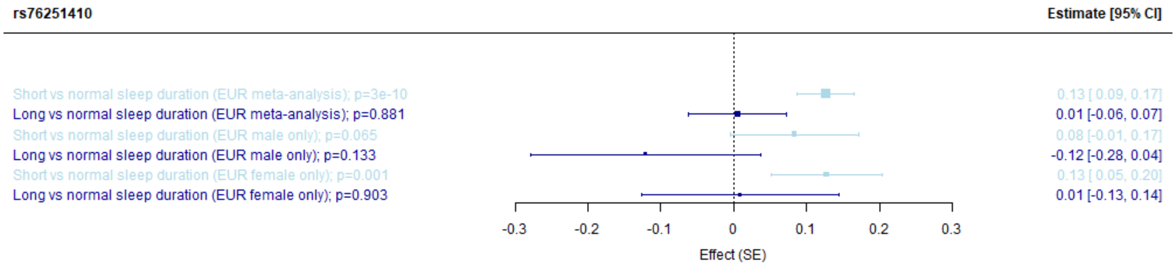
rs76251410
Estimate [95% CI]
Short vs normal sleep duration (EUR meta-analysis); p=3e-10
Long vs normal sleep duration (EUR meta-analysis); p=0.881
Short vs normal sleep duration (EUR male only); p=0.065
Long vs normal sleep duration (EUR male only); p=0.133
Short vs normal sleep duration (EUR female only); p=0.001
Long vs normal sleep duration (EUR female only); p=0.903
0.13 [0.09, 0.17]
0.01 [-0.06, 0.07]
0.08 [-0.01, 0.17]
-0.12 [-0.28, 0.04]
0.13 [0.05, 0.20]
0.01 [-0.13, 0.14]
-0.3
-0.2
-0.1
0
0.1
0.2
0.3
Effect (SE)

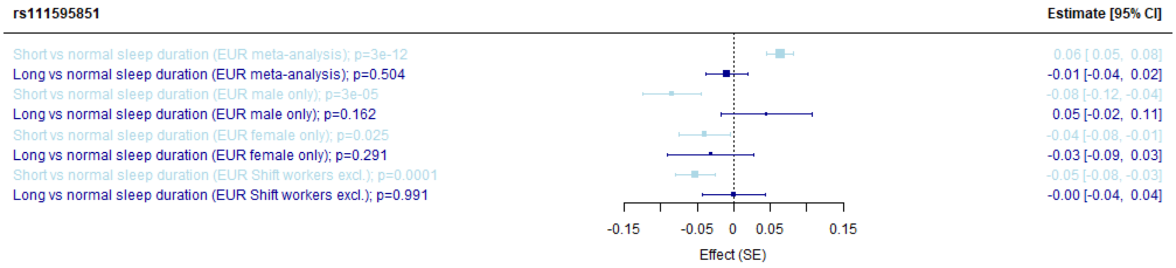
rs111595851
Estimate [95% CI]
Short vs normal sleep duration (EUR meta-analysis); p=3e-12
Long vs normal sleep duration (EUR meta-analysis); p=0.504
Short vs normal sleep duration (EUR male only); p=3e-05
Long vs normal sleep duration (EUR male only); p=0.162
Short vs normal sleep duration (EUR female only); p=0.025
Long vs normal sleep duration (EUR female only); p=0.291
Short vs normal sleep duration (EUR Shift workers excl.); p=0.0001
Long vs normal sleep duration (EUR Shift workers excl.); p=0.991
0.06 [0.05, 0.08]
-0.01 [-0.04, 0.02]
-0.08 [-0.12, -0.04]
0.05 [-0.02, 0.11]
-0.04 [-0.08, -0.01]
-0.03 [-0.09, 0.03]
-0.05 [-0.08, -0.03]
-0.00 [-0.04, 0.04]
-0.15
-0.05
0
0.05
0.15
Effect (SE)

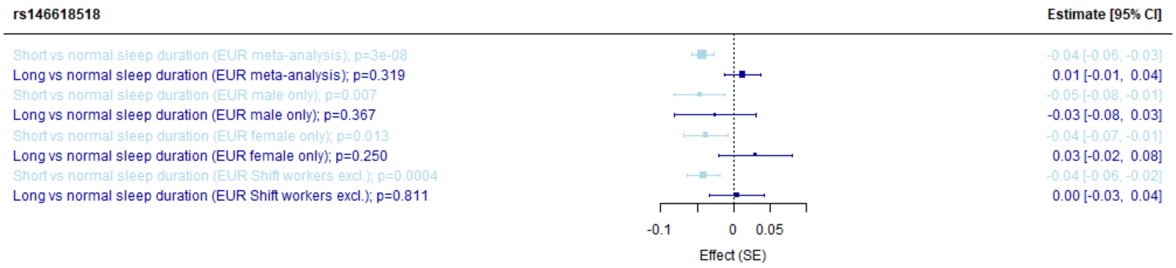
rs146618518
Estimate [95% CI]
Short vs normal sleep duration (EUR meta-analysis); p=3e-08
Long vs normal sleep duration (EUR meta-analysis); p=0.319
Short vs normal sleep duration (EUR male only); p=0.007
Long vs normal sleep duration (EUR male only); p=0.367
Short vs normal sleep duration (EUR female only); p=0.013
Long vs normal sleep duration (EUR female only); p=0.250
Short vs normal sleep duration (EUR Shift workers excl.); p=0.0004
Long vs normal sleep duration (EUR Shift workers excl.); p=0.811
-0.04 [-0.06, -0.03]
0.01 [-0.01, 0.04]
-0.05 [-0.08, -0.01]
-0.03 [-0.08, 0.03]
-0.04 [-0.07, -0.01]
0.03 [-0.02, 0.08]
-0.04 [-0.06, -0.02]
0.00 [-0.03, 0.04]
-0.1
0
0.05
Effect (SE)

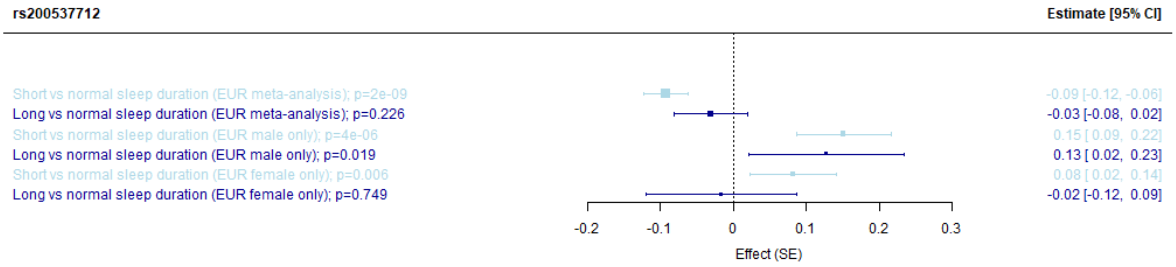
rs200537712
Estimate [95% CI]
Short vs normal sleep duration (EUR meta-analysis); p=2e-09
Long vs normal sleep duration (EUR meta-analysis); p=0.226
Short vs normal sleep duration (EUR male only); p=4e-06
Long vs normal sleep duration (EUR male only); p=0.019
Short vs normal sleep duration (EUR female only); p=0.006
Long vs normal sleep duration (EUR female only); p=0.749
-0.09 [-0.12, -0.06]
-0.03 [-0.08, 0.02]
0.15 [0.09, 0.22]
0.13 [0.02, 0.23]
0.08 [0.02, 0.14]
-0.02 [-0.12, 0.09]
-0.2
-0.1
0
0.1
0.2
0.3
Effect (SE)

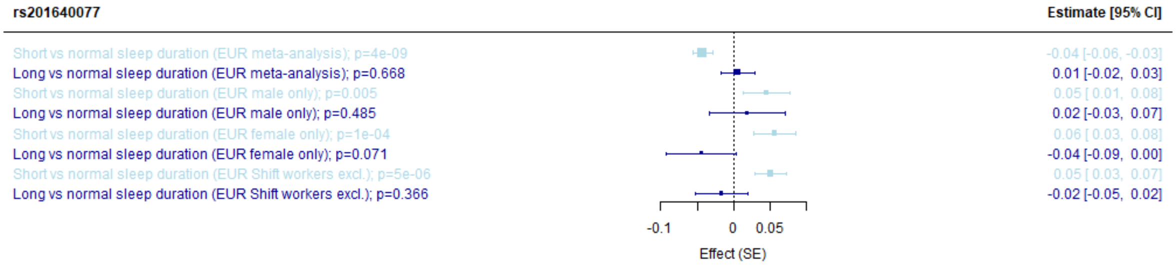
rs201640077
Estimate [95% CI]
Short vs normal sleep duration (EUR meta-analysis); p=4e-09
Long vs normal sleep duration (EUR meta-analysis); p=0.668
Short vs normal sleep duration (EUR male only); p=0.005
Long vs normal sleep duration (EUR male only); p=0.485
Short vs normal sleep duration (EUR female only); p=1e-04
Long vs normal sleep duration (EUR female only); p=0.071
Short vs normal sleep duration (EUR Shift workers excl.); p=5e-06
Long vs normal sleep duration (EUR Shift workers excl.); p=0.366
-0.04 [-0.06, -0.03]
0.01 [-0.02, 0.03]
0.05 [0.01, 0.08]
0.02 [-0.03, 0.07]
0.06 [0.03, 0.08]
-0.04 [-0.09, 0.00]
0.05 [0.03, 0.07]
-0.02 [-0.05, 0.02]
-0.1
0
0.05
Effect (SE)

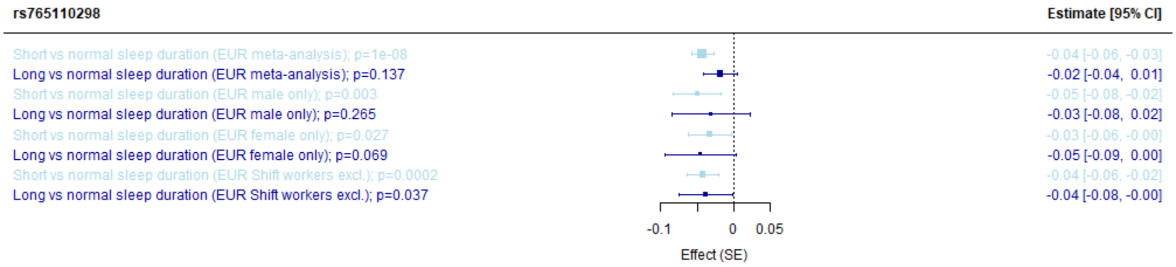
rs765110298
Estimate [95% CI]
Short vs normal sleep duration (EUR meta-analysis); p=1e-08
Long vs normal sleep duration (EUR meta-analysis); p=0.137
Short vs normal sleep duration (EUR male only); p=0.003
Long vs normal sleep duration (EUR male only); p=0.265
Short vs normal sleep duration (EUR female only); p=0.027
Long vs normal sleep duration (EUR female only); p=0.069
Short vs normal sleep duration (EUR Shift workers excl.); p=0.0002
Long vs normal sleep duration (EUR Shift workers excl.); p=0.037
-0.04 [-0.06, -0.03]
-0.02 [-0.04, 0.01]
-0.05 [-0.08, -0.02]
-0.03 [-0.08, 0.02]
-0.03 [-0.06, -0.00]
-0.05 [-0.09, 0.00]
-0.04 [-0.06, -0.02]
-0.04 [-0.08, -0.00]
-0.1
0
0.05
Effect (SE)

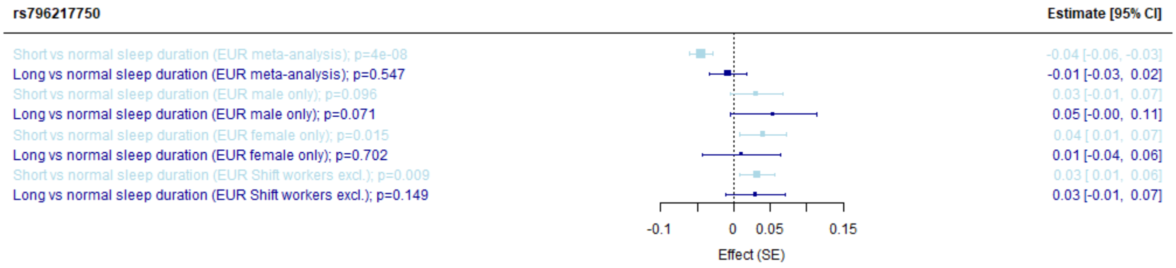
rs796217750
Estimate [95% CI]
Short vs normal sleep duration (EUR meta-analysis); p=4e-08
Long vs normal sleep duration (EUR meta-analysis); p=0.547
Short vs normal sleep duration (EUR male only); p=0.096
Long vs normal sleep duration (EUR male only); p=0.071
Short vs normal sleep duration (EUR female only); p=0.015
Long vs normal sleep duration (EUR female only); p=0.702
Short vs normal sleep duration (EUR Shift workers excl.); p=0.009
Long vs normal sleep duration (EUR Shift workers excl.); p=0.149
-0.04 [-0.06, -0.03]
-0.01 [-0.03, 0.02]
0.03 [-0.01, 0.07]
0.05 [-0.00, 0.11]
0.04 [0.01, 0.07]
0.01 [-0.04, 0.06]
0.03 [0.01, 0.06]
0.03 [-0.01, 0.07]
-0.1
0
0.05
0.15
Effect (SE)

6. Ingenuity Pathways Analysis: Results

The top significantly enriched canonical pathway for short sleep was DNA Methylation and Transcriptional Repression Signaling, followed by the Complement System, molecular mechanisms of cancer, caveolar-mediated endocytosis signaling and synaptogenesis signaling pathway. For long sleep the top findings were reelin signaling in neurons, semaphoring neuronal repulsive signaling pathway, breast cancer regulation by stathmin 1, cAMP-mediated signaling, and glutaryl-CoA degradation (supplementary table 28).

7. Replication of previously published significant associations in the MVP sample.

A previously published GWAS of sleep duration in European UK Biobank sample revealed several GWS risk loci for short sleep (<7 hours; 27 significant loci), long sleep (>8 hours; 8 significant loci) and sleep as a quantitative trait (from 0-24 hours) (78 significant loci) (14). Note that the definitions of short and long sleep differ slightly from our own. As noted in the main text, we did not assess the sleep data from MVP as a continuous trait. We used the European MVP-only GWAS to provide an independent replication sample for previously published GWAS assessing self-reported sleep duration as both a binary and quantitative trait in 446,118 European UK Biobank subjects[1] (7). For this analysis, we considered a locus replicated if it reached a nominal significance threshold of $p < 0.05$ in the MVP GWAS. Where SNPs were not present in MVP, we investigated SNPs in high LD ($r^2>0.8$) for replication, using 1000 Genomes Project European population as a reference panel. Where the primary study investigated sleep as a continuous trait, we considered a locus as replicated in our sample where the odds ratio was in the opposite direction for short sleep, and a consistent direction for long sleep.

Of the 27 significant loci for short sleep duration, 17 were present in our study. We were able to identify reliable LD proxies for seven additional SNPs, leaving a total of three that could not be assessed in our data. A total of 10 reach a significance threshold of at least $p < 0.05$ in an independent sample of 158,222 EUR subjects from the MVP GWAS for short sleep duration (with a more stringent definition of <6 hours sleep) with same effect direction. In addition, two loci were significantly associated with long sleep in our MVP data, with an opposite direction of effect to that observed in the primary study of short sleep duration (see supplementary tables 17 and 18).

Of the eight loci significant associated with long sleep in (14), five are present in our MVP data, and we were able to identify a reliable LD proxy for a further two. One locus, rs549961083 on chromosome five, could not be assessed. None of these seven loci were replicated in our study of long sleep duration. However, two of these loci (rs4585442 on chromosome 5, and rs1229762 on chromosome 7) are significantly associated with short sleep duration in the MVP sample, with an opposite direction of effect to that reported in the original study of long sleep (see supplementary tables 19 and 20)

In addition to considering long and short sleep as binary traits, Dashti *et al* (14) conducted a GWAS of sleep as a quantitative continuous measure, for which they identify 78 GWS loci. A total of 58 of these loci were present in our MVP data, and we identified reliable LD proxies for further 12. Eight of these loci could not be assessed in our data. Of those 78 significant associations with continuous sleep duration, we replicate a total of 18 of these associations in our MVP GWAS on short sleep, and three in our MVP GWAS on long sleep (see supplementary tables 21 and 22).

---

[1] Dashti HS, Jones SE, Wood AR, Lane JM, van Hees VT, Wang H, et al. Genome-wide association study identifies genetic loci for self-reported habitual sleep duration supported by accelerometer-derived estimates. Nat Commun. 2019;10(1):1100.
