# Supplementary material for: Genome-wide association studies and cross-population meta-analyses investigating short and long sleep duration
Source: Nat Commun. 2023 Sep 28;14:6059. doi: 10.1038/s41467-023-41249-y (PMC10539313; doi:10.1038/s41467-023-41249-y)
Supplement: Supplementary file 2 — Description of Additional Supplementary Files [file 41467_2023_41249_MOESM2_ESM.pdf]

Description of Additional Supplementary Files

File Name: Supplementary Data 1-41
Description: This excel file contains 41 additional tables and data to support the main text. A hyperlinked contents table describes the data contained within each tab. Each table is referenced in the main text and detailed legends are provided throughout.
