# Supplementary material for: Genome-wide association studies and cross-population meta-analyses investigating short and long sleep duration
Source: Nat Commun. 2023 Sep 28;14:6059. doi: 10.1038/s41467-023-41249-y (PMC10539313; doi:10.1038/s41467-023-41249-y)
Supplement: Supplementary file 4 — Reporting Summary [file 41467_2023_41249_MOESM4_ESM.pdf]

nature portfolio

Corresponding author(s): Joel Gelernter

Last updated by author(s): Jul 5, 2023

Reporting Summary

Nature Portfolio wishes to improve the reproducibility of the work that we publish. This form provides structure for consistency and transparency in reporting. For further information on Nature Portfolio policies, see our Editorial Policies and the Editorial Policy Checklist.

Statistics

For all statistical analyses, confirm that the following items are present in the figure legend, table legend, main text, or Methods section.

n/a	Confirmed	
☐	☒	The exact sample size (*n*) for each experimental group/condition, given as a discrete number and unit of measurement
☐	☒	A statement on whether measurements were taken from distinct samples or whether the same sample was measured repeatedly
☐	☒	The statistical test(s) used AND whether they are one- or two-sided<br>*Only common tests should be described solely by name; describe more complex techniques in the Methods section.*
☐	☒	A description of all covariates tested
☐	☒	A description of any assumptions or corrections, such as tests of normality and adjustment for multiple comparisons
☐	☒	A full description of the statistical parameters including central tendency (e.g. means) or other basic estimates (e.g. regression coefficient) AND variation (e.g. standard deviation) or associated estimates of uncertainty (e.g. confidence intervals)
☐	☒	For null hypothesis testing, the test statistic (e.g. *F*, *t*, *r*) with confidence intervals, effect sizes, degrees of freedom and *P* value noted<br>*Give P values as exact values whenever suitable.*
☒	☐	For Bayesian analysis, information on the choice of priors and Markov chain Monte Carlo settings
☒	☐	For hierarchical and complex designs, identification of the appropriate level for tests and full reporting of outcomes
☐	☒	Estimates of effect sizes (e.g. Cohen's *d*, Pearson's *r*), indicating how they were calculated

Our web collection on statistics for biologists contains articles on many of the points above.

Software and code

Policy information about availability of computer code

Data collection	This manuscript uses data from UK Biobank and The Million Veteran Program. Detailed information on the data collection for these studies is publicly available.
Data analysis	PCA analysis and relatedness were assessed using GENESIS version 2.30.0 (PC-AiR/PC-Relate respectively)<br>Genome wide association studies were conducted using Plink 2.0.<br>Meta-analyses of these primary GWAS studies was conducted using METAL (2011-03-25).<br>Genetic correlation analyses and SNP-based heritability were conducted using linkage disequilibrium score regression (LDSC v1.0.1) and covariate adjusted LDSC (cov-LDSC 2018-06-07) and POPCORN version 0.9.6.<br>Cross-population transferability of loci was conducted using the R package LDlinkR (v4.0).<br>Mendelian randomisation analyses were conducted using TwoSampleMR (v0.5.7) R package.<br>We uploaded summary statistics from the primary GWAS and the meta-analyses into the FUMA (Functional Mapping and Annotation) GWAS platform version 1.3.7, to examine gene-level associations using MAGMA (Multi-Marker Analysis of GenoMic Annotation) version 1.6.<br>SNP-level finemapping was conducted using PolyFun (POLYgenic FUNctionally-informed fine-mapping)<br>Functionally-informed fine-mapping was conducted using SuSiE (Sum of Single Effects) and we then estimated functional enrichment using S-LDSC (stratified LD-score regression)<br>TWAS was conducted with FUSION<br>We used FOCUS (Fine-mapping Of CaUsal gene Sets) to fine-map genomic risk regions<br>We performed a Core Analysis using Ingenuity Pathway Analysis software (December 2022)<br><br>All additional statistical analysis was conducted using R version 3.5.0.<br>Relevant references:

core Team R. R: A Language and Environment for Statistical Computing. R Found Stat Comput Vienna Austria. 2018;
Purcell S, Neale B, Todd-Brown K, Thomas L, Ferreira M, Bender D, et al. PLINK: a toolset for whole-genome association and population-based linkage analysis. Am J Hum Genet. 2007;81(3):559–75.
Willer CJ, Li Y, Abecasis GR. METAL: Fast and efficient meta-analysis of genomewide association scans. Bioinformatics. 2010;26(17):2190–1
Watanabe K, Taskesen E, Van Bochoven A, Posthuma D. Functional mapping and annotation of genetic associations with FUMA. Nat Commun. 2017;8:1826.
Myers TA, Chanock SJ, Machiela MJ. LDlinkR: An R Package for Rapidly Calculating Linkage Disequilibrium Statistics in Diverse Populations. Front Genet. 2020 Feb 28;11:157.
Auton A, Abecasis GR, Altshuler DM, Durbin RM, Bentley DR, Chakravarti A, et al. A global reference for human genetic variation. Nature. 2015.
Luo Y, Li X, Wang X, Gazal S, Mercader JM, 23 and Me Research Team, et al. Estimating heritability and its enrichment in tissue-specific gene sets in admixed populations. Hum Mol Genet. 2021 May 13;

For manuscripts utilizing custom algorithms or software that are central to the research but not yet described in published literature, software must be made available to editors and reviewers. We strongly encourage code deposition in a community repository (e.g. GitHub). See the Nature Portfolio guidelines for submitting code & software for further information.

Data

Policy information about availability of data

All manuscripts must include a data availability statement. This statement should provide the following information, where applicable:

- Accession codes, unique identifiers, or web links for publicly available datasets
- A description of any restrictions on data availability
- For clinical datasets or third party data, please ensure that the statement adheres to our policy

The summary statistics for the GWAS and meta-analyses generated in this study have been deposited in dbGAP under accession number phs001672.v1.p1 and are also available on the Gelernter Lab website (https://medicine.yale.edu/lab/gelernter/stats/). The UKB raw genotype data are available through UK Biobank (http://biobank.ndph.ox.ac.uk/showcase/). Data from the European Commission Photovoltaic Geographical Information System can be accessed here: https://re.jrc.ec.europa.eu/pvg_tools/en/. Data from the National Solar Radiation Database can be access here: https://nsrdb.nrel.gov/data-sets/how-to-access-data.

Human research participants

Policy information about studies involving human research participants and Sex and Gender in Research.

Reporting on sex and gender

All primary GWAS are adjusted for sex, as described in the manuscript. Sex was both self-reported and genetically inferred and participants with a mismatch between self-report and genetically inferred sex were excluded.

Population characteristics

We report sample characteristics for the UK Biobank and MVP cohorts within the manuscript. The UK Biobank is younger cohort than MVP (mean age UKB 56.8, mean age MVP 66.8). There are more female participants in UKB (54% female). The MVP sample is a US military veteran patient sample, with a majority of the participants being male (7% female). In both samples, the majority of participants are of European ancestry. Participants reporting 'normal' sleep of 7-8 hours were the largest groups in both cohorts, though there was a higher proportion of both short and long sleepers in the MVP cohort. Please see table 1 and figure 1 in the manuscript for further detail.

Recruitment

Recruitment was conducted by the UK Biobank and MVP studies and is described in greater detail in the following publications: Bycroft C, Freeman C, Petkova D, Band G, Elliott LT, Sharp K, et al. The UK Biobank resource with deep phenotyping and genomic data. Nature. 2018;
Sudlow C, Gallacher J, Allen N, Beral V, Burton P, Danesh J, et al. UK Biobank: An Open Access Resource for Identifying the Causes of a Wide Range of Complex Diseases of Middle and Old Age. PLoS Med. 2015;12(3).
Gaziano JM, Concato J, Brophy M, Fiore L, Pyarajan S, Breeling J, et al. Million Veteran Program: A mega-biobank to study genetic influences on health and disease. J Clin Epidemiol. 2016;70:214–23.

The UK Biobank is a population study that recruited approximately 500,000 participants across the UK, aged between 40 and 70. The main inclusion criteria were age between 40 and 70, living within reasonable distance from a UK Biobank assessment centre. MVP is a US military veteran sample, and all participants were required to be active users of the Veteran Health Administration with available electronic health record. All MVP and UK Biobank participants provided informed consent. All participants to both studies provided extensive lifestyle, medical, and genetic data thorough questionnaires and blood samples.

Ethics oversight

The UK Biobank study was approved by the North-West Research Ethics Committee (ref 06/MREC08/65) in accordance with the Declaration of Helsinki. Research involving the MVP in general is approved by the VA Central Institutional Review Board. All participants in both cohorts provided written informed consent.

Note that full information on the approval of the study protocol must also be provided in the manuscript.

Field-specific reporting

Please select the one below that is the best fit for your research. If you are not sure, read the appropriate sections before making your selection.

☒ Life sciences ☐ Behavioural & social sciences ☐ Ecological, evolutionary & environmental sciences

For a reference copy of the document with all sections, see nature.com/documents/nr-reporting-summary-flat.pdf

Life sciences study design

All studies must disclose on these points even when the disclosure is negative.

Sample size	Sample size was determined by inclusion of participants for whom we had the necessary sleep data, and who passed all relevant quality control checks. We aimed to be as inclusive as possible.
Data exclusions	We excluded all subjects with a ICD-10 diagnosis of sleep apnoea, as these were significantly over-represented in both the long and short sleep groups. We also excluded any subjects that could not be assigned to one of the main ancestry groups included, as the methods used in this study are not reliable where ancestry is undetermined.
Replication	We describe a replication of a previously published sleep GWAS in the supplementary material. The previous sleep GWAS (Dashti et al, 2019) used UK Biobank data, so we conducted a replication study in the independent MVP sample. Of the 27 significant loci for short sleep duration reported in Dashti et al (2019), 17 were present in our study. We were able to identify reliable LD proxies for seven additional SNPs, leaving a total of three that could not be assessed in our data. A total of 10 reach a significance threshold of at least $p < 0.05$ in an independent sample of 158,222 EUR subjects from the MVP GWAS for short sleep duration (with a more stringent definition of <6 hours sleep) with same effect direction. In addition, two loci were significantly associated with long sleep in our MVP data, with an opposite direction of effect to that observed in the primary study of short sleep duration (see supplementary tables 17 and 18).<br>Of the eight loci significant associated with long sleep in Dashti et al (2019), five are present in our MVP data, and we were able to identify a reliable LD proxy for a further two. One locus, rs549961083 on chromosome five, could not be assessed. None of these seven loci were replicated in our study of long sleep duration. However, two of these loci (rs4585442 on chromosome 5, and rs1229762 on chromosome 7) are significantly associated with short sleep duration in the MVP sample, with an opposite direction of effect to that reported in the original study of long sleep (see supplementary tables 19 and 20)<br>In addition to considering long and short sleep as binary traits, Dashti et al (2019) conducted a GWAS of sleep as a quantitative continuous measure, for which they identify 78 GWS loci. A total of 58 of these loci were present in our MVP data, and we identified reliable LD proxies for further 12. Eight of these loci could not be assessed in our data. Of those 78 significant associations with continuous sleep duration, we replicate a total of 18 of these associations in our MVP GWAS on short sleep, and three in our MVP GWAS on long sleep (see supplementary tables 21 and 22).
Randomization	Subjects were assigned to groups based on their self-reported sleep duration. All analyses were adjusted for age, sex, recruitment site. Randomisation was not relevant to this study design.
Blinding	Blinding was not relevant to this study as participants were recruited regardless of disease status or other characteristics.

Reporting for specific materials, systems and methods

We require information from authors about some types of materials, experimental systems and methods used in many studies. Here, indicate whether each material, system or method listed is relevant to your study. If you are not sure if a list item applies to your research, read the appropriate section before selecting a response.

Materials & experimental systems

n/a	Involved in the study	
☒	☐	Antibodies
☒	☐	Eukaryotic cell lines
☒	☐	Palaeontology and archaeology
☒	☐	Animals and other organisms
☒	☐	Clinical data
☒	☐	Dual use research of concern

Methods

n/a	Involved in the study	
☒	☐	ChIP-seq
☒	☐	Flow cytometry
☒	☐	MRI-based neuroimaging
